# Opto-Electrostatic Determination of Nucleic Acid Double-Helix Dimensions and the Structure of the Molecule–Solvent Interface

**DOI:** 10.1021/acs.macromol.2c00657

**Published:** 2022-07-01

**Authors:** Maria Bespalova, Ali Behjatian, Narain Karedla, Rowan Walker-Gibbons, Madhavi Krishnan

**Affiliations:** Physical and Theoretical Chemistry Laboratory, Department of Chemistry, University of Oxford, South Parks Road, Oxford OX1 3QZ, U.K.

## Abstract

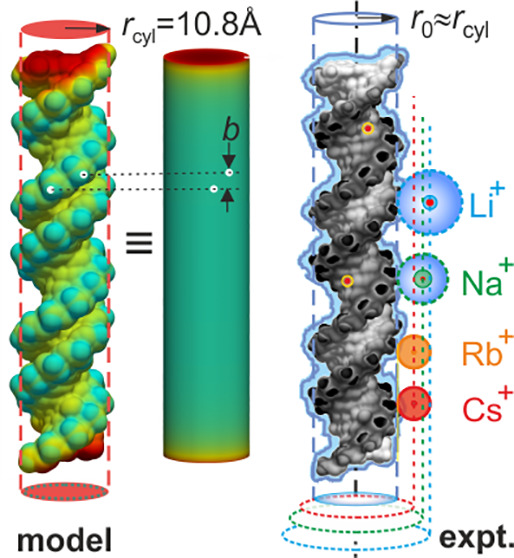

A DNA molecule is highly electrically charged in solution. The electrical potential at the molecular surface is known to vary strongly with the local geometry of the double helix and plays a pivotal role in DNA–protein interactions. Further out from the molecular surface, the electrical field propagating into the surrounding electrolyte bears fingerprints of the three-dimensional arrangement of the charged atoms in the molecule. However, precise extraction of the structural information encoded in the electrostatic “far field” has remained experimentally challenging. Here, we report an optical microscopy-based approach that detects the field distribution surrounding a charged molecule in solution, revealing geometric features such as the radius and the average rise per basepair of the double helix with up to sub-Angstrom precision, comparable with traditional molecular structure determination techniques like X-ray crystallography and nuclear magnetic resonance. Moreover, measurement of the helical radius furnishes an unprecedented view of both hydration and the arrangement of cations at the molecule–solvent interface. We demonstrate that a probe in the electrostatic far field delivers structural and chemical information on macromolecules, opening up a new dimension in the study of charged molecules and interfaces in solution.

Nucleic acids play a central role in biological function. Investigation of the structure of nucleic acids has had a long and compelling history and continues to have far-reaching impact in fields ranging from molecular biology, genetics and disease, to nanotechnology. A range of powerful techniques such as X-ray crystallography, nuclear magnetic resonance (NMR), atomic force microscopy (AFM), small-angle X-ray scattering (SAXS), Forster resonance energy transfer, and optical trapping have generated an unprecedented structural view of DNA, covering all length scales from the atomic to the macroscopic polymer contour level.^1−8^ The structural properties and function of this biopolymer in solution are strongly governed not only by steric and mechanical aspects but also by electrostatic considerations, as it is among the most highly charged linear polymers known.^9,10^ Indeed, electrical mobility measurements provided an early demonstration of the link between nucleic acid electrostatics and double helix geometry and molecular topology.^11^ More recently, magnetic tweezers and SAXS have been used to infer molecular properties of nucleic acids via the measurement of an intra- or intermolecular interaction potential.^12,13^ Furthermore, anomalous SAXS and atomic emission spectroscopy (AES) have probed the properties of the counterion atmosphere enveloping nucleic acid molecules,^14,15^ while X-ray photoelectron spectroscopy (XPS) has shed light on the interface between a charged nanoparticle and the surrounding electrolyte.^16^ To our knowledge, the ability to glean structural information on a diffusing macromolecule and its interface with the electrolyte through precise measurement of the electrical repulsion due to the molecule has not been demonstrated.

## Experimental Approach

**Figure 1:**
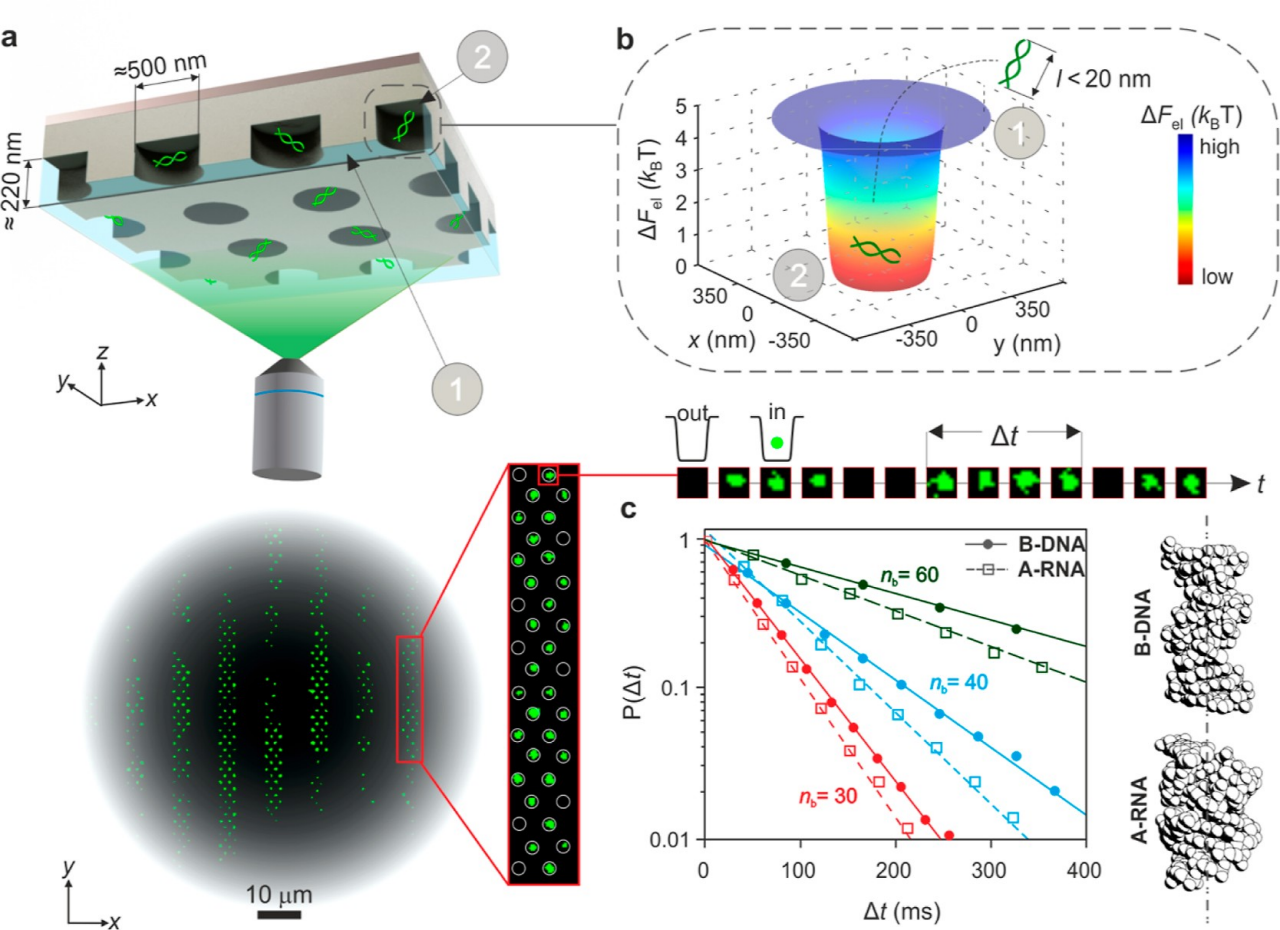
High-precision ET*e* measurements on nucleic acid fragments. (a) Schematic representation (not-to-scale) of fluorescently labeled nucleic acid molecules confined in an array of electrostatic fluidic traps and imaged using wide-field optical microscopy (top). Maximum intensity projection of 500 fluorescence images of parallel arrays of ≈700 traps imaged for 20 s (bottom). (b) Calculated spatial distribution of minimum axial electrostatic free energy, , in a representative trap (top). Labels “1” and “2” denote locations of the molecule outside and inside the potential well, respectively, and refer to spatial locations in the trapping nanostructure depicted in the device schematic in (a). A time course of optical images in a single trap (bottom) displays the duration of a single recorded residence event of duration, Δ*t*. (c) Probability density distributions, *P*(Δ*t*), of escape times, Δ*t*, for *N* = 10^4^ escape events for measurements on double-stranded B-DNA (solid lines) and A-RNA (dashed lines) in 1.23 mM LiCl for fragment length *n*_b_ = 30 (red), 40 (blue), and 60 (green) basepairs fitted to the expression . In order to enable comparison across different molecular species, *P*(Δ*t*) data series are rescaled such that the maximum value is 1. Average escape times, *t*_esc_, and measured effective charge values, *q*_m_, are as follows: *t*_esc,30B_ = 52.2 ± 0.3 ms (*q*_m,30B_ = −25.28 ± 0.07*e*), *t*_esc,40B_ 93.9 ± 0.4 ms (30.46 ± 0.06*e*), and  242.5 ± 1.1 ms (40.71 ± 0.07*e*) for B-DNA and  46.3 ± 0.2 ms (−23.86 ± 0.04*e*),  70.4 ± 0.8 ms (−28.35 ± 0.13*e*), and  192.5 ± 0.6 ms (−37.26 ± 0.04*e*) for A-RNA. B-DNA systematically displays 10–20% longer escape times and higher magnitudes of effective charge than A-RNA. Space filling structures of B-DNA and A-RNA reproduced with permission from ref (3) (right).

We optically visualize and measure the strength of electrostatic repulsions between a charged molecule and like-charged probe surfaces in solution using wide-field fluorescence microscopy and the recently developed escape time electrometry (ET*e*) approach.^17^ In contrast to scanning probe techniques where a nanoscale entity is placed in near contact with a stationary object of interest, our experiment involves a pair of flat, featureless probe surfaces placed in the “far field” of a diffusing charged molecular species in solution. We qualitatively define the electrostatic “far field” as the region in the electrolyte at a distance greater than a Debye length, , from the object. Here,  nm is a length scale governing the decay of electrostatic interactions in aqueous solution at temperature *T* = 298 K, where the salt concentration in solution, *c* ≈ 1–1.5 mM in this work, implies  8 nm. ET*e* measures the reduction in system free energy associated with transferring a charged molecule from a gap between like-charged parallel plates into a nanostructured “trap” region of very weak confinement where the molecule–plate repulsion is negligible^18^ (Figure 1a,b). The system is at thermodynamic equilibrium, and there are no externally applied fields. We create an array of such electrostatic fluidic traps using periodic nanostructured indentations in one surface of a parallel plate slit composed of silica surfaces separated by a gap of typical height, 2*h* = 75 nm. We introduce nucleic acid molecules at a concentration of 50–100 pM labeled with exactly two fluorescent dye molecules of ATTO 532, suspended in 1 mM Tris buffer and ≈1.2 mM monovalent salt solution, pH 9, into a system with multiple parallel lattices of traps (Figure 1a). Alkaline pH in the experiment ensures that the weakly acidic SiO_2_ walls of our nanoslit system are strongly charged.^19^ A low (mM) concentration of monovalent salt, in turn, ensures that the electrostatic interactions between a charged molecule and the walls of the slit are sufficiently strong and long-ranged, yielding long-lived trap states of ≈50–200 ms duration. Analytical characterization of the molecular species in the study using, for example, circular dichroism spectroscopy verifies that the solution conditions in our measurement conditions do not perturb the molecules’ structural integrity (see Supporting Information Figure S2).

Imaging the escape dynamics of trapped single molecules permits us to identify individual molecular residence events of duration Δ*t* in each trap. Photobleaching of the fluorescent dyes and any potential impact thereof on the measurement have been carefully explored in previous work.^17^ Because molecular residence times in the trap are much shorter than dye photobleaching times, we expect dye photophysics and photochemistry not to influence the accuracy of our measured escape times. Overdamped escape of an object from a potential well can be treated as a Poisson process with residence times that are exponentially distributed.^20^ Fits of the measured probability density function of residence times, *P*(Δ*t*), to an exponential function of the form  permit us to extract precise measurements of the molecular species’ average time to escape,  (Figure 1b,c). The average escape time, in turn, is expected to depend exponentially on well depth, according to the relation ,^20^ permitting us to relate measured  values to the depth of the trap, *W*, in the regime of *W* > 4*k*_B_*T*. In practice, we use Brownian dynamics (BD) simulations of the escape process in order to accurately convert measurements of  to the well depth, *W*, as described previously^17,21,22^ (Supporting Information Section S2).

In our BD simulations, we treat molecules as effective spheres of a radius equal to the measured hydrodynamic radius of the molecule. The hydrodynamic radius, *r*_H_, of each molecular species was measured using fluorescence correlation spectroscopy as described in the Supporting Information (Supplementary Methods). The use of an effective hydrodynamic radius, which ignores the anisotropic diffusive behavior of non-spherical objects, is valid when the translational diffusive length scale of interest, *l*_s_, is much larger than the length of the molecule, *l*, or in other words, when the ratio of rotational and translational diffusive timescales . For a rigid cylinder of length *l*, this ratio is approximately *l*^2^/*l*_s_^2^ (see ref (23)). The relevant length scale for translational diffusion, *l*_s_, in an ET*e* measurement corresponds to the radius of a nanostructured pocket which is typically 250–300 nm. Given the contour length of a 60 bp B-DNA (*l* ≈ 20 nm), which is the longest fragment considered here, we have *l*^2^/*l*_s_^2^ ≈ 0.01 ≪ 1, which ensures that the translational diffusion of an anisometric object may be treated as equivalent to that of an effective sphere for large displacements. It is worth noting that we ignore inertial effects in our BD simulations on the grounds that the momentum relaxation time of the molecule is very small.^24^ Although inertial BD simulations of large supercoiled DNA plasmids (∼1000 bp) have shown that mass can have some effects on conformation transition rates in equilibrium, they do also demonstrate that the translational diffusion coefficient of these molecules is accurately captured by conventional BD simulations.^24,25^ Thus, for short nucleic acid fragments, which are expected to behave like rigid rods, BD simulations in the overdamped regime are expected to provide an accurate description of our escape time problem.

The highly non-linear dependence of the measurand (escape time, ) on the measurable (well depth, *W*) facilitates precise interaction energy measurements. Observation of a large number of escape events, *N* ≈ 10^4^, reduces the fractional statistical uncertainty in the determination of *W* to about 0.1%.^22^ Importantly, the dominant contribution to the trap depth, *W*, is the electrostatic free energy of interaction, , which has robust theoretical underpinnings in the Poisson–Boltzmann (PB) framework for solution phase electrostatics as discussed further later.^26−28^ Correction of a contribution from axial spatial fluctuations to the total free energy, *W*, permits us to determine  with high precision, as described further in Supporting Information, Section S2. We have previously shown that  may be regarded in terms of the product of the effective charge of the molecule in solution, , and the electrical potential, ϕ_m_, at the midplane of the slit, such that  = .^29,30^ If ϕ_m_ is accurately known, the measurable in our experiment is the effective charge, , of the molecular species under the experimental conditions. Note that our values of  for charged spheres and cylinders are comparable to those encountered in other charge renormalization theories.^30−32^ Furthermore, our interaction-energy-based definition of  (i.e.,  = ) is identical to that in Kjellander’s dressed ion theory.^33−36^

The principle behind the present study may be summarized as follows: Accurate measurements of the electrostatic free energy,  permit us to measure the effective charge, , of three different lengths of a nucleic acid species (e.g., A- or B-form helix in this work). Theoretically expected effective charge values may also be calculated using the PB theoretical framework for each length of the fragment, as described previously (see Supporting Information Section S7).^30^ As described further below, calculations show that  depends strongly on geometrical dimensions of the molecular species of interest, for example, the rise per basepair, *b*, and helical radius, *r*. The precise functional form of this dependence is itself a function of the length of each fragment, as shown in Figures 3a and S4a. Thus, we have three independent theoretical relationships relating effective charge with molecular geometry for the fragment lengths under consideration. Since the effective charge of the molecular conformation under study (e.g., either the A-form or the B-form helix) may be described by a *common pair* of underlying geometric parameters (e.g., rise per basepair, *b*, and helical radius, *r*), a comparison of the measured effective charge values with the theoretically expected values for the three lengths of the double helix permits us to extract estimates of the two geometric properties of interest (described in detail in Supporting Information, Section S4). The third parameter we extract from the analysis characterizes the measurement device. We find that measurements of the helical radius in electrolytes containing cations of different radii further permit us to make inferences on the structure of the molecular interface with the electrolyte.

**Figure 2:**
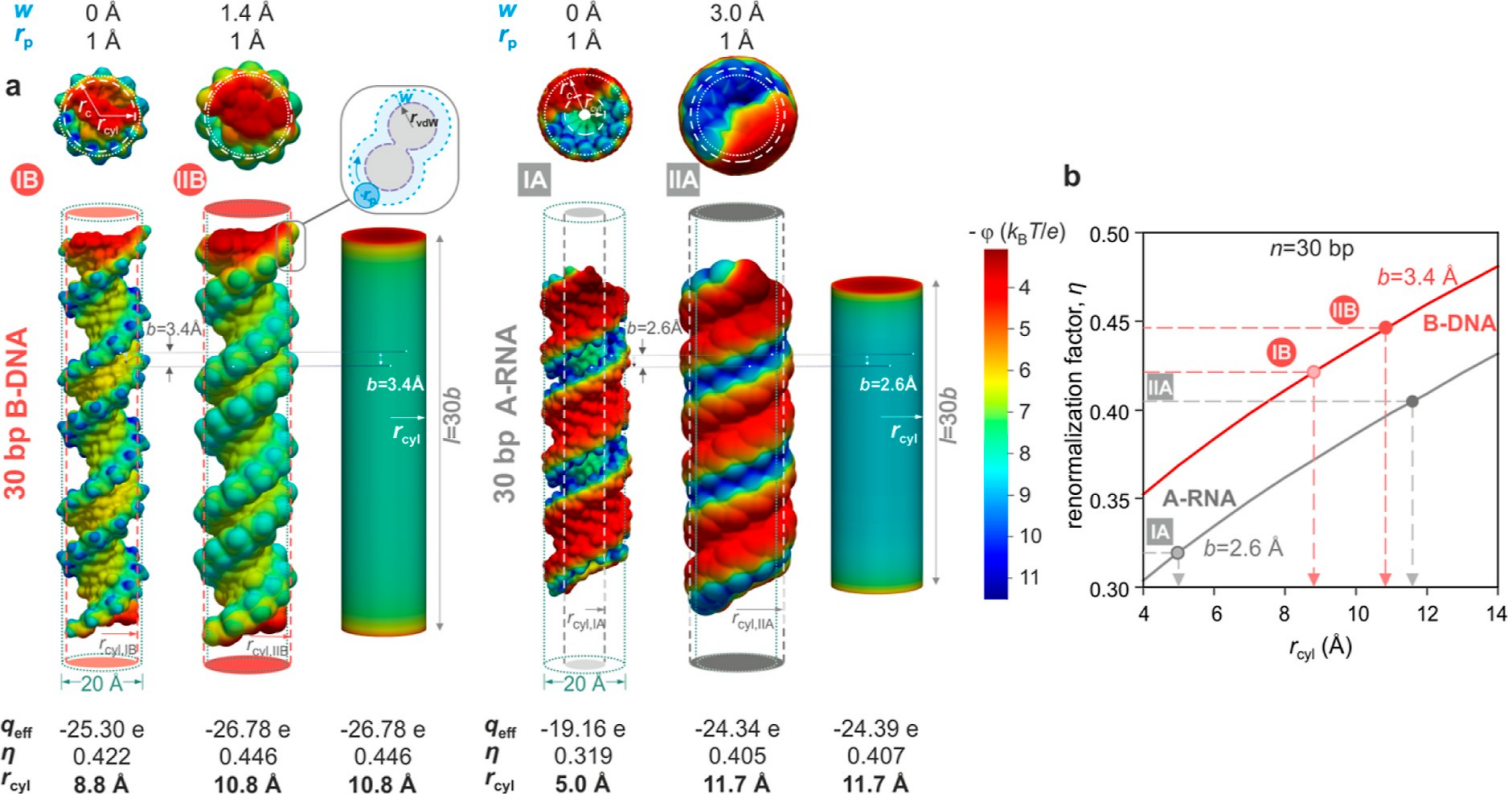
Modeling the double helix as a smooth charged cylinder of finite length. (a) Distributions of surface electrostatic potential, ϕ, for two molecular models of a 30 bp fragment of B-DNA (IB and IIB—left) and A-RNA (IA and IIA—right) generated based on atomic coordinates with rolling probe radius (*r*_p_ = 1 Å) and solvent accessible surface (*w*) parameter values as listed and pictured (inset) alongside axial projections of the molecular models (top panel). Surface potential distributions for corresponding smooth charged cylinders equivalent to models IIB and IIA carrying a total charge 60*e* with radii,  10.8 Å and  11.7 Å, respectively, and length 30*b* Å in each case. The radius of the equivalent cylinder,  (dashed lines), may be compared with a nominal double-helical radius *r*_c_ = 10 Å (dotted lines). (b) Calculated trends for the renormalization factor, , for cylinders of radius and length 30*b* Å, with nominal values of *b* = 3.4 Å for B-DNA (red line) and 2.6 Å for A-RNA (gray line). η values for the four molecular models can be related to those for smooth cylinders and correspond to  8.8 Å(effective vdW surface),  10.8 Å (effective SAS),  5 Å (vdWS), and  11.7 Å (SAS), two of which are depicted in (a). Panels are reproduced from ref (48), with the permission of AIP Publishing.

**Figure 3:**
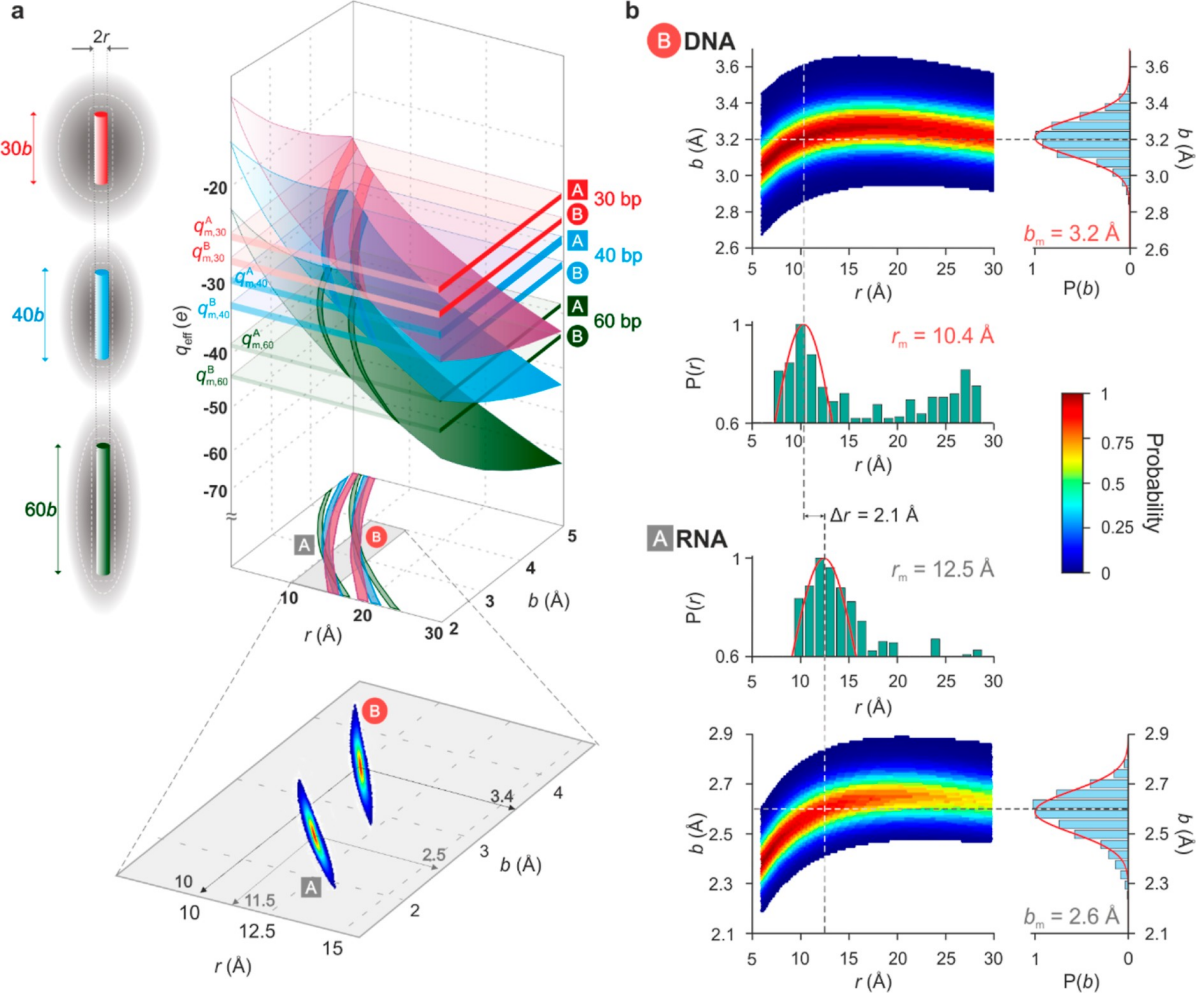
Measuring the helical rise per basepair and radius of the double helix. (a) Principle behind the measurement of the helical rise per basepair, *b*, and radius, *r*, of the double helix, for an ideal experiment, free of systematic measurement uncertainty (i.e., *f*_M_ = 1). Schematic representations of three lengths of a double-stranded nucleic acid species surrounded by a cloud of screening counterions (left). A measured value  for each molecular species of length *n* bp, in conjunction with the corresponding calculated 2D function (colored surface) for the effective charge, , generates a curve of possible solutions in *b* and *r*. Intersection of three such curves for *n* = 30, 40, and 60 bp yields a probability-weighted manifold of solutions from which measured values, *b*_m_ and *r*_m_, for the rise and radius, respectively, of each helix form can be obtained. (b) Measured *b*–*r* probability manifolds for B-DNA (top) and A-RNA (bottom) for an experiment performed in 1.2 mM CsCl. Since *f*_M_ ≠ 1 in experiments, measured *b*–*r* manifolds are broader than those in the ideal case depicted in (a) yielding  3.2 Å and  10.4 Å and  2.6 Å and  12.5 Å for B-DNA and A-RNA, respectively.

For a highly charged molecule in solution, it has been shown that , where η is a molecular geometry-dependent charge renormalization factor.^30−32,37,38^ denotes the net electrical charge in the molecular structure and stems from the sum of charge carried by the ionized structural groups and bound ions from the electrolyte. A highly acidic molecule like DNA, *n* basepairs in length and carrying a chemical modification at both 5′-end phosphates, has a structural charge  at pH 7 and higher (see Supporting Information, Section S3.2). Here, *e* is the elementary charge and  is the amount of charge due to the backbone phosphate groups on the molecule which are all fully ionized in our experiments. However, if a number of positively charged counterions, δ, associate with the molecule, for example, via energetic interactions beyond the purely Coulombic that are already accounted for within the PB model, then , where  is an inverse ion affinity parameter which tends to zero as .

To a first approximation, a periodic linear charged structure such as a short fragment of a double-stranded nucleic acid may be viewed as a smooth, charged cylinder of finite length.^39,40^ Here, η depends on the charge density of the polyelectrolyte and therefore on the axial base spacing, *b*, and the radius of the polyelectrolyte backbone, *r*. Considering a short stretch of a nucleic acid whose contour length, *l* = *nb*, is of the order of the Debye length, η further depends on *l*.^17,41,42^ Upon approximating a short stretch of DNA (≤60 bp) by a rigid cylinder of radius *r* and length *l*, we thus have  which can be calculated for a range of *b* and *r* values using the PB framework (Figures 2 and 3).^29,30^ Finally, for a given molecular geometry and structural charge, η is essentially independent of ion affinity for  0.7. Although η does exhibit some dependence on the salt concentration, *c*, this variation is negligible over the small range in experimental uncertainty in *c* in a given measurement.^30,31^

In view of the grooved molecular surface of double-stranded nucleic acids and the helicoid distribution of charge on the molecular backbone, we first test the quality of the smooth cylinder electrostatic model for DNA in the context of our experiment (Figure 2). We calculate  and therefore determine  values for molecular models of the full 3D structure of 30 bp B-DNA and A-RNA molecules constructed using the 3DNA platform (Supporting Information Section S7).^43^ We then determine  values for smooth cylinders of variable radii, *r*, and the same axial rise per basepair, *b*, as the molecular helices. Cylinders of radius  whose  values are identical to those of the molecular helices within computational error (estimated at <0.1%) are termed equivalent cylinders. Physically, this means that the computed electrostatic free energy difference between the "free" and "trapped" states (states1 and 2 in Fig. 1b respectively)of the molecular helix, , is indistinguishable from that due to a smooth cylinder of radius . Importantly, a domain decomposition of the free energy in the system demonstrates that the electrostatic well depth of the trap, , stems in nearly equal proportions from the “near field” (the region within about 2 nm) of both the molecule and the slit surfaces^48^. Note that high-resolution structural studies have shown that the double helix can have local structural variability, for example, sequence-dependent and thermally induced variation of the rise per basepair along the molecular contour, which is not captured in the uniformly charged cylinder model.^44−47^ Our approach measures an averaged interaction response from the molecule. Whereas thermal variations are expected to average out in the measurement, local sequence-dependent variations will be interpreted in terms of an *average* rise per basepair parameter characterizing the molecule. Therefore, for the current work, we assume that a coarse-grained model that treats the double helix as a uniformly charged cylinder provides a sufficient description of the measurement. Although mapping of the molecular problem on to that of a uniform cylinder can be highly informative, future work could directly compare electrometry measurements with expectations for molecular structural models.

We considered two molecular models each for B-DNA and A-RNA, with all molecular surfaces generated using rolling probe radii, *r*_p_ = 1 Å. Models-IA and -IB were generated using reference van der Waals (vdW) values for all atoms, while models-II A and -IIB entail atomic radii that are all *w* = 3 Å and *w* = 1.4 Å larger than the vdW values, respectively (Figure 2a). While model-I is expected to capture the vdW surface (vdWS) of the molecule, a larger atomic radius in model-II is expected to mimic a “solvent accessible surface” (SAS) which defines the distance of closest approach of the center of a water molecule to the macromolecular structure. For B-DNA, we find that models-I and -II yield equivalent electrostatic cylinder radii,  9 Å and 11 Å, respectively, which are in remarkable agreement with the nominal outer helical radius, *r*_c_ ≈ 10 Å, inferred from molecular crystal structures (Figure 2b).^28^ Interestingly, for A-RNA, the  values for the two structures considered are rather different:  5 Å and  12 Å, suggesting that an experimental measurement with sufficient accuracy may be able to distinguish between the two models, shedding light on molecular interfacial structural detail in an electrolyte (Supporting Information Section S7). The modeling procedure has been described in detail previously^48^ and is summarized in Supporting Information Section S7.

Precise measurements (uncertainty<1%) of  on three nucleic acid fragments of different lengths may be compared with calculated  values for charged cylinders in order to extract measures of three unknown quantities of interest (Figure 3a). Two of these three unknowns describe geometric properties of the underlying molecular structure, namely, the radius of the helix, *r*, and the axial helical rise per basepair, *b*. The third unknown relates to experimental measurement conditions and the associated uncertainty. Experiments generally contain parameters that need to be well controlled, or accounted for, in order to foster accurate measurements. We account for uncertainties in various experimental quantities through the use of two correction terms: one is a multiplicative factor, *f*_M_, and the other is an additive quantity, *f*_A_, such that the measured effective charge for each fragment of size *n* bases is given by . The correction factor, *f*_M_ = *f*_ion_^R^*f*_ϕ_, accounts for effects that influence the measured effective charge in a multiplicative fashion and is, in turn, composed of two terms. *f*_ϕ_ reflects a property of the measurement apparatus and involves the overall uncertainty in the midplane electrical potential, ϕ_m_, in the slit. ϕ_m_ directly relates to the effective surface potential of the silica surfaces, ϕ_s_, via the relation ϕ_m_ = 2ϕ_s_ exp(−*h*), and we use a nominal value of ϕ_s_ = −2.8 for our experimental conditions as noted in previous work.^21^ Examples of factors that contribute to variations in *f*_ϕ_ include the finite accuracy of the order of *h*_e_ ≈ 1 nm in the height of the slit, the particular value of the surface charge density on the confining walls, the salt concentration, and possible ionic species effects on ϕ_s_. *f*_ion_^R^, in turn, represents a relative "inverse affinity" of cations for the nucleic acid molecule, measured with respect to Na^+^ ions, such that *f*_Na_^R^ = 1. Finally, *f*_A_ is an additive term, the main contribution to which is 0.5*e*, the effective charge of the fluorescent label covalently coupled to both 5′-phosphates of the double helix, which is determined by measurement (see Supporting Information, Section S3).

We constructed 30, 60, and 40 bp fragments of dsDNA and dsRNA and measured the effective charge for each molecular species. We then compared the measured effective charge values, *q*_m_, with the corresponding calculated values, , for cylinders with linear charge spacing corresponding to rise per basepair values, *b*, ranging from 2 to 5 Å and the radius, *r*, in the range of 6–30 Å. In principle, simultaneously solving the three known relationships for  with  for the three fragments should yield values for the unknowns *b* and *r* when *f*_M_ = 1 (Figure 3a). However, in general, *f*_M_ ≠ 1, and the measurement data, which are of the form , are not single-valued but rather carry Gaussian-distributed uncertainties of width  about the mean value, *q*_m_. Thus, we have three functions of the form  Pairwise division of these three equations eliminates *f*_M_ and results in two functions that may be numerically solved to yield a probability-weighted manifold of solutions in *b* and *r* (Figure 3b). We determine the most probable measured values *b*_m_ and *r*_m_ using an algorithm developed based on simulated input data. *f*_M_ is then determined self-consistently by substitution into one of the three equations for  (Supporting Information Figure S4 and Section S4).

## Results

**Figure 4:**
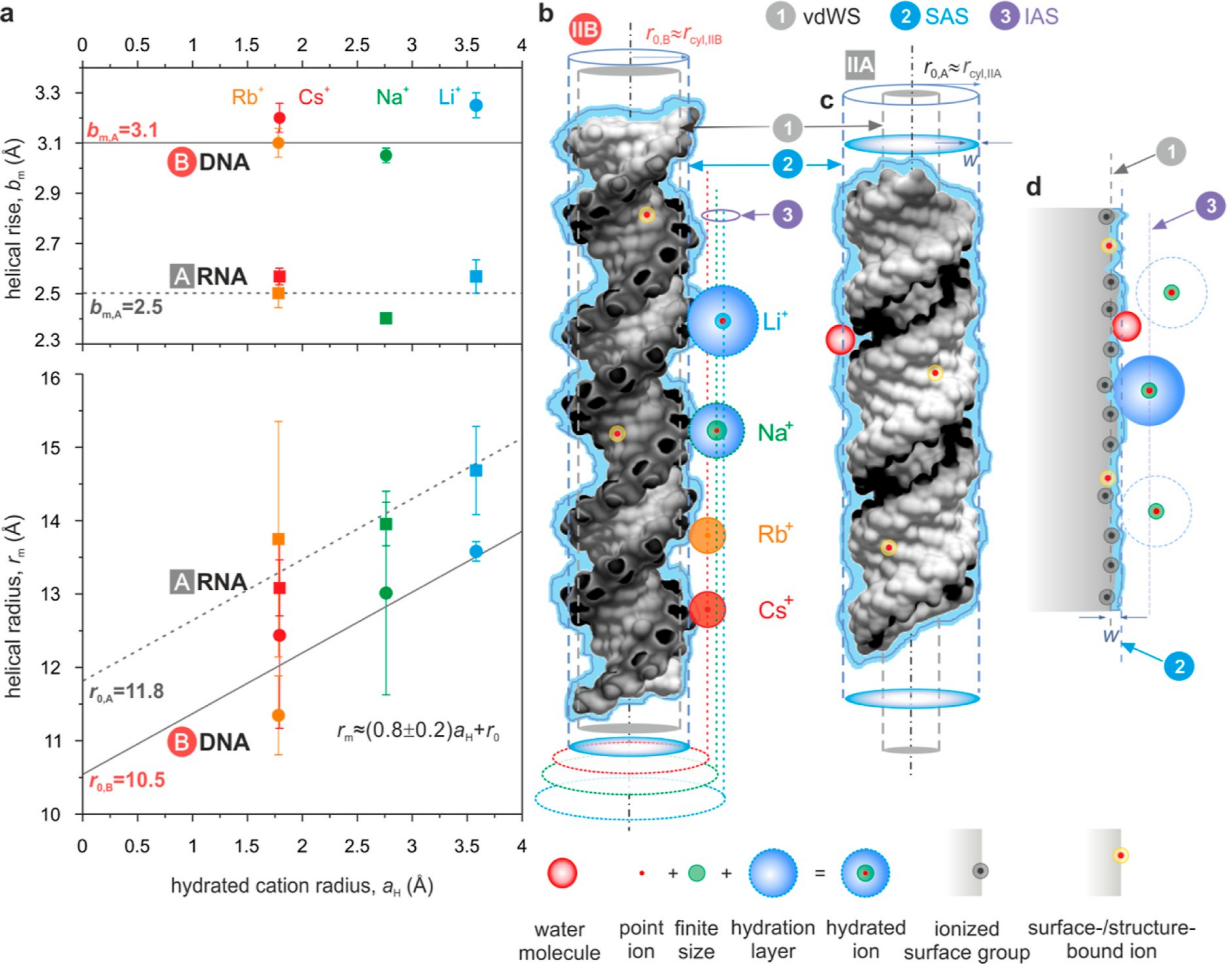
Inferring the structure of the molecule–electrolyte interface. (a) Measured helical rise per basepair, *b*_m_ (top), and radius, *r*_m_ (bottom), as a function of the hydrated cation radius, *a*_H_. Error bars denote s.e.m. Rise per basepair values show no significant variation with *a*_H_ and yield average values of  3.1 ± 0.1 Å and  2.5 ± 0.1 Å. Helical radius data were fit with a function of the form , yielding 10.5 ± 0.6 Å and  11.8 ± 0.6 Å. The slope, *k* = 0.8 ± 0.2, is a shared fit parameter in both relationships. (b) Cylinder of radius  10.5  (blue dashed cylinder) depicting that the effective cylinder in model-IIB of B-DNA is superimposed for comparison on the vdW molecular surface in model-IB (gray dashed cylinder). *k* = 0.8 ± 0.2 suggests that the distance of the closest approach of screening cations to the molecular surface is directly related to the radius of the hydrated cation species, *a*_H_. The resulting effective “ion accessible surface” (IAS) is the distance from the molecular axis beyond which the point-ion description of the electrolyte may be invoked (red, green, and blue dotted lines). The molecular structure may carry bound ions (yellow spheres) whose charge is included in . (c) For A-RNA, model-IIA which includes a SAS of thickness *w* = 3 Å meets the condition  12 Å (blue dashed cylinder). (d) Extrapolating the inferred structure of the molecule–electrolyte interface in (b) to a view of a macroscopic interface in solution where *w* < 3 Å.

We measured the radius, *r*, and axial rise per basepair, *b*, for dsDNA and dsRNA in solution containing alkali metal chlorides LiCl, NaCl, RbCl, and CsCl. Although the bare cationic radius decreases in the order Cs → Li, in an electrolyte, hydrated ionic radii increase with decreasing ionic radius due to favorable interactions between the ionic core and the surrounding polarizable water molecules (Figure 4). We found that our measured rise per basepair values for B-DNA and A-RNA are essentially insensitive to the nature of the cation in solution, and we obtained rise values averaged over all measurements of  3.1 ± 0.1 Å and  2.5 ± 0.1 Å for B-form and A-form helices, respectively (Figure 4a, top). These measurements compare well with values from crystallography and NMR.^2,3,5,45,49^

In contrast to the response of the helical rise to the cationic species in solution, we found that the inferred helical radii tended to increase in the order Cs → Li (Figure 4a, bottom). We further systematically found that  with an average difference in helical radii between A and B forms of about 1–2 Å. Using values for hydrated ionic radii, *a*_H_, determined from ionic mobilities and slip hydrodynamic boundary conditions, and plotting measured helical radii, *r*_m_, against *a*_H_, revealed a linear relationship between the two quantities.^50^ Extrapolating the measured *r*_m_ values to *a*_H_ = 0 yielded values for *r*_0_ that may be thought to represent the measured radii of equivalent cylinders in a hypothetical electrolyte containing point ions (Figure 4a). We obtained  10.5 ± 0.6 Å and  11.8 ± 0.6 Å for B-DNA and A-RNA, respectively (Figure 4a). Atomic models of B-DNA and A-RNA display axial radii of gyration of ≈6.7 and ≈7.8 Å and have helical radii of ≈8 and ≈9.5 Å based on the main backbone carbon atoms, respectively (Supporting Information Figure S10a). Thus, in addition to the average axial charge separation, our measurement is sensitive to the radial arrangement of atoms in the double helix. The latter appears to contribute to an effective electrical molecular surface topography, the geometry of which can be sensed even by a probe in the electrostatic far field, according to our measurements (Figure 2a and Supporting Information Figure S10b).^10^Figure S9 further examines the influence of various literature estimates of hydrated cationic radii on the inferred trends in *r*_m_.

Our measured *r*_0_ values may be thought to reflect a hypothetical experimental scenario involving point ions in solution (Supporting Information Section S7.5). We therefore expect these values to be amenable to direct comparison with the quantity  computed for the molecular models. We find that  10.5 ± 0.6 Å is comparable to  10.8 Å obtained for model-II of B-DNA that incorporates a SAS region of width *w* = 1.4 Å (Figures 4b and 2a). For A-RNA, we obtain agreement between the measured value of  11.8 ± 0.6 Å and a molecular model constructed using *w* = 3 Å, yielding  11.7 Å, as reflected in model II-A. Taken together, the measurements and the molecular electrostatic models for both B-DNA and A-RNA would point to the presence of a hydration layer of thickness 1 ≲ *w* ≲ 3 Å. This is in general agreement with the value of 1.4 ± 0.6 Å reported in a study using XPS of the Stern layer at the silica–water interface.^16,51^ Furthermore, the large disparity between the measured *r*_m_ value for A-RNA and the  value calculated for model-IA would appear to strongly preclude a molecular electrostatic model that neglects hydration at the molecular interface. A combination of the “hollow spine” along the A-RNA molecular axis, the deep and narrow major groove, and the closer packing of charged atoms in general would appear to render a measurement of the electrostatic free energy of A-RNA a more sensitive probe of interfacial structural detail and the finite size of ions in solution compared to B-DNA.^52,53^ Finally, our inferred slope for the *r*_m_ versus *a*_H_ relationship, *k* = 0.8 ± 0.2 ≈ 1, suggests that the radius of the effective cylindrical molecular surface contour in solution is enlarged by an amount that correlates with the radius of the hydrated cation (Figure 4c). Thus, in our picture, the thickness of the “Stern layer” at the molecular interface has a strong contribution from the size of the counterion in the electrolyte (Figure 4c,d).

Importantly, we find that a PB model of the electrostatics in conjunction with a geometric modification of the object—a slight inflation of the cylindrical radius in this case—is sufficient to model measured free energies in an experimental system with finite-sized ions.^42^ A comparison between all-atom molecular dynamics (MD) simulations and a PB model of nucleic acids reveals that the latter is capable of capturing many features evident in MD simulations, for example, integrated spatial free energy density profiles which are central to our work. However, it has also been pointed out that detailed agreement between a PB model and MD simulations, for example, at the level of spatial ionic densities in the major groove of A-RNA, will likely require a suitably modified PB theory.^53,54^ In future, a modified PB model for a charged cylinder of a fixed radius, which self-consistently accounts for hydration and finite ion-size effects, is likely to provide a common underlying framework to explain the results for both A- and B-form helices.^55^ Such a model will likely furnish more refined estimates of the interfacial parameters of interest, for example, *w*, *k*, and *a*_H_.

To conclude the study, we focus on *f*_M_, a parameter describing the experimental apparatus, determined in the measurement alongside *b*_m_ and *r*_m_. Like *r*_m_, we found that *f*_M_ displayed a systematic dependence on the cationic species in solution. For measurements that hold fixed all other experimental parameters, such as the slit height and salt concentration, any cation species-dependent variation in *f*_M_ = *f*_ion_^R^*f*_ϕ_ is expected to stem from either, or both, of the two interfacial sources: (1) cation-specific surface potential dependence of the silica surfaces, reflected in *f*_ϕ_ and/or (2) non-electrostatic cation interactions with the double helix captured by a relative ion binding affinity factor, given by *f*_ion_^R^. Our measured *f*_M_ values for various cationic species relative to those for the Na^+^ ion yielded on average  1.1,  0.9, and  0.9 (where ), and the affinity factors lie in the order Li > Na > Rb ≈ Cs (Supporting Information Figure S8). These values prove to be close to the “Hofmeister series”-dependent zeta (ζ) potentials reported for silica surfaces in alkali metal chloride solutions of concentration 10^–3^ M to 1 M, where  1.1 ± 0.1 and  0.8 ± 0.1 (Supporting Information Figure S8b).^56,57^ Assuming that the reported trend for the ζ-potential reflects the behavior of the effective surface electrical potential, ϕ_s_, in our experiments, our measured trends for *f*_M_ would suggest that most of the observed ion-dependent variation in *q*_m_ stems from the variation of surface potential of silica, captured by *f*_ϕ_. Our estimate of 0.9 ≲ *f*_Li_^R^ ≲ 1 would therefore point to a 10% reduction in , at the most, due to binding of Li^+^ cations to the molecule, that is,  0.1. Therefore, at present, we do not obtain evidence of relative cation affinity values, *f*_ion_^R^, that depart substantially from 1. To compare these observations with other techniques, Na^23^ NMR reports little significant sodium binding to DNA, with dissociation constants on the order of several molar.^58^ MD studies find that while monovalent cations do reside in the major and minor grooves of DNA, there is little preferential long-lived binding of monovalent cations (e.g., Li^+^ compared to Na^+^).^59^ However, AES reports weak affinities for Li^+^ cations corresponding to an amount of bound charge of ≈5–10% of , and transport measurements report decreased electrical mobility of DNA in the presence of Li^+^ cations.^14,60,61^ Our observation of an absence of substantial variation in relative affinity of alkali metal cations, and a possible weak affinity of Li^+^ for the double helix, is thus in broad agreement with previous observations.

## Discussion

It is important to note that although it may in principle be possible to evaluate electrostatic interaction free energies, , using molecular simulations such as Monte Carlo (MC) or MD methods,^53,62,63^ these techniques are computationally resource-intensive as the system size increases. Statistical simulation approaches such as MC require an exhaustive sampling of the configuration space in order to provide reliable results with acceptable accuracy.^64^ On the other hand, PB theory ignores correlations between ions but is nonetheless expected to provide satisfactory theoretical description of experiments involving monovalent salts in solution which is typical for ET*e* measurements. The PB approximation relies on the basic assumption that the potential of mean force for each ion type is equivalent to the mean electrostatic potential.^42,65^ This assumption neglects all higher-order ion correlations which manifest both through a long-range coulombic interaction and a short-range volume-excluded effect.^42^ These correlations are particularly important at high concentrations and in the presence of multivalent ions in solution.^66−68^ Nevertheless, comparison of PB ion densities with MC simulations involving finite-sized ions^69,70^ reveals unexpectedly good agreement, despite the fact that PB theory is typically thought of as a “point-ion” description of the problem.^42^ Although the reason for this behavior has not been fully understood, it might be attributed to fortuitous error cancellation within the PB approximation.^42^

Furthermore, in recent years, several attempts have been made to incorporate missing additional physics into the standard PB model by introducing different forms of modified PB equation.^68,71−73^ Although these modified PB models provide results that compare well with MC/MD simulations, their application to situations involving monovalent ions and dilute solutions does not lead to results which are significantly different from those of standard PB theory. Thus, in general, the PB approximation gives a satisfactory description for long-range electrostatic interactions of DNA molecules in monovalent electrolytes, which is also in agreement with MC simulations and hypernetted chain approximations.^67^ The fact that our measurements of the rise per basepair and radius of two classes of the double helix are so close to values known from high-resolution structural biology techniques, such as X-ray crystallography and NMR, may be viewed as evidence of the validity and applicability of the combined experimental and modeling approach described here.

In conclusion, although molecular simulations are gaining dramatically in sophistication and power, field theoretical descriptions of these systems remain important due to the high computational cost of problems involving explicit atoms in a many-body problem. We demonstrate that precise measurements of interaction free energies readily distinguish between structurally or conformationally distinct states of a molecular species. Viewed through the lens of the standing theoretical model for electrostatics, such measurements also provide information on molecular and interfacial structure. Although the approach does not furnish single-atom locations, it is capable of delivering more coarse-grained molecular structural information at high resolution, which could prove useful in analyzing molecular species that are challenging to crystallize or to isotope-label for NMR. Our findings further provide estimates of geometric parameters that describe the far-field properties and interactions of a polyelectrolyte in solution, for example, the effective molecular radius, the SAS, and the radii of ions at an interface. With the surface electrical characteristics of the system (given by *f*_M_) determined with high accuracy, we expect that in future, molecular electrometry measurements will be capable of yielding similar information on a molecular species using fewer independent measurements. For example, it may be possible to use our approach to directly measure sequence-dependent differences in the rise per basepair between different oligonucleotide species.^74^ Furthermore, given the sensitivity of the method to small differences in 3D conformation, for example, in helical geometry as shown in this work, it is likely that molecular electrometry will provide sensitive detection of more complex 3D conformational states and structural features such as loops and bubbles in molecules. Besides, the method is not limited to the study of rod-like molecules but can be readily extended to longer nucleic acids, as long as the measurements are then compared with free energies calculated for relevant molecular structural models.^53^ Although the present work relies on optical observation of about 1 zmol of a species, label-free optical detection could foster such measurements at the level of one molecule in solution, enabling analysis of biomolecular conformational or structural heterogeneity at the highest sensitivity.^75^ Finally, since ions and water tend to be disordered, they generally evade detection by high-resolution structural methods. Thus, beyond the structural properties of the molecule, our study furnishes a parameter-free, atomic-level view of the contact region between a molecule and the electrolyte phase (Figure 4b,c), reporting directly on the structure of the “Stern layer” at the liquid–solid interface in solution (Figure 4d).

## Materials and Methods

### ET*e* Experimental Procedure: The Measurement of Molecular Escape Time, *t*_esc_

Devices for ET*e* measurements were fabricated using silicon/silicon dioxide and glass substrates as previously described.^18^ Nanofabricated fluidic slits and nanostructured pocket regions were extensively characterized by scanning electron microscopy (SEM), AFM, and profilometry. We used nanoslits of height 2*h* = 71–77 nm and a width of about 5 μm and pockets of depth *d* = 140–160 nm and radii of either 250 or 300 nm. Nanoslits were loaded with a suspension of the molecular species of interest at a concentration of 50–70 pM using pressure-driven flow for about 1 min. The flow was then stopped, and the inlet and outlet reservoirs were filled with the same suspension and sealed to prevent evaporation. The system was allowed to equilibrate for 5–10 min and maintained in an argon atmosphere during the whole measurement.

The salt concentration in the electrolyte was monitored before and after the measurement by measuring solution conductivity with a microconductivity meter (Laquatwin, Horiba Scientific, Japan). The conductivity meter was calibrated for each salt species: LiCl, NaCl, RbCl, and CsCl (Supporting Information Figure S2d). Solution pH was measured before and after the measurement using a micro-pH electrode (InLab, Mettler Toledo, UK) and pH meter (Orion Star A215, Thermo Scientific, UK).

Optical measurements were performed using wide-field fluorescence imaging. Fluorescence excitation was achieved by illuminating the labeled molecules with a 532 nm DPSS laser (MGL_III-532_100 mW, PhotonTec, Berlin) that was focused at the back aperture of a 60×, NA = 1.35 oil immersion objective (Olympus, UK). Images were acquired using an sCMOS camera (Prime95B, Photometrics). Time-lapse videos were recorded using an exposure time  5 ms and a variable lag time between exposures, . The sampling frequency is the inverse of , where  =  +  is a factor 2–4 smaller than the average escape time, , for the molecular species of interest. Typical cycle times were in the range of 40–65 ms for 60 bp DNA/RNA, 25–40 ms for 40 bp DNA/RNA, and 15–25 ms for 30 bp DNA/RNA. Therefore, typical imaging frequencies were around 15–25 Hz for 60 bp DNA/RNA, 25–40 Hz for 40 bp DNA/RNA, and 40–67 Hz for 30 bp DNA/RNA.

Fluorescence images of molecular trapping were analyzed as described previously.^17^ Briefly, regions of interest (ROIs) centered on the locations of the individual traps were identified in an automated fashion. Intensity time traces for ROIs were analyzed using threshold intensity values to identify durations of trapping events, and the extracted residence times were pooled to construct escape time histograms (Supporting Information Figure S1a). Operating in the rapid escape regime, corresponding to average molecular residence times of Δ*t* ≈20–350 ms, we were able to acquire ≈10^4^ escape events within a total imaging time of 10–20 min for each molecular species of interest. Fitting the probability density of Δ*t* values with an exponential function of the form  yields the value of average escape time, , in any given measurement with an uncertainty of ≈1% (Supporting Information Figure S1a).

### Purification and Characterization of DNA and RNA Samples

All nucleic acid fragments were purchased from IBA Lifesciences (Germany) with a single ATTO 532 dye molecule coupled to either one 5′ end or both 5′ termini (Supporting Information Figure S2a). The oligomers were purified with reversed-phase high-performance liquid chromatography using a Reprosil-Pur 200 C18 AQ column (Dr. Maisch, Germany) and elution with a gradient of acetonitrile in an aqueous 0.1 M triethylammonium acetate solution at a flow rate of 5 mL/min. The integrity of DNA and RNA fragments was examined with 20% polyacrylamide native gel electrophoresis (Supporting Information Figure S2c), and the helical structures (A-form for dsRNA and B-form for dsDNA) were confirmed by acquiring circular dichroism (CD) spectra using a CD spectrometer (Chirascan, Applied Photophysics, UK). Nucleic acid samples in CD spectrometry measurements contained 1 mM NaCl and 1–1.3 mM Tris, similar to the electrometry measurements. CD spectra with a data resolution of 0.5 nm per point were recorded three times for each fragment and averaged (Supporting Information Figure S2b).

## Supporting Information Available

Considerations influencing the choice of NA fragments for the study; Converting measured escape times, t_esc_, to measured molecular effective charge, q_m_; Accounting for experimental inaccuracies; Inferring values of r, b and f_M_ from measurements of effective charge; Dependence of the inferred r_m_ vs. a_H_ relationship on the assumed values of a_H_; Comparing the inferred radii of two forms of the double helix; Electrostatic modeling and free energy calculations for B-DNA and A-RNA (PDF)
