# Supplementary material for: Opto-Electrostatic Determination of Nucleic Acid Double-Helix Dimensions and the Structure of the Molecule–Solvent Interface
Source: Macromolecules. 2022 Jul 1;55(14):6200–10. doi: 10.1021/acs.macromol.2c00657 (PMC9330769; doi:10.1021/acs.macromol.2c00657)
Supplement: Supplementary file 1 — ma2c00657_si_001.pdf [file ma2c00657_si_001.pdf]

Supplementary Information for

Opto-electrostatic determination of nucleic acid double helix dimensions and the structure of the molecule-solvent interface

*Maria Bespalova, Ali Behjatian, Narain Karedla, Rowan Walker-Gibbons & Madhavi Krishnan**

Physical and Theoretical Chemistry Laboratory, Department of Chemistry, University of Oxford, South Parks Road, Oxford OX1 3QZ, United Kingdom

Table of Contents

Supplementary Methods....2
Supplementary Discussion....3
S1. Considerations influencing the choice of NA fragments for the study....3
S2. Converting measured escape times, t_{esc}, to measured molecular effective charge, q_{m}....3
S3. Accounting for experimental inaccuracies....6
S4. Inferring the values of r, b and f_{M} from measurements of effective charge....11
S5. Dependence of the inferred r_{m} vs. a_{H} relationship on the assumed values of a_{H}....16
S6. Comparing the radii of two forms of the double helix....18
S7. Electrostatic modeling and free energy calculations for B-DNA and A-RNA....18
List of important variables and parameters....24
Fig. S1. Converting t_{esc} to q_{m}....25
Fig. S2. Characterization of B-DNA and A-RNA samples....26
Fig. S3. Measuring the hydrodynamic uncertainty, f_{H}....28
Fig. S4. Principle behind the measurement of b and r....29
Fig. S5. Influence of small perturbations of q_{dye} on r_{m} and b_{m} for $\ln f_{\mathrm{H}} = 0.007$....31
Fig. S6. Influence of small perturbations of q_{dye} on r_{m} and b_{m} for $\ln f_{\mathrm{H}} = 0.013$....33
Fig. S7. Example measured b-r probability manifolds for B-DNA and A-RNA....35
Fig. S8. The influence of ionic species on the value of f_{M}....36
Fig. S9. Dependence of the inferred r_{m} vs. a_{H} relationship on the assumed values a_{H}....37
Fig. S10. Electrostatic modeling for B-DNA and A-RNA....38
Table S1. Experimental details on all r and b measurements....40
References....42

Supplementary Methods

Fluorescence correlation spectroscopy (FCS) to determine molecular hydrodynamic radii, r_{H}

An important part of our data analysis involves Brownian Dynamics simulations which in turn require molecular hydrodynamic radii, r_{H}, values as inputs (described briefly in Sections 2 and 2.1 and extensively in previous work)[1, 2]. Molecular hydrodynamic radii were measured using single-focus FCS under solution conditions identical to the ETe measurements (1.2 mM NaCl, 1 mM Tris pH 9), with the addition of 0.01% Tween-20. FCS measurements were performed using a MicroTime 200 confocal single-molecule instrument (PicoQuant, Berlin, Germany). A 532 nm continuous-wave laser (LaserBoxx LBX-532-50-COL-PP; Oxxius) provided fluorescence excitation. Fluorescence emission was separated from the excitation light using a triple-band mirror (zt405/530/630rpc; Chroma, Irvine, CA), collected through a 100 μm pinhole, split according to polarization, and directed onto two detectors following filtering through a long-pass filter (532 LP Edge Basic, Chroma).

The fluorescence intensity cross-correlation for the two detectors was fit with an FCS correlation function, $G(\tau)$, that takes into account translational diffusion and triplet blinking in order to determine the diffusion time of a molecular species of interest, τ_{D}, through the confocal volume:

$$G(\tau) = 1 + \frac{1}{N}\left(1 + \frac{\tau}{\tau_{\mathrm{D}}}\right)^{-1}\left(1 + s^2\frac{\tau}{\tau_{\mathrm{D}}}\right)^{-1/2}\left(1 + c_{\mathrm{T}}\exp\left[-\frac{\tau}{\tau_{\mathrm{T}}}\right]\right) \quad \text{(S1)}$$

Here N is the average number of labeled molecules in the confocal volume, $s = 1/6$ denotes the ratio of the lateral to the axial radii of the confocal volume, τ_{T} is the average lifetime of a triplet state, and c_{T} is the fraction of labeled molecules that are in the triplet state in the confocal volume.

In order to estimate the value for τ_{T}, we first performed FCS measurements on the intrinsically disordered activator for thyroid hormone and retinoid receptors ACTR labeled with ATTO 532 dye, as well as on free ATTO 532 dye molecules[3]. The value for r_{H} for ACTR was obtained from the literature and is equal to 2.3 nm[4]. The value for r_{H} for ATTO 532 dye was estimated from an independent Dual-focus Fluorescence Correlation Spectroscopy (2fFCS) measurement, as described in our previous work, and is equal to 0.61 nm[1]. Using the value for $\tau_{\mathrm{T}} = 6$ μs, we obtained $\tau_{\mathrm{D,ACTR}}/\tau_{\mathrm{D,ATTO\ 532}} = 3.78$ which agrees well with the expected ratio of radii, $r_{\mathrm{H,ACTR}}/r_{\mathrm{H,ATTO\ 532}} = 3.77$. Setting $\tau_{\mathrm{T}} = 6$ μs we fit the measured correlation data to equation (S1) in order to determine a value for τ_{D} for each nucleic acid species. In order to relate these diffusion times through the optical focus to molecular radii we use a value for the hydrodynamic radius of 60 dsDNA, $r_{\mathrm{H,60dsDNA}} = 4.55$ nm, previously measured using two-focus FCS[1]. Specifically, we use the relation $r_{\mathrm{H},i} = \tau_{\mathrm{D},i} \cdot r_{\mathrm{H,60dsDNA}}/\tau_{\mathrm{D,60dsDNA}}$ in order to determine the unknown $r_{\mathrm{H},i}$ value of the test species from the measured $\tau_{\mathrm{D},i}$ value. Further, we performed linear fits of the data for r_{H} vs n, the number of basepairs, for B-DNA and A-RNA (Fig. S3a, solid lines). For the Brownian Dynamics (BD) simulations, described in section S2.1, we used the fit values for r_{H} for each molecular species, listed in the table in Fig. S3a.

We also fitted the measured r_{H} vs. n data with an equation describing the diffusion of cylinders in solution: $r_{\mathrm{H}} = l\ /\ 2[\ln(\ l/2r) + \nu]$. Here $l = bn$ denotes the contour length of the molecule, b is the axial rise per basepair, r is the radius of the cylinder which may be regarded as comparable with the radius of nucleic acid double helix, and υ is a correction factor accounting for end effects. The equation above combines the equation for the diffusion coefficient of cylinders of finite length modeled as stacks of rings, $D = k_{\mathrm{B}}T[\ln(\ l/2r) + \nu]/3\pi\mu l$, with the Stokes-Einstein equation, $D = k_{\mathrm{B}}T/6\pi\mu r_{\mathrm{H}}$[5]. Equating the above two expressions effectively maps the diffusive behavior of a cylinder in solution on to the motion of a sphere of radius, r_{H}. Here, D is the nucleic acid molecule's diffusion coefficient, μ is the solvent viscosity and $k_{\mathrm{B}}T$ is the product of the Boltzmann constant, k_{B}, and the temperature, T. Performing the fitting procedure with three fit parameters, namely b, r and

υ, we obtained the following values: $b = 4.4 \pm 8.5$ Å, $r = 9.3 \pm 18516$ Å, $\upsilon = 0.26 \pm 2001$ for B-DNA, and $b = 2.6 \pm 1.9$ Å, $r = 10.4 \pm 4042$ Å, $\upsilon = 0.06 \pm 389$ for A-RNA (Fig. S3a, dashed lines). Interestingly the fit value for b for A-RNA agrees well with the nominal value from crystallography, $b = 2.6$ Å. We then fixed b values for both helical forms to the nominal crystallographic values of 3.4 Å for B-DNA and 2.6 Å for A-RNA and repeated the fitting procedure with two fit parameters, r and υ. We obtained the following values: $r = 9.8 \pm 1255$ Å, $\upsilon = -0.004 \pm 127$ for B-DNA, and $r = 9.7 \pm 315$ Å, $\upsilon = -0.02 \pm 32$ for A-RNA (Fig. S3a, dotted lines). The nominal mean values for r are close to $\approx$10 Å, typical for nucleic acid double helices, but the uncertainties are very large due to the weak, logarithmic dependence of the measured D values on r.

Supplementary Discussion

S1. Considerations influencing the choice of nucleic acid fragments for the study

Our experimental study requires precise measurement of effective charge for at least three short nucleic acid fragments under identical experimental conditions. Identical conditions refer to the same measurement device and buffer conditions with effectively identical pH and salt concentration, c, within experimental measurement error.

Based on previous experience, we chose to perform measurements on double stranded nucleic acid fragments characterized by a value of $q_{\mathrm{eff}} \approx -40\ e$, corresponding to a 60 bp dsDNA molecule[1]. Fragments that are very similar in length entail effectively redundant $Q_n(b, r)$ functions (see Section S4). Given the experimental error on measurement values, the similarity in the resulting functions would therefore not be conducive to extracting unique solutions for the unknown quantities of interest (b and r). In addition, for this study we required that all fragments satisfied the rigid rod model of DNA. Odijk-Skolnick-Fixman theory predicts that the overall persistence length, l_{p}, of a semi-flexible polymer increases with decreasing salt concentration[6]. In particular, the electrostatic contribution, $l_{\mathrm{OSF}} = l_{\mathrm{B}}/4L^2\kappa^2$, to the intrinsic persistence length increases in proportion to salt concentration, c, for a monovalent salt solution. Here l_{B} denotes the Bjerrum length and $L = b/2$ represents the linear charge spacing on the polymer, where b is the rise per basepair in this work. Further, κ^{-1} denotes the Debye length which for a monovalent electrolyte is given by $\sqrt{\varepsilon\varepsilon_0 k_{\mathrm{B}}T/2cN_{\mathrm{A}}e^2}$. Here, $\varepsilon\varepsilon_0$ is the static permittivity of the medium, N_{A} is Avogadro's number, and e is the elementary charge. Given that the polymer persistence lengths are 150 bp for dsDNA and about 430 bp for dsRNA in high salt concentration solutions ($\approx$100 mM), our chosen fragment lengths of 30 bp, 40 bp, and 60 bp are expected to lie well within the required rigid rod limit for salt concentrations, $c \approx$1.2 mM, used in this study, while still providing non redundant $Q_n(b, r)$ relations conducive to the study[7].

The sequences of the dsDNA and dsRNA fragments in our study are shown in Fig. S2a. Sequences were designed such that the individual strands are expected to form no stable hairpin structures by internally complementary base-pairing. We therefore expect stoichiometric mixtures of the strands to form the expected stable double stranded structures upon annealing in solution, with the values for Gibbs free energy of the duplex formation in the range of $\approx$100-200 $k_{\mathrm{B}}T$ per duplex formed.

S2. Converting measured escape times, t_{esc}, to measured molecular effective charge, q_{m}

In general, the depth of the potential well , W, links t_{esc} with the molecule's effective electrical charge, q_{eff}. Simple physical insight into the relationship between t_{esc} and W is given by Kramers relation[8]:

$$t_{\mathrm{esc}} = t_{\mathrm{r}} \exp\left(\frac{W}{k_{\mathrm{B}}T}\right) \quad \text{(S2)}$$

which holds in the regime of $W > 4\ k_{\mathrm{B}}T$, and shows that the experimental measurable t_{esc} depends exponentially on W. Here, the position relaxation time of the molecule, t_{r}, is treated as a fit parameter and depends on geometric features of the potential well and is also inversely proportional to the molecule's diffusion coefficient, $D = k_{\mathrm{B}}T/6\pi\mu r_{\mathrm{H}}$ in the trap, where r_{H} denotes the hydrodynamic radius of the object and μ is the solvent viscosity.

In order to convert measured escape time, t_{esc}, to q_{eff} we first convert t_{esc} to W. W is next converted to the electrostatic free energy difference ΔF_{el} and we have also $\Delta F_{\mathrm{el}} \propto q_{\mathrm{eff}} \propto q_{\mathrm{m}}$. Here q_{m} represents a measured effective charge which may not be identical to the theoretical quantity q_{eff} due to the presence of measurement uncertainties, discussed in section S3. The process of inferring values of q_{m} from measurements of t_{esc} is described below.

S2.1 Converting measured escape time, t_{esc}, to the total well depth, W

In order to convert the average measured escape time, t_{esc}, to the depth of the potential well W, we performed Brownian Dynamics (BD) simulations of the escape process in a lattice of traps of identical geometry, reflecting the experimental situation as described in our previous work[2]. Simulations were performed for a range of input values for trap depth, W (Fig. S1b).

For various values of well depth, W, we simulated trajectories of single molecule motion through a lattice of traps. We then used these trajectories to generate stacks of optical images including all attributes of the imaging process and system, such as the optical point spread function, exposure time, lag time, average signal and background intensities, background noise, dark noise, shot noise, and laser fluctuations (Fig. S1d and e). These simulated movies corresponding to known input values of W were analysed in a manner identical to the experimental data, using the same intensity thresholds on the signal to extract average simulated escape times, $t_{\mathrm{esc,s}}$. A plot of W vs $t_{\mathrm{esc,s}}$ values was fit with a second degree polynomial and used to convert the experimentally measured t_{esc} value to the total well depth, W (Fig. S1b).

S2.2 Converting the inferred well depth, W, to q_{eff}

In our previous work, we have shown that the total well depth, W, can be written as the difference between the values of the system free energy when the molecule is outside (slit region, denoted by state 1) and inside the trap (pocket region, state 2)[9, 10]. The total free energy includes the electrostatic free energy of interaction, ΔF_{el}, and an entropic term, f, arising from spatial fluctuation of the molecule. In this work we extend the previous description to include a free energy term due to rotational motion, ΔF_{rot}, of the molecule in the system as follows:

$$W = W|_1 - W|_2 = \Delta F_{\mathrm{el}} + f = q_{\mathrm{eff}}\phi_{\mathrm{m}} + \Delta F_{\mathrm{trans}} + \Delta F_{\mathrm{rot}} \quad \text{(S3)}$$

Here ϕ_{m} is the electrical potential at the midplane in the slit region. ϕ_{m} is an accurate estimate of the electrical potential difference, $\Delta\phi$, that gives rise to the trap, since the electrical potential in the "pocket" region is zero by design. A large contribution to W in our experiments arises from the first term, $\Delta F_{\mathrm{el}} = q_{\mathrm{eff}}\phi_{\mathrm{m}}$, which denotes the electrostatic contribution to the total free energy when a charged object moves from the electrostatic energy minimum at the midplane of the slit (state 1) to midplane location along the axis of cylindrical symmetry in the pocket region (state 2). Further, $\Delta F_{\mathrm{trans}}$ and ΔF_{rot} denote contributions to W that arise from two different types of spatial fluctuations. $\Delta F_{\mathrm{trans}}$ denotes the free energy contribution from position fluctuations of the center of mass of the molecule in the depth (z) dimension of the slit, while ΔF_{rot} denotes the free energy contribution from rotational motion of a rod-shaped molecule[10]. In each case $\Delta F_{\mathrm{trans}}$ and ΔF_{rot} denote differences in

the corresponding free energies between states "1" and "2". For the translational mode, the axial fluctuation free energy term may be calculated by first writing the local molecular partition function for each state of the system:

$$Z = \int_0^{z_{\max}} e^{\frac{-q_{\mathrm{eff}}\phi(z)}{k_{\mathrm{B}}T}} dz \tag{S4}$$

Here $\phi(\mathrm{z}) = \phi_{\mathrm{m}}\cosh\left[\kappa\left(z - \frac{z_{\max}}{2}\right)\right]$ is the electrical potential at an axial location, z, in the parallel plate gap and $q_{\mathrm{eff}}\phi(z)$ is the object's local electrical interaction energy. The limits of integration $z = 0$ and $z = z_{\max}$ denote the lower and upper wall of the parallel plate gap respectively, with $z_{\max} = 2h$ or $(2h + d)$ depending on whether the free energy calculated concerns the slit region or in the pocket region respectively. Here d is the depth of the pocket structure. The above equation holds for a point object which we have verified approximates well an object of finite size. This is because Boltzmann weighting to the midplane of the slit ensures that the molecule never approaches the walls close enough for finite size effects to make a substantial difference to the partition function. As shown previously, $\Delta F_{\mathrm{trans}}$, is then given by[10]:

$$\Delta F_{\mathrm{trans}} = -k_{\mathrm{B}}T \ln Z|_1 + k_{\mathrm{B}}T \ln Z|_2 - q_{\mathrm{eff}}\phi_{\mathrm{m}} \tag{S5}$$

Next, in order to model the angular motion of a rod-shaped molecule we write a partition function that describes rotational motion of a line charge of length, l, and charge density $\sigma_l = q_{\mathrm{eff}}/l$ located in the vicinity of the midplane of the slit. The angular potential energy landscape sampled by a rod oriented with its long axis at a polar angle θ to the z-axis of the slit (Fig. 1a) and with its midpoint located at a height, z, far away from the walls, is given by

$$\Delta F_{\mathrm{r}}(z,\theta) = \frac{1}{\cos\theta}\int_{z-\frac{l}{2}\cos\theta}^{z+\frac{l}{2}\cos\theta} \sigma_l \phi(z)\mathrm{d}z \tag{S6}$$

We may also write the above equation in simpler form: $\Delta F_{\mathrm{r}}(z,\theta) = q_{\mathrm{eff}}\langle\phi(z)\rangle$, where $\langle\phi(z)\rangle$ denotes the average electrical potential evaluated over a height in the slit ranging from $z - \frac{l}{2}\cos\theta$ to $z + \frac{l}{2}\cos\theta$. For example, for $\theta = 90°$, and $z = z_{\max}/2$, Eq. (S6) reduces to $\Delta F_{\mathrm{r}}(z,\theta) = q_{\mathrm{eff}}\phi_{\mathrm{m}}$, which is the electrostatic interaction energy of a rod of charge q_{eff} lying along the mid-plane of the slit. Since the problem is azimuthally symmetric around the z-axis of the slit, the angular partition function for a rod centered on and confined to the midplane of the parallel-plate gap at $z = z_{\max}/2$ is given by

$$\Omega = 4\pi \int_0^{\pi/2} \exp\left[-\Delta F_{\mathrm{r}}(z_{\max}/2\,,\theta)/k_{\mathrm{B}}T\right] \sin\theta\, \mathrm{d}\theta \tag{S7}$$

Similar to equation (S5) above, we write

$$\Delta F_{\mathrm{rot}} = -k_{\mathrm{B}}T \ln\Omega|_1 + k_{\mathrm{B}}T \ln\Omega|_2 - q_{\mathrm{eff}}\phi_{\mathrm{m}} \tag{S8}$$

Note that equation (S8) represents the rotational free energy difference for a rod in states confined to the midplane regions of the slit and pocket. We have verified that equation (S8) is in fact an accurate estimate of the total rotational component of the free energy arising from the full translational-rotational motion of a rod that exhibits both degrees of freedom simultaneously. Once again, this is

because probabilities of occupancy are Boltzmann weighted to the midplane of the slit causing the rotational contribution to the free energy to be dominated by dynamics at the midplane.

In general in these measurements we find that for a nominal well depth of $W = 5\ k_{\mathrm{B}}T$ the translation contribution to the free energy, $\Delta F_{\mathrm{trans}} \approx 2.3\ k_{\mathrm{B}}T$, while the rotational free energy, $\Delta F_{\mathrm{rot}} \approx 0.05\text{-}0.2\ k_{\mathrm{B}}T$, leaving $\Delta F_{\mathrm{el}} = q_{\mathrm{eff}}\phi_{\mathrm{m}} \approx 2.5\ k_{\mathrm{B}}T$ which is the experimental quantity of interest (Fig. S1c). We further point out that while ΔF_{el} depends linearly on q_{eff} and ϕ_{m}, $\Delta F_{\mathrm{trans}}$ and ΔF_{rot} depend weakly (logarithmically) on q_{eff} and ϕ_{s}, and are therefore relatively insensitive to both quantities[10]. Knowing the geometry of the slit and given a nominal value of the surface potential of the wall, ϕ_s, we are able to calculate a relationship between W and q_{eff}. Once W is known from the experimental measurement we obtain a value for the measured effective charge, q_{m}, by reading it off a W vs q_{eff} plot shown in Fig. S1c. This value of q_{m} for each fragment is used in subsequent analyses.

We point out that in this work the rotational term, ΔF_{rot}, was not uniformly applied in the data analysis. The need for the small rotational contribution to the free energy was only apparent in a few datasets that involved comparatively lower magnitudes of surface electrical potential ($\phi_s \approx -2\ k_{\mathrm{B}}T/e$), with these datasets yielding no measurement outcomes for b and r otherwise (Table S1). In all remaining datasets (a dataset consists of q_{eff} measurements on three nucleic acid fragments), inclusion of the rotational free energy contribution led to no measurement outcome for b and r. The origin of this variation is currently not clear, but we emphasize that measurement outcomes were obtained in a mutually exclusive fashion, i.e., the ΔF_{rot} term had to either be included or excluded in the data analysis in each case. Measurement outcomes for b and r were never obtained in both scenarios, which precluded a situation where a given dataset yielded two potentially disparate sets of results.

We further noted that the measurement of the rise per basepair, *b,* and radius, *r,* was very sensitive to minor perturbations (1-2 %) in q_{eff} on any single fragment. The overall observation in the data analysis was that datasets frequently returned no intersection plot, as shown in Fig. S4b, which resulted in no measurement outcomes for *b* and *r*. Overall about 30% of the datasets, which were considered free of perturbations attributable to external sources, yielded results for *b* and *r*, all of which are tabulated in Table S1. This implies a 70% probability of achieving a single measurement of q_{eff} with ≈99% accuracy, which is reasonably good.

S3. Accounting for experimental inaccuracies

As follows from the equation (S3), in an ideal experiment, without any measurement uncertainties, the electrical free energy of a molecule carrying charge q_{t} may be written as: $\Delta F_{\mathrm{el}} = \phi_{\mathrm{m}} \cdot q_{\mathrm{t}} = W - \Delta F_{\mathrm{trans}} - \Delta F_{\mathrm{rot}}$. As demonstrated in previous work, the main contribution to the measurement inaccuracy in F_{el} arises from the uncertainty on the midplane potential, ϕ_{m}[1]. The midplane potential, in turn, depends on the surface potential of the wall, ϕ , and the measured slit height, $2h$, where h includes small measurement uncertainties, $h_{\mathrm{e}} \approx 1\text{-}2$ nm, from AFM measurements, and is well approximated by

$$\phi_{\mathrm{m}} = 2\phi_{\mathrm{s}}\exp(-\kappa h) \tag{S9}$$

For the surface potential, we used a nominal working value of $\phi_{\mathrm{s}} = -2.8\ k_{\mathrm{B}}T/e$, estimated in previous measurements on a range of biomolecules in 1 mM NaCl, 1 mM Tris[9]. However, the true value of ϕ_{m} in any given experiment is subject to small variations on slit height (≈1 nm) and measurement error on salt concentration (<0.5%). Furthermore, a small variation in ϕ may also arise from ion-specific effects on ϕ_{s}[11]. To account for the uncertainties on both parameters, we introduced a new variable – the multiplicative correction factor, f_{M}. This factor corrects the nominal midplane potential, obtained using $\phi_{\mathrm{s}} = -2.8\ k_{\mathrm{B}}T/e$ and measured slit height, $2h$, as given by equation (S9) to the true value of midplane potential, $\phi_{\mathrm{m,t}}$:

$$\phi_{\mathrm{m,t}} = \phi_{\mathrm{m}} \cdot f_{\mathrm{M}} \tag{S10}$$

Note that f_{M} can also capture systematic departures of ϕ_{s} from the nominal value arising from ion specific effects.

The second source of error is encountered in extracting the well depth of the trap, W, using two experimental measurables: the molecule's escape time, t_{esc}, and its position relaxation time, t_{r}, as given by equation (S2). As described above, t_{r} is inversely proportional to the molecule's diffusion coefficient, D. The dominant contribution to the uncertainty in the diffusion coefficient originates from measurement uncertainties in FCS and any contribution to the altered hydrodynamic drag on the molecules arising from confinement in a parallel plate system. To correct for these uncertainties, we have introduced the hydrodynamic correction factor, f_{H}, which relates the measured hydrodynamic radius, $r_{\mathrm{H},}$ to its true value, $r_{\mathrm{H,t}}$ as follows:

$$r_{\mathrm{H}} = r_{\mathrm{H,t}} \cdot f_{\mathrm{H}} \tag{S11}$$

Therefore, the interpreted or measured well depth, W, can differ from the true well depth, W_{t}, as given by

$$W_{\mathrm{t}} = W + k_{\mathrm{B}}T\ln f_{\mathrm{H}} \tag{S12}$$

The above relationship (S11) has been verified using Brownian Dynamics simulations. Using the equations (S3), (S10) and (S12), we obtained the following expression for the measured effective electrical charge, q_{m}, in a real experiment with measurement uncertainties:

$$q_{\mathrm{m}} = \frac{W - \Delta F_{\mathrm{trans}} - \Delta F_{\mathrm{rot}}}{\phi_{\mathrm{m}}} = f_{\mathrm{M}}\left(q_{\mathrm{t}} - k_{\mathrm{B}}T\frac{\ln f_{\mathrm{H}}}{\phi_{\mathrm{m,t}}}\right) \tag{S13}$$

Note that here we implicitly use the relation $q_{\mathrm{t}} = (W_{\mathrm{t}} - \Delta F_{\mathrm{trans}} - \Delta F_{\mathrm{rot}})/\phi_{\mathrm{m,t}}$, where q_{t} is a quantity that is free of all known experimental inaccuracies (f_{M} and f_{H}). Furthermore, the nucleic acid molecules in our experiments are labeled with two ATTO 532 dyes, each carrying a net structural charge, $q_{\mathrm{str}} = -1e$. So the measured charge of a molecular species in an ideal experiment is expected to be

$$q_{\mathrm{t}} = q_{\mathrm{eff}} + 2q_{\mathrm{dye}}. \tag{S14}$$

Here, q_{eff} denotes the effective electrical charge of the nucleic acid double helix, and q_{dye} is the effective charge of a single dye attached to each 5' terminus of the molecule. Equation (S13) can thus be rewritten as:

$$q_{\mathrm{m}} = f_{\mathrm{M}}\left(q_{\mathrm{eff}} + 2q_{\mathrm{dye}} - k_{\mathrm{B}}T\frac{\ln f_{\mathrm{H}}}{\phi_{\mathrm{m,t}}}\right) \tag{S15}$$

Hydrodynamic uncertainties, as captured by a single factor f_{H}, and the effective charge of the dye, q_{dye}, thus both enter the molecule's measured effective charge, q_{m}, in an additive fashion. For convenience, they can be combined into a single additive correction factor, $f_{\mathrm{A}} = 2q_{\mathrm{dye}} - k_{\mathrm{B}}T\frac{\ln f_{\mathrm{H}}}{\phi_{\mathrm{m,t}}}$, so that we may write

$$q_{\mathrm{m}} = f_{\mathrm{M}}(q_{\mathrm{eff}} + f_{\mathrm{A}}) \tag{S16}$$

Importantly, both quantities in the additive correction factor – q_{dye} and f_{H} – can be estimated in independent measurements as described further in sections S3.1 and S3.2.

Once the value of f_{A} is known we obtain corrected values of the measured effective charge, q_{m}, for all nucleic acid fragment lengths. We then compared these values with the corresponding calculated effective charges, q_{eff}, in order to extract values for the three unknowns of interest: b, r and f_{M}, as described in section S4.

S3.1 Measuring the hydrodynamic uncertainty, f_{H}

Using equation (S10), equation (S13) can be rewritten as:

$$q_{\mathrm{m}} = f_{\mathrm{M}} \cdot q_{\mathrm{t}} - k_{\mathrm{B}}T\frac{\ln f_{\mathrm{H}}}{\phi_{\mathrm{m}}} \tag{S17}$$

For two measurements of q_{m} of the same nucleic acid species, measured in two slightly different salt concentrations, we may write the difference in measured charges as follows:

$$q_{\mathrm{m},1} - q_{\mathrm{m},2} = q_{\mathrm{t}}(f_{\mathrm{M},1} - f_{\mathrm{M},2}) + k_{\mathrm{B}}T\ln f_{\mathrm{H}}\left(\frac{1}{\phi_{\mathrm{m},2}} - \frac{1}{\phi_{\mathrm{m},1}}\right) \tag{S18}$$

Indices 1 and 2 in the subscripts denote the values for the respective parameters in the two measurements performed.

The first term on the RHS of equation (S18) can be simplified using the equations (S9) and (S10):

$$f_{\mathrm{M}} = \frac{\phi_{\mathrm{m,t}}}{\phi_{\mathrm{m}}} = \frac{2\phi_{\mathrm{s,t}}\exp[-\kappa(h + h_{\mathrm{e}})]}{2\phi_{\mathrm{s}}\exp(-\kappa h)} = \frac{\phi_{\mathrm{s,t}}}{\phi_{\mathrm{s}}}\exp(-\kappa h_{\mathrm{e}})$$

$$\frac{f_{\mathrm{M},2}}{f_{\mathrm{M},1}} = \frac{\exp(-\kappa_2 h_{\mathrm{e}})}{\exp(-\kappa_1 h_{\mathrm{e}})} = \exp[h_{\mathrm{e}}(\kappa_1 - \kappa_2)]$$

For example, for measurements performed in salt concentrations of 1.20 mM and 1.30 mM, $\kappa_1{}^{-1} =$ 8.8 nm and $\kappa_2{}^{-1}$ =8.4 nm, respectively. We further have
$|h_{\mathrm{e}}(\kappa_1 - \kappa_2)| = 1\ \mathrm{nm} \cdot |\ 0.114\ \mathrm{nm}^{-1} - 0.119\ \mathrm{nm}^{-1}\ | = 0.005 \ll 1$. Thus, we can use the following Taylor's series approximation:

$$\frac{f_{\mathrm{M},2}}{f_{\mathrm{M},1}} = \exp[h_{\mathrm{e}}(\kappa_1 - \kappa_2)] \approx 1 + h_{\mathrm{e}}(\kappa_1 - \kappa_2)$$

which gives $f_{\mathrm{M},2} \approx f_{\mathrm{M},1}[1 + h_{\mathrm{e}}(\kappa_1 - \kappa_2)]$

The first term on the RHS of equation (S18) is therefore

$$q_{\mathrm{t}}(f_{\mathrm{M},1} - f_{\mathrm{M},2}) = -q_{\mathrm{t}}f_{\mathrm{M},1}h_{\mathrm{e}}(\kappa_1 - \kappa_2)$$

We further can simplify the second term on the RHS of equation (S18):

$$k_B T \ln f_H \left(\frac{1}{\phi_{m,2}} - \frac{1}{\phi_{m,1}}\right) = k_B T \ln f_H \left(\frac{1-\phi_{m,2}/\phi_{m,1}}{\phi_{m,2}}\right)$$

where $\frac{\phi_{m,2}}{\phi_{m,1}} = \frac{2\phi_s \exp(-\kappa_2 h)}{2\phi_s \exp(-\kappa_1 h)} = \exp\,[h(\kappa_1 - \kappa_2)]$

Here we have
$|h(\kappa_1 - \kappa_2)| = 36\text{nm} \cdot |\ 0.114\ \text{nm}^{-1} - 0.119\ \text{nm}^{-1}\ | = 0.18 < 1$. Thus, we use a few additional terms in the Taylor's series approximation

$$\frac{\phi_{m,2}}{\phi_{m,1}} = \exp[h(\kappa_1 - \kappa_2)] \approx 1 + h(\kappa_1 - \kappa_2) + \frac{h^2(\kappa_1 - \kappa_2)^2}{2} + \frac{h^3(\kappa_1 - \kappa_2)^3}{6}$$

We can now rewrite the second term on the RHS of equation (S18) as follows:

$$k_B T \ln f_H \left(\frac{1}{\phi_{m,2}} - \frac{1}{\phi_{m,1}}\right) = -\frac{k_B T \ln f_H}{\phi_{m,2}}\left[h(\kappa_1 - \kappa_2) + \frac{h^2(\kappa_1 - \kappa_2)^2}{2} + \frac{h^3(\kappa_1 - \kappa_2)^3}{6}\right]$$

Finally, equation (S18) can be rewritten as follows:

$$\frac{q_{m,1} - q_{m,2}}{\kappa_2 - \kappa_1} = q_t \cdot f_{M,1} \cdot h_e + \frac{k_B T \ln f_H}{\phi_{m,2}}\left[h + \frac{h^2(\kappa_1 - \kappa_2)}{2} + \frac{h^3(\kappa_1 - \kappa_2)^2}{6}\right] \quad \text{(S19)}$$

We emphasize that in an ideal experiment, without any slit height and hydrodynamic uncertainties, i.e., $h_e = 0$ and $f_H = 1$ respectively, Δq would be expected to be zero, as evident above. But in fact measurements show that the value of $q_{m,1} - q_{m,2}$ is in general around -0.5 e when $\kappa_2 - \kappa_1$ is around -0.007, suggesting the presence of inaccuracies in slit height and/or hydrodynamic quantities.

Equation (S19) has two unknown parameters, $f_{M,1} \cdot h_e$ and $\ln f_H$. The quantity on the LHS, $(q_{m,1} - q_{m,2})/(\kappa_2 - \kappa_1)$, is known from measurement. Furthermore on the RHS, values for h and $\phi_{m,2}$ are set equal to nominal values taken from AFM measurements and equation (S9), respectively. As the true effective charge of the labeled molecule, $q_t = q_{eff} + 2\ q_{dye}$, also remains unknown at this point, we assume as an estimate, the calculated value of effective charge, q_{eff}, which corresponds to crystallographic structural parameters for the helical radius, r, and rise per basepair, b, describing the form of the double helix used in the particular experiment. We further assume $q_{dye} \cong -0.5\ e$ (which is known from calculation). Because $\left|(q_t \cdot f_{M,1} \cdot h_e)\left[(\kappa_2 - \kappa_1)/(q_{m,1} - q_{m,2})\right]\right| \approx 0.4$, the precise value of q_t used could be expected to impact the estimate of f_H, but in fact a reasonable estimate for q_t within 10% of the true value would suffice. For example, a 5% alteration in the assumed value of q_t (which implies very different helical parameters compared to the nominal helical geometry assumed) alters the value of $\ln f_H$ by about 3% which is negligible for our purposes. In practice, in solving two equations of the form (S19), any systematic uncertainty factor multiplying q_t would be absorbed into the value of the first unknown ($f_{M,1} \cdot h_e$), leaving the unknown quantity of interest, $\ln f_H$, unaffected.

In order to determine f_H from equation (S19), we performed replicates of escape-time measurements on two nucleic acid fragment lengths, each measured in two different salt concentrations. This gives a system of two equations with two unknowns. For example, for 30 bp and 60 bp nucleic acid fragments, we obtained the following system of two equations:

$$\left.\frac{q_{\mathrm{m},1}-q_{\mathrm{m},2}}{\kappa_2-\kappa_1}\right|_{30} = q_{\mathrm{t},30}\cdot f_{\mathrm{M},1}\cdot h_{\mathrm{e}} + \frac{k_{\mathrm{B}}T\ln f_{\mathrm{H}}}{\phi_{\mathrm{m},2}}\left[h + \frac{h^2(\kappa_1-\kappa_2)}{2} + \frac{h^3(\kappa_1-\kappa_2)^2}{6}\right] \quad \text{(S19a)}$$

$$\left.\frac{q_{\mathrm{m},1}-q_{\mathrm{m},2}}{\kappa_3-\kappa_1}\right|_{60} = q_{\mathrm{t},60}\cdot f_{\mathrm{M},1}\cdot h_{\mathrm{e}} + \frac{k_{\mathrm{B}}T\ln f_{\mathrm{H}}}{\phi_{\mathrm{m},2}}\left[h + \frac{h^2(\kappa_1-\kappa_2)}{2} + \frac{h^3(\kappa_1-\kappa_2)^2}{6}\right] \quad \text{(S19b)}$$

Note that measurements for both molecular species were performed keeping at least one salt concentration the same for both species so that $f_{\mathrm{M},1}$ was identical. Due to the presence of measurement errors, we solved the equations (S19a) and (S19b) graphically as shown in Fig. S3b. For every measurement, we defined the solution space of possible $f_{\mathrm{M},1}\cdot h_{\mathrm{e}}$ and $\ln f_{\mathrm{H}}$ values, which satisfied the measured values for $(q_{\mathrm{m},1}-q_{\mathrm{m},2})/(\kappa_2-\kappa_1)$ for the first nucleic acid fragment and $(q_{\mathrm{m},1}-q_{\mathrm{m},2})/(\kappa_3-\kappa_1)$ for the second nucleic acid fragment within bounds specified by the measurement errors. We then found weighted mean values for both unknowns. We performed three sets of such an analysis which revealed average values of $\ln f_{\mathrm{H}}$ =0.007±0.025 or f_{H} =1.007±0.025 (Fig. S3b).

S3.2 Measuring the effective charge of a single dye molecule, q_{dye}

In order to determine the effective charge of a single dye molecule coupled to the end of the double helix, we performed in addition, measurements on dsDNA and dsRNA fragments labeled with a single ATTO 532 dye molecule coupled to a single 5' terminus. An isolated ATTO 532 dye molecule in solution carries a net structural charge, $q_{str}=-1e$ (Fig. S2a), which has been verified in prior measurements on single ATTO 532 molecules in solution[10]. The local electrostatic environment of the dye which arises as a result of coupling of the dye molecule into the electrostatic near field of the "parent" DNA molecule can however alter the effective charge of the dye. Therefore, despite the knowledge of the free solution effective charge of a single dye molecule, its effective charge in the molecular context, involving coupling to the end of a DNA fragment, is *a priori* not clear. We denote the effective charge of a dye molecule coupled to the terminal phosphate of a DNA fragment as q_{dye} which can not only be calculated but can also be directly determined via measurement.

In order to measure the value of q_{dye} we compared pairs of measurements of q_{m} for identical 60 bp dsDNA and dsRNA fragments carrying either one or two ATTO 532 dyes on their 5' ends. The difference between a pair of such measurements for a given molecular species permits us to directly estimate the effective charge contribution of a single ATTO532 dye molecule to the overall measurement. The measured difference is given by:

$$\Delta q = q_{\mathrm{m}} - q'_{\mathrm{m}} = f_{\mathrm{M}} q_{\mathrm{dye}} \quad \text{(S20)}$$

Here q'_{m} denotes the measured effective charge of a 60bp dsDNA or dsRNA labeled with a single dye only, while q_{m} is the measured effective charge of the same fragment labelled with two dyes.

In our experiments, a double labeled fragment n basepairs in length with fluorophores on both 5' ends carries a structural charge, $q_{\mathrm{str}}=-2ne$ (Fig. S2a). But in a singly labeled fragment one 5'-phosphate is unmodified. This terminal phosphate monoester group has two ionizable sites as opposed to a single ionizable site encountered, e.g., in the phosphodiester backbone as well as at a dye-derivatized 5' end phosphate (Fig. S2a). Nominally this would suggest that the structural charge of a dsDNA or dsRNA fragment labeled with a single fluorophore on one 5' end is $q_{\mathrm{NA}}=-(2n+1)\,e$. However, it is important to note that the two ionizable phosphate monoester sites have widely different nominal pK_{a} values of ≈2 and 7. The local electrical potential in the vicinity of the ionized phosphates is around 7-8 $k_{\mathrm{B}}T$ under our experimental conditions (≈1 mM alkali chloride, pH=9). This implies a local pH in the vicinity of the phosphate groups of around 5-6, which suggests that we can safely assume that the ionizable site in the phosphate monoester group will carry a magnitude of

structural charge less than 0.1 e (Fig. S2a). Charge renormalization will reduce the contribution of this weakly ionized group to the measured overall effective charge of the molecule to the level of around or less than 0.02 e. The latter is comparable with the standard error in the measurement of the effective charge of the dye, $\mathrm{SE_{dye}}$, and we therefore neglect explicit consideration of this contribution to our working estimate value of q_{dye}. Furthermore, any possible contributions of charge uncertainties at this level to the overall measurement of molecular geometric parameters are considered in the context of the analysis presented in Fig. S5 and Fig. S6 where we examine the impact on the final results of small perturbations of the value of q_{dye} around the measured value.

Having established values for f_{H} and $f_{\mathrm{M}}q_{\mathrm{dye}}$ we proceed as described below in order to determine q_{dye}. Rearranging equation (S15) we have:

$$f_{\mathrm{M}}q_{\mathrm{eff,n}} = q_{\mathrm{m,n}} - 2f_{\mathrm{M}}q_{\mathrm{dye}} + k_{\mathrm{B}}T\frac{\ln f_{\mathrm{H}}}{\phi_{\mathrm{m}}} \tag{S21}$$

where all the quantities on the right hand side, namely, the measured effective charge of the nucleic acid molecule n basepairs in length, $q_{\mathrm{m,n}}$, the measured effective dye charge, $f_{\mathrm{M}}q_{\mathrm{dye}}$, and the final term involving the hydrodynamic correction factor, are now known.

We use the values for $f_{\mathrm{M}}q_{\mathrm{eff,n}}$ inferred from equation (S21) for fragments of different lengths, n =30, 40 and 60, in the analysis procedure invoking the calculated effective charge values $q_{\mathrm{eff},n}(b,r)$ in order to extract values for three unknowns: b, r, and f_{M}. This procedure is discussed in detail in section S4. At this point, we note that the value of f_{M} obtained from the overall analysis yields a measurement for q_{dye}, since $f_{\mathrm{M}}q_{\mathrm{dye}}$ is known from the charge difference measurement described previously.

We performed three independent measurements on singly and doubly labeled B-DNA fragments and three measurements on A-RNA. We obtained the following average value for the measurements with B-DNA: $q_{\mathrm{dye,DNA}} = -0.46 \pm 0.07\ e$ where the standard error on the measurement, $\mathrm{SE_{dye,DNA}} = 0.04\ e$. Measurements for A-RNA reveal an average value of $q_{\mathrm{dye,RNA}} = -0.44 \pm 0.09\ e$, where $\mathrm{SE_{dye,RNA}} = 0.05\ e$.

We point out that our measured values for q_{dye} are in good agreement with calculated values of ca. $-0.48\ e$ and $-0.43\ e$ for $q_{\mathrm{dye,DNA}}$ and $q_{\mathrm{dye.RNA}}$ respectively, assuming the charge on the dye molecule can be modeled as a sphere of radius 5 Å, and that the distance of the sphere from the 5' end of the DNA molecule is 1 nm, which is given by the effective length of the linker (Fig. S2a).

S4. Inferring the values of radius, r, rise per base pair, b, and multiplicative correction factor, f_{M}, from measurements of effective charge

In this section, we describe the procedure used to extract the molecular properties of interest from our measured values of effective charge, $f_{\mathrm{M}}q_{\mathrm{eff,n}}$.

S4.1 Relationship between the measured effective charge of the double helix, q_{m}, and rise per basepair, b and radius, r

We model an *n* basepair nucleic acid double helix of radius, r, and axial rise per basepair, b, as a cylinder of radius $r_{\mathrm{cyl}} = r$ and length *nb*, carrying a total charge of $-2ne$. Using our previously described PB framework for calculating electrostatic free energies, F_{el}, and corresponding molecular effective charges, q_{eff}, we obtain a set of q_{eff} values for charged cylinders for a range of values corresponding to 2-5 Å for b and 2-30 Å for r, with a calculated uncertainty of around 0.1%[9]. We then fit the calculated $q_{\mathrm{eff},n}(b,r)$ values with a power-law function of the form $Q_n(b,r) = a_1 + a_2(b^{\alpha} + a_3)(r^{\beta} + a_4)$ such that the maximum deviation between the fit and the calculated data was

≈0.05% (Fig. S4a). Fig. S4a lists the values of all parameters in the three fit equations for $n = 30, 40$ and 60 determined for salt concentrations ranging from 1 to 1.5 mM. Comparison of partial derivatives $(Q_n)'_b$ and $(Q_n)'_r$ reveals a much stronger dependence of $q_{\mathrm{eff,n}}$ on b than that on r:

$$(Q_n)'_b = a_2 \cdot \alpha\left(r^\beta + a_4\right)b^{\alpha-1} \tag{S22a}$$
$$(Q_n)'_r = a_2 \cdot \beta(b^\alpha + a_3)r^{\beta-1} \tag{S22b}$$

Although $r^{\beta-1}$ can be slightly larger (≈10-15%) than $b^{\alpha-1}$ for implausibly small r values ≈2-3 Å and b ≈5 Å, the $\alpha\left(r^\beta + a_4\right)$ and $\beta(b^\alpha + a_3)$ factors play a decisive role here. For any parameter set from Fig. S4a, $\alpha\left(r^\beta + a_4\right)$ is ≈3-4 times larger than $\beta(b^\alpha + a_3)$ for any r and b within the ranges considered. Thus, we find that the axial rise per basepair, *b*, has a stronger influence on the measured effective charge of the molecule, compared to the radius of the cylinder, whose influence is more modest. This effect is borne out clearly in the measurements where we achieve far higher precision (≈0.1 Å) in the readout of the average *b* value, compared to the precision on the radius, *r*, which is on the order of 1 Å.

We now rewrite equation (S21) to relate the measured quantity from experiment to the molecular geometrical quantities of interest, embodied in the $q_{\mathrm{eff},n}(b,r) = Q_n(b,r)$ relations:

$$q_{\mathrm{m,n}} - 2f_{\mathrm{M}}q_{\mathrm{dye}} + k_{\mathrm{B}}T\frac{\ln f_{\mathrm{H}}}{\phi_{\mathrm{m}}} = f_{\mathrm{M}}Q_n(b,r) \tag{S23}$$

The above equation has three unknown parameters: f_{M}, r and b, all on the right side of the equation. Parameters r and b describe the structure of the molecule while f_{M} describes the experimental apparatus. In order to determine these three unknowns, we require at least three measurements of $q_{\mathrm{m,n}}$ on fragments of different lengths that are described by identical values of r and b. In practice, this requires four measurements in total: three $q_{\mathrm{m,n}}$ measurements for three different fragments and occasionally (25% of all measurement sets) an additional measurement of a singly labeled fragment in order to determine $f_{\mathrm{M}}q_{\mathrm{dye}}$ as described in the previous section. To keep f_{M} constant, all the measurements needed to be performed under identical conditions. In particular, we used the same measurement device and buffer conditions with effectively identical pH and salt concentration, c. We also point out that our measurements of $q_{\mathrm{m,n}}$ include statistical uncertainties $q_{\mathrm{e,n}}$ arising primarily from the uncertainties on measured t_{esc} values.

Note that we do not explicitly consider uncertainties on the measured quantities $f_{\mathrm{M}}q_{\mathrm{dye}}$ and f_{H} at this stage. Since these two quantities appear in an additive fashion in the final $q_{\mathrm{m},n}$ value of interest, we consider their uncertainties in a consolidated fashion. In particular, we explore the impact of small perturbations on the overall additive quantity, $f_{\mathrm{A}} = 2q_{\mathrm{dye}} - k_{\mathrm{B}}T \cdot \ln f_{\mathrm{H}}/\phi_{\mathrm{m,t}}$, on the final results for r and b. Fig. S5 and Fig. S6 illustrate the results of this study. We find that perturbations $\leq \pm 2\mathrm{SE}_{\mathrm{dye}}$ on the value of q_{dye} preserve the qualitative measured trends for the radius, r, in most of the cases (Fig. S5a and Fig. S6a). The results for the rise per basepair, b, on the other hand, remain robust to such perturbation (Fig. S5b and Fig. S6b).

For convenience, we introduce variable names q_{30}, q_{40} and q_{60} in place of $q_{\mathrm{m,n}} - 2f_{\mathrm{M}}q_{\mathrm{dye}} + k_{\mathrm{B}}T \cdot \ln f_{\mathrm{H}}/\phi_{\mathrm{m,t}}$ for n=30, 40 and 60 respectively. In order to simplify the problem and eliminate f_{M}, which is a common multiplicative factor in all the measurements in a given experiment, we perform pairwise division of three equations denoted by equation (S23). Thus, we obtain two equations with two unknowns, r and b:

$$\frac{q_{30}}{q_{60}} \pm 3\sqrt{q_{e,30}^2 + q_{e,60}^2} = \frac{Q_{30}\,(b,r)}{Q_{60}\,(b,r)} \tag{S24a}$$

$$\frac{q_{40}}{q_{60}} \pm 3\sqrt{q_{e,40}^2 + q_{e,60}^2} = \frac{Q_{40}\,(b,r)}{Q_{60}\,(b,r)} \tag{S24b}$$

Once r and b are known, using the simulation based approach described in the following section S4.2, we determine f_{M} by substitution in one of the original equations.

The value of f_{M} obtained for data sets that include singly labeled nucleic acid fragments enables us to estimate values for q_{dye} as described in section S3.2. Knowing the values for $q_{\mathrm{dye,DNA}}$ and $q_{\mathrm{dye,RNA}}$ permits us to simplify all further measurements and analysis by rearranging equation (S23) as follows:

$$q_{\mathrm{m,n}} + k_{\mathrm{B}}T\,\frac{\ln f_{\mathrm{H}}}{\phi_{\mathrm{m}}} = f_{\mathrm{M}}[Q_n(b,r) + 2q_{\mathrm{dye}}] \tag{S25}$$

We now have:

$$\frac{q_{30}{}^*}{q_{60}{}^*} \pm 3\sqrt{q_{e,30}^2 + q_{e,60}^2} = \frac{Q_{30}\,(b,r)^*}{Q_{60}\,(b,r)^*} \tag{S26a}$$

$$\frac{q_{40}{}^*}{q_{60}{}^*} \pm 3\sqrt{q_{e,40}^2 + q_{e,60}^2} = \frac{Q_{40}\,(b,r)^*}{Q_{60}\,(b,r)^*} \tag{S26b}$$

where $q_{30}{}^*$, $q_{40}{}^*$ and $q_{60}{}^*$ denote $q_{\mathrm{m,n}} + k_{\mathrm{B}}T \cdot \ln f_{\mathrm{H}}/\phi_{\mathrm{m,t}}$ where n=30, 40 and 60 respectively. Furthermore, $Q_{30}\,(b,r)^*$, $Q_{40}\,(b,r)^*$ and $Q_{60}\,(b,r)^*$ represent $Q_n(b,r) + 2q_{\mathrm{dye}}$ for n =30, 40, 60. We solve the system of two equations (S26a) and (S26b) using the same algorithm as for equations (S24a) and (S24b) as described in the next section S4.2.

S4.2 Algorithm for determining rise per base pair, *b* and radius, *r* of the double helix

In this section we describe the procedure we use to extract the values of the unknowns of interest using equations (24a) and (24b) (or equations (26a) and (26b)). The algorithm was developed and validated using simulated values of measured molecular effective charge, $q_{\mathrm{s,n}}$, where the subscript 's' emphasizes the fact that these values are calculated or simulated for the purpose of developing the algorithm, and not measured in experiment. In order to do so, we begin with $q_{\mathrm{s,n}}$ values calculated for any set of input parameters, $r = r_{\mathrm{in}}$, $b = b_{\mathrm{in}}$, $f_{\mathrm{M}} = f_{\mathrm{M,in}}$, as given by the equations $q_{\mathrm{s,n}} = f_{\mathrm{M}} Q_n(b,r)$, where $Q_n(b,r) = a_1 + a_2(b^{\alpha} + a_3)(r^{\beta} + a_4)$ for each fragment. We then superimpose an amount of charge corresponding to ±0.2% relative error on the value for each fragment, so that an input value of the form $q_{\mathrm{s,n}} \pm 0.006 q_{\mathrm{s,n}}$ represents a measured mean value, $q_{\mathrm{s,n}}$, with Gaussian distributed uncertainty characterized by a standard deviation of $0.002 q_{\mathrm{s,n}}$. Such values are analogous to experimental measurements of the form $q_{\mathrm{m,n}} \pm 3q_{\mathrm{m,e}}$, where $q_{\mathrm{m,e}}$ is about 0.2% of $q_{\mathrm{m,n}}$. We applied these simulated input values to our analysis routine and sought to establish an algorithm that returned the known inputs for b, r and f_{M}.

Initially, we determined all possible combinations of b and r values that satisfied each of the two equations separately, as follows. Each effective charge ratio, i.e., $q_{\mathrm{s,30}}/q_{\mathrm{s,60}}$ and $q_{\mathrm{s,40}}/q_{\mathrm{s,60}}$, excluding errors can be thought of a 2D surface parallel to both r and b axes. But in practice, these mean values are broadened by errors, such that the standard deviations on each of the input ratios can be written as $q_{e,1} = \sqrt{(0.002q_{s,30})^2 + (0.002q_{s,40})^2}$ and $q_{e,2} = \sqrt{(0.002q_{s,40})^2 + (0.002q_{s,60})^2}$ respectively. Including uncertainties corresponding to three standard deviations yields a probability weighted input written as $q_{\mathrm{s,30}}/q_{\mathrm{s,60}} \pm 3q_{e,1}$ and $q_{\mathrm{s,40}}/q_{\mathrm{s,60}} \pm 3q_{e,2}$. Intersection of the ranges of the two simulated input values with the calculated quantities given by $Q_{30}\,(b,r)/Q_{60}\,(b,r)$ and

$Q_{40}\ (b,r)/Q_{60}\ (b,r)$ (right hand sides of equations S24a and S24b) gives two independent manifolds of solutions in b-r space as illustrated in Fig. S4b. The analyses were performed using calculated surfaces plotted using a grid resolution of 0.01 Å in b and r.

The intersection of the above two manifolds yields a final probability weighted space of solutions in b and r which are common to the system of three input simulated measurement values. Each member of the final "b-r manifold" solution space (Fig. S4b lower panel) is associated with a probability tag that reflects the product of individual probabilities of the "overlapping" values. Because the uncertainties in the measurement resulted in relatively broad b-r manifolds, we found that an unrestricted direct weighted average determination of inferred output values for the rise per basepair and helical radius (b_{out} and r_{out} respectively) did not accurately and consistently recover the known inputs b_{in} and r_{in}.

We therefore developed and validated the following procedure to recover the known inputs, b_{in} and r_{in}. We first focus on estimating the rise per basepair, b. Using the final b-r manifold for a given input pair ($b_{\mathrm{in}}, r_{\mathrm{in}}$), we calculated a weighted average of b (which we term b_{out}) over a range in r corresponding to r =6 to 30 Å. This procedure was carried out for various sets of b_{in} and r_{in} ranging from 2-4 Å for b, and 8-18 Å for r, with a step size of 0.1 Å for b and 0.4 Å for r. We rounded the obtained b_{out} values to the nearest 0.1 Å. We found that this procedure consistently yielded b_{out} values that accurately reflected the specific b_{in} input value, departing from the inputs by 0.2 Å at the most (Fig. S4c.I). Fig. S4c illustrates the overall accuracy of the method for different sets of tested b_{in} and r_{in} parameters. For nominal values of b=3.4 Å for DNA and 2.6 Å for RNA, the accuracy of the procedure, as assessed by the ratio $b_{\mathrm{out}}/b_{\mathrm{in}}$, is 1.0 if the corresponding r_{in} values are within the ranges of 10.5-17.0 Å and 11.3-16.5 Å respectively. Outside these optimal ranges, we note that b_{out} can exceed b_{in} by 0.1-0.2 Å when r_{in} lies below the optimal range. On the other hand, we find that b_{out} is smaller than b_{in} by about 0.1 Å when the corresponding r_{in} value lies above the optimal range (Fig. S4c.I).

The goal of the next analysis step is to establish a procedure to infer the helical radius, r. Here we use the b-r manifolds obtained for various input pairs ($b_{\mathrm{in}}, r_{\mathrm{in}}$), and construct a probability distribution in r, restricting the value of b to b_{in}. We then determined the value of r_{out} by fitting a Gaussian function to the region of the first peak in the probability distribution of r values, similar to that shown in Fig. S4b (bottom panel). This value was consistently found to lie within 1 Å of r_{in} over a wide range of r_{in}, e.g., 6-20 Å. This implies that as long as we can accurately determine b_{out}, such that $b_{\mathrm{out}}/b_{\mathrm{in}} = 1.0$, we can determine r_{out} with <1 Å accuracy. In practice, when dealing with experimental data we do not have access to b_{in}, but rather only to b_{out}, obtained by the approach described above.

The analysis clearly indicates that the accuracy in the determination of r depends on the accuracy in the determination of b. Fig. S4c.II demonstrates that when $b_{\mathrm{out}}/b_{\mathrm{in}}$ =1.0 the obtained r_{out} values lie within ±10% of the input r_{in}. However when b_{out} is either under- or overestimated by ≈4% (≥0.1 Å) we find that r_{out} is correspondingly under- or overestimated by ≈40% compared to the known input value (Fig. S4c.II). For instance, for small r_{in} values <10 Å the method entails an overestimation of b_{out} by at least 0.1 Å which in turn implies overestimated values for $r_{\mathrm{out}} \approx 1.4\, r_{\mathrm{in}}$. Thus, our simulation procedure is not capable of accurately recovering r_{out} values for r_{in} <10 Å. We comment on the implications of this observation for our results, particularly in the context of A-RNA, at the end of Section 4.3. Furthermore, when the simulation-vetting procedure on occasion yields a value of r_{out} <10 Å, we find that b_{out} has been underestimated, i.e., $b_{\mathrm{out}} = b_{\mathrm{in}} - 0.1$ Å. In these cases, we also find that correcting the b_{out} value upwards by 0.1 Å yields an r_{out} value that is within 1 Å of r_{in}.

Once r_{out} and b_{out} have been determined using the above procedure, the multiplicative correction factor, $f_{\mathrm{M,out}}$, is then determined by substituting the obtained values for b_{out} and r_{out} in

any of the three equations $q_{\mathrm{s,n}} = f_{\mathrm{M}} Q_n(b, r)$. Performing the above data analysis procedure using measured inputs q_n or q_n^* in equations (S24a) and (S24b) or (S26a) and (S26b) respectively, we obtain all our measurables b_{m}, r_{m} and f_{M}. Note that equations (S24a) and (S24b) are used for datasets where q_{dye} is also a measurable, and equations (S26a) and (S26b) are used in cases where q_{dye} is known from a previous measurements.

We found that the results for r_{m} and the trends of r_{m} vs a_{H} depended sensitively on the exact value of the additive correction factor, $f_{\mathrm{A}} = 2q_{\mathrm{dye}} - k_{\mathrm{B}}T \ln f_{\mathrm{H}}/\phi_{\mathrm{m,t}}$. Since the uncertainty on q_{dye} can be estimated more accurately than that on $\ln f_{\mathrm{H}}$, we first focus on the impact of the measurement uncertainty on q_{dye} given by $\mathrm{SE}_{\mathrm{dye}}$. We therefore varied the value of q_{dye} in the range of $q_{\mathrm{dye,m}} \pm 2\mathrm{SE}_{\mathrm{dye}}$ in increments of $\mathrm{SE}_{\mathrm{dye}}$. We repeated the *b-r* analysis described above with these perturbed values of q_{dye} and determined the corresponding r_{m} and b_{m} values for each dataset using our simulation-vetted procedure described above. The results are shown in Fig. S5. Clearly measurements of the rise per basepair, b_{m}, are relatively insensitive to value of q_{dye} (Fig. S5b). The measured values for r_{m} in turn are more sensitive to the particular choice of q_{dye} (Fig. S5a). We found that while most cases display a direct dependence of r_{m} on a_{H}, the results corresponding to $q_{\mathrm{dye}} = q_{\mathrm{dye,m}} + \mathrm{SE}_{\mathrm{dye}}$ show the clearest trends for both A-RNA and B-DNA. It is therefore likely that $q_{\mathrm{dye}} = q_{\mathrm{dye,m}} + \mathrm{SE}_{\mathrm{dye}}$ approximates closest the true value of q_{dye}, and we focus on the results corresponding to $q_{\mathrm{dye}} = q_{\mathrm{dye,m}} + \mathrm{SE}_{\mathrm{dye}}$ for all analyses and discussions in the study.

In addition, we further examined the impact of an altered value of $\ln f_{\mathrm{H}}$ on the measured b_{m} and r_{m} results keeping the value of q_{dye} constant (Fig. S6). For example, in place on the value $\ln f_{\mathrm{H}}$=0.007 used in the analysis in Fig. S5 we used a value nearly a factor 2 larger corresponding to $\ln f_{\mathrm{H}}$=0.013. Again we found that the values obtained for b_{m} remained effectively unchanged. We also observed a robust linear dependence of r_{m} on a_{H}, with a small difference however in the $r_{0,\mathrm{A}}$ and $r_{0,\mathrm{B}}$ intercept values in each case with respect to the previous results. The intercept values in this analysis turned out to be overall about 1 Å smaller than in the previous analysis procedure. The values however lie within each other's estimated bands of uncertainty (compare tables in Fig. S5 and Fig. S6).

All the measured r_{m} and b_{m} values obtained with the method described in this section for A-RNA and B-DNA fragments in CsCl, RbCl, NaCl and LiCl salt solutions are listed in Table S1 along with the corresponding experimental details for all measurements. Fig. S7 presents the results for representative datasets in each salt species for both B-DNA and A-RNA fragments.

S4.3 Simulation based studies of the role of the multiplicative correction factor, f_{M}, in the measurement of b and r

Assuming particular values of $f_{\mathrm{M,in}}$, b_{in} and r_{in} as known inputs we obtain simulated effective charge values for all three fragments $q_n = f_{\mathrm{M,in}} Q_n(b_{\mathrm{in}}, r_{\mathrm{in}})$. We ran the data analysis procedure described in the sections above using these values in equations (S24a) and (S24b) for a range of values b_{in}(2-4 Å), r_{in}(8-18 Å) and f_{M} chosen as a random positive value. As indicated above, the vetting of the analysis procedure in this fashion revealed that there is an optimal range for r_{in} over which the analysis delivers output values $f_{\mathrm{M,out}}$, b_{out} and r_{out} within 4%, <1% and 10% of the inputs respectively. For example, for nominal values of b_{in}=3.4 Å and 2.6 Å (typical for B-DNA and A-RNA) the optimal range in r_{in} that yields <10% error in determining r_{out} (i.e., $|(r_{\mathrm{out}} - r_{\mathrm{in}})/r_{\mathrm{in}}| < 0.1$) lies between r_{in} =10.5-17.0 Å and 11.3-16.5 Å respectively. In this range, we obtain high accuracy in the determination of b_{out} where we find that $b_{\mathrm{out}}/b_{\mathrm{in}}$ =1 (Fig. S4c.I), and the accuracy in the determination of f_{M} is better than 4% ($|(f_{\mathrm{M,out}} - f_{\mathrm{M,in}})/f_{\mathrm{M,in}}| < 0.04$) (Fig.S4c.III). For r_{in} less than 10.4 Å we note that $1.1 < \frac{r_{\mathrm{out}}}{r_{\mathrm{in}}} < 1.8$ (Fig. S4c.II). However, it is worth noting that even when the accuracy of the analysis in *r* is poor, as reflected in the ratio $r_{\mathrm{out}}/r_{\mathrm{in}} \approx 1.8$, we obtain

$f_{\mathrm{M,out}}/f_{\mathrm{M,in}} \approx 0.8$ and $b_{\mathrm{out}}/b_{\mathrm{in}} \approx 1.08$ (Fig. S4c). The accuracy in determining b stems from the higher sensitivity of q_{eff} to b compared to r as discussed earlier.

We point out that the value for f_{M} serves as an important diagnostic purpose in our ability to distinguish between model-IA and model-IIA of A-RNA. In principle, for a true value of r=5 Å our data analysis procedure would naturally provide an overestimate of $r_{\mathrm{m}} \approx$15 Å, which is larger by a factor three than the true value. However, the analysis procedure also shows that this overestimate of r is coupled with a large underestimate of f_{M} of the order of 30% relative to any input value of $f_{\mathrm{M,in}}$. In practice, $q_{\mathrm{m,n}}$ measurements performed for B-DNA and A-RNA fragments under effectively identical conditions, using the same measurement device, pH and salt concentration, c, reveal very close f_{M} values for both helical forms. E.g., for RbCl measurements, $f_{\mathrm{M,DNA}}$ =0.902, corresponding to r_{m} =12.4 Å and b_{m} =3.2 Å, is very close to $f_{\mathrm{M,RNA}}$ =0.919, where r_{m} =13.3 Å and b_{m} =2.6 Å. Assuming that the true radius of B-DNA is not much smaller than 10 Å and that the B-DNA measurement therefore reflects an accurate result, such close agreement in the f_{M} values implies that the obtained r_{m} values for A-RNA lie in the range where we expect about 10% uncertainty in the inferred r values, as is typical of the method (Fig. S4c.II and Fig. S4c.III). Were the true value rather r=5 Å, this would be immediately reflected in the value of $f_{\mathrm{M,RNA}}$ which, according to our analysis vetting procedure, would be at least 30% smaller than $f_{\mathrm{M,DNA}}$. Clearly this is not the case, permitting us to infer comparable accuracy for r measurements for both A-RNA and B-DNA.

S4.4 The influence of ionic species on f_{M}

In practice, although we can determine f_{M} reasonably accurately for every single measurement, unrestricted comparison of f_{M} across all measurements is not very meaningful as the major determinant of f_{M} stems from experimental variations such slit height uncertainty, h_{e}. But measurements performed under identical conditions given by salt concentration and slit height (same measurement device) may be directly compared to test for relative departures of f_{M}. We expect such a comparison to carry information on any variation in surface electrical potential that may arise solely on account of a single known variable factor in the experiments in question, say the cationic species in solution.

In order to compare the effect of different counterion species on f_{M}, we performed f_{M} measurements for the same nucleic acid species in different alkali metal chloride solutions, keeping all other experimental conditions identical, including salt concentration. Comparing f_{M} values, averaged over all measurements performed under identical conditions, we indeed noted slightly different f_{M} values as a function of cationic species. We found that the values for f_{M} increased in the order Cs≈Rb<Na<Li. We also found that our values for the quantity $f_{\mathrm{M}}\phi_{\mathrm{s}} = \phi_{\mathrm{s,t}}$, the true effective surface potential in our experiments, are in good agreement with zeta (ζ) potentials reported for silica surfaces in alkali metal chloride solutions, as shown in Fig. S8[12]. Our $\phi_{\mathrm{s,t}}$ values are of course higher in magnitude than the corresponding ζ potential values in each case, but this is to be expected since: (1) the ζ potential reflects the value of the electrical potential at the shear surface near a particle, which can be distinct and further away than its geometric surface, and (2) samples of silica may vary greatly in their surface electrical properties depending on preparation and treatment procedures[13].

S5. Dependence of the inferred r_{m} vs. a_{H} relationship on the assumed values of the hydrated cationic radii, a_{H}

Fig. 4 demonstrates that r_{m} values for both DNA and RNA vary linearly with hydrated radii of the cationic species, a_{H}, in solution. We assumed values for a_{H} taken from Ref. 18. These values were determined from limiting ionic conductance values using both slip and stick hydrodynamic conditions at the surfaces of spheres of radii, a_{H}, that represent a migrating ionic entity which is

assumed to be spherical in solution. The Stokes drag (friction coefficient) on an ion in solution is given by $\gamma_s \pi \mu a_H$, where $\gamma_s = 6$ for the stick boundary condition and $\gamma_s = 4$ for the slip case. For stick boundary conditions the reported Stokes radii were smaller than the crystal ionic radii for Rb^+ and Cs^+ (e.g., r_{Cs} =1.65 Å and $a_{H,Cs}$ =1.19 Å; r_{Rb} =1.49 Å and $a_{H,Rb}$ =1.18 Å). In contrast, for slip boundary conditions the reported a_H values for Rb^+ and Cs^+ are comparable to the crystal ionic radii ($a_{H,Cs} = 1.79$ Å and $a_{H,Rb} = 1.78$ Å), and are therefore considered more plausible than the former case. Fig. S9a illustrates the r_m vs. a_H dependence for hydrated ionic radii obtained using slip boundary conditions from Ref. 18, as shown in Fig. 4a. Linear extrapolation of the measured r_m values to $a_H = 0$ yields $r_{0,A}$ =10.5±0.6 Å and $r_{0,B}$ =11.8±0.6 Å for A-RNA and B-DNA respectively (Fig. S9a), as presented in the main text.

As an independent verification of the a_H values in Ref. 18, we analyzed our electrical conductivity measurements as a function of salt concentration (Fig. S2d), which were used to monitor the salt concentration in each measurement. We fitted the data with the equation $c = A + B\gamma$, where A and B are fit coefficients and γ (in $\mu S/cm$) is the measured conductivity of a solution with salt concentration, c (in mM). In order to determine the values for hydrated cationic radii we used the fitted values for slopes, B=$4\pi\mu R_i / 10^4 e_0^2 N_A$, since

$$c = \frac{4\pi\mu R_i}{e_0^2 N_A}(\gamma - \gamma_{sol}), \text{ with } \frac{1}{R_i} = \frac{1}{a_{Cl^-}} + \frac{1}{a_H} \quad \text{(S27)}$$

Here, a_H is the hydrated radius of the cationic species, a_{Cl^-} is the hydrated radius of Cl^-, γ_{sol} denotes the conductivity of pure solvent, and γ (in $\frac{S}{m}$) is the measured conductivity of the solution with salt concentration, c (in mM). The value for a_{Cl^-}=1.81 Å was taken from McMillan's study[14]. Fig. S2d lists our measured values for hydrated cationic radii, a_m, which lie between 8-12% of the values in Ref. 18.

Furthermore, we explored the dependence of r_m on a_H values from two other literature sources. Nightingale used a calibration curve to convert Stokes radii from conductance measurements to hydrated radii of ions in solution[15]. This was done by taking Stokes radii measurements using stick boundary conditions (γ_s=6) for four cations in the series from tetraethyl- to tetrapentylammonium. These values were related to the corresponding values of hydrated radii that were in turn simply taken to be their crystal radii. These large cations were considered to be non-hydrated, so that their crystal radii were assumed to reflect their true hydrated radii in aqueous solutions. The calibration curves thus obtained was extrapolated to the regime of small Stokes radii and the a_H values for smaller ions, e.g., alkali metal ions with Stokes radii <2.82 Å (Stokes radius for tetraethylammonium cation) were read off from this curve. Nightingale's a_H values vary from 3.29 to 3.82 Å for cations in the order $Rb^+ \rightarrow Li^+$, while the values in Ref. 18 lie in the range 1.78-3.58 Å. Fig. S9b displays the r_m vs. a_H dependence for Nightingale's a_H values and the corresponding linear fit functions with a shared value of slope for both helical forms. Extrapolation of the fit to $a_H = 0$ leads to very small values for both $r_{0,B}$ and $r_{0,A}$ (2.8 and 4.1 Å respectively). Furthermore, the slope of the fit function is significantly larger than 1.

We performed a similar analysis using a_H values obtained by Marcus[16]. Values for a_H in this study were calculated using a model which links the ionic radius, the width of the hydration shell and the number of molecules in this shell with the values for standard molar Gibbs free energies of hydration. Marcus' values for hydrated ionic radii are smaller than Nightingale's and again lie within a fairly narrow range: 2.13-2.42 Å. Linear fits of the r_m vs a_H data once again lead to very small values for $r_{0,B}$ and $r_{0,A}$ (1.2 and 2.5 Å) and the slope of the r_m vs. a_H fit line is around 5 (Fig. S9c).

S6. Comparing the radii of two forms of the double helix: analysis of model structures of B-DNA and A-RNA

The 3DNA web server 2.0 was used to generate generic fiber model structures (derived from a database of published experimental crystallographic data) of B-DNA and A-RNA of the desired sequence (Fig. S2a)[17]. The B-DNA and A-RNA models we use have a helical rise per basepair of 3.4 and 2.6 Å respectively. These atomic structures were used to generate molecular surface structures which were then imported into the mesh generation package GMSH. Finite element numerical calculations were then performed as described in the following section.

The 'main backbone' atoms in the molecular visualization software packages used for the structural analysis are the C3' and C4' atoms for DNA and RNA respectively[18, 19]. The ribbon structures trace out these atomic positions (Fig. S10a). The average distance of these main backbone atoms was calculated to be around 7.9 Å for B-DNA and 9.5 Å for A-RNA. Considering all backbone carbon atoms, namely, C3', C4', C5', the corresponding values are 7.9±0.3 Å for B-DNA and 9.3±0.7 Å for A-RNA. Taking all possible backbone atoms (P, O1P, O2P, O3', O5', C3', C4', C5'), however, we find that the mean atomic distances from the principal axes of the structures are 8.7±0.9 Å for B-DNA and 8.9±0.9 Å for A-RNA. All the above cases provide estimates of a helical radius where the value for A-RNA is systematically larger than for B-DNA. However, considering only the spatial distributions of the P atoms, we obtain values for the average distance from the helical axis that are more similar for the two structures; we note a value of 9.23±0.01 Å for B-DNA that is in fact slightly larger than the average distance of 8.6±0.6 Å obtained for A-RNA. Next we consider the axial radius of gyration which in turn provides a measure of the average displacement of all atoms from the principal axis. The radius of gyration of A-RNA is approximately 7.8 Å and is around 1 Å larger than the value of 6.7 Å characterizing B-DNA. Overall we find that the backbone carbon atoms and the axial radius of gyration point to values that indicate that the helical radius of A-RNA is about 1 Å larger than B-DNA. Furthermore, we point out that the axial radii of gyration of the molecular surfaces generated by a probe of radius of 1 Å, using default van der Waals (vdW) atomic radii ($w = 0$), are 6.5 Å and 7.8 Å for B-DNA and A-RNA, respectively (model-IB and model-IA of Fig. 2). These values are in fact very similar to the all-atom axial radii of gyration values above. Clearly, an enlargement of all atomic radii of all atoms ($w > 0$) would lead to a further increase in these values. E.g., Figure 2A further displays two additional molecular surfaces for B-DNA (w=1.4) and A-RNA (w=3 Å). The radii of gyration of these surfaces are 7.7 Å and 9.6 Å, respectively.

S7. Electrostatic modeling and free energy calculations for B-DNA and A-RNA

S7.1.1 Governing Equations

We first solved the non-linear Poisson-Boltzmann (PB) equation in the experimental geometry for two states of the molecule in the system: the "slit" state (#1) and the "pocket" state (#2) as shown in Fig. 1a and Fig. S10c. The PB equation is given by

$$-\nabla \cdot (\varepsilon\varepsilon_0 \nabla\phi) = \rho_\mathrm{e} \tag{S28}$$

where ϕ is the local electrical potential in the system, ρ_e is the charge density, and $\varepsilon\varepsilon_0$ is the permittivity of the medium. The charge density, ρ_e, in the aqueous solution is described by the Boltzmann distribution for ionic species:

$$\rho_\mathrm{e} = \sum_i c_i N_\mathrm{A} e z_i \exp\left(\frac{-z_i e\phi}{k_\mathrm{B}T}\right) \tag{S29}$$

where N_A is Avogadro's number, and c_i and z_i are the bulk concentration and the valences of the ith ionic species, respectively. Here $z_+ = -z_- = 1$ holds for a binary, symmetric and monovalent electrolyte.

Fig. 1a schematically represents a nucleic acid molecule in a nanofluidic device consisting of a nanoslit and a pocket region which serves as an electrostatic trap. Thermodynamically, the direction of the trapping process is determined by the difference in the free energy of the system between two different configurations, namely a "slit-state" (Fig. S10c, panel 1) and "pocket-state" (Fig. S10c, panel 2). Neglecting the orientational and spatial degrees of the freedom of the molecule, the problem can be simplified to the calculation of the free energy for two molecular configurations at the locations of minimum axial free energy showed in Fig. S10c. The free energy functional for our problem for any fixed state of the molecule is given by[20, 21]:

$$F_{\mathrm{el}} = \int_{\mathrm{V}} \left[\frac{\varepsilon\varepsilon_0}{2} \boldsymbol{E} \cdot \boldsymbol{E} - 2cN_{\mathrm{A}}k_{\mathrm{B}}T(\cosh\psi - \psi\sinh\psi - 1) \right] dV \quad \text{(S30)}$$

where $\boldsymbol{E} = -\nabla\phi$ is the electric field, and $\psi = e\phi/k_{\mathrm{B}}T$ is the nondimensional electrical potential.

In this study, we use the finite element (FE) approach to solve equation (S28) for DNA and RNA structures. We consider macroscopic molecular representations based on solvent excluded and solvent accessible surfaces (SES and SAS) deduced from atomic structures. Subsequently, we calculate free energy differences between the two states of the molecule. Note, that in these calculations the molecule is placed at the midplane of the parallel plate gap in the slit and pocket regions (i.e., $z = z_{\mathrm{max}}/2$), with its long axis parallel to the midplane of the slit (i.e., $\theta = 90°$). We then deduce the effective charge of the molecule from the relationship $\Delta F_{\mathrm{el}} = q_{\mathrm{eff}}\phi_{\mathrm{m}}$. Here $\Delta F_{\mathrm{el}} = F_1 - F_2$ is the difference in electrostatic free energy between the two states of the system, as calculated using equation (S30), and ϕ_{m} is the electrical potential at the midplane of the slit region in the absence of the molecule[21].

We then compare the effective charge values obtained for the molecular models with those of charged cylinders in order to deduce the equivalent radius, r_{cyl}, of a cylindrical object which leads to the same value of effective charge, q_{eff}, as described in sections S7.3 and S7.4. Further discussion on equivalent cylinders is provided in section S7.5.

S7.1.2 Boundary Conditions for the solution of the PB equation

We solve equation (S28) using Neumann boundary conditions that specify constant charge densities at the surfaces of the cylinder or charged atoms in the molecular structure. Note that constant charge on DNA is an excellent approximation given the highly acidic nature of the phosphate groups (pK_a≈2) under the conditions of our experiment (pH≈9). The effect of charge regulation of the silica walls due to the proximity of the charged molecule has a negligible impact on the calculated electrostatic free energy (F_{el}) which turns out to be essentially identical to the value obtained for constant charge boundary conditions. We therefore treat the silica walls with constant charge boundary conditions in both states involving the molecule in either the slit or the pocket region. For the silica surfaces of the device we have $-\varepsilon\varepsilon_0\nabla\phi \cdot \mathbf{n} = \sigma_{\mathrm{w}}$ at the boundary, where $\sigma_{\mathrm{w}} = -0.1$ e/nm^2 (giving $\psi_{\mathrm{m}} \approx -0.1$, similar to experiments) is a nominal surface charge density, and $\mathbf{n}$ is the normal vector pointing into the electrolyte.

While the application of this boundary condition is straightforward for cylinders and for the solid surfaces of the device, the process of defining a molecular surface is more challenging owing to the complicated, corrugated nature of a molecular surface constructed from atoms. Traditionally, there are 3 methods to define a molecular surface: (i) van der Waals (vdW) surface, (ii) Solvent Accessible Surface (SAS), and (iii) Solvent Excluded Surface (SES)[22]. The vdW surface of the molecular structure is simply the surface of the volume obtained by the union of all atoms in the molecule which are treated as spheres of radii given by each atom's respective vdW radius. The SAS in turn is a

continuous surface generated by the center of a spherical probe of radius r_{p} rolled over the vdW surface. It can be readily demonstrated that the SAS is equivalent to a vdW surface where the vdW radii of all atoms are increased uniformly by an amount equal to r_{p}. The SES, however, is defined by tracking the contact points of the rolling probe as shown in Fig. S10f. Accordingly, the SES can be regarded as a smooth manifold which approximates the true vdW surface as $r_{\mathrm{p}} \rightarrow 0$. One of the advantages of the SES is that it produces a smooth geometry which is favorable for mesh generation and finite-element analysis. For the above reasons, we use a SES created by a probe size of $r_{\mathrm{p}} = 1$ Å in order to generate the effective vdW surface of the molecular structures (Fig. 2). In our work we regard the vdWS and SES as synonymous. In order to create the SAS, we simply generate a SES of a molecular structure where the default vdW radii values of all atoms are increased by a constant value, w.

MSMS[23] is a molecular surface rendering software package which generates a SES by rolling a spherical probe of radius r_{p} over the surfaces of spheres representing atoms in the molecular structure. The radii of these spheres are taken to be equal to the corresponding vdW radius of each atom. MSMS is embedded as a third-party package into UCSF Chimera[24] which is a molecular visualization software. We point out that Chimera has different algorithms to determine the vdW radii of atoms based on whether the structure contains explicit H-atoms or not. In our case, since the atomic structures generated by 3DNA[25] do not contain explicit hydrogen atoms, "united atom radii" based on protein organic set (ProtOr) are used[26]. For more detail, we refer the reader to the Chimera User's Guide: https://www.cgl.ucsf.edu/chimera/docs/UsersGuide/midas/vdwrad.html.

In the present study, we consider 30 bp DNA and RNA molecules carrying a total structural charge of $q_{\mathrm{str}} = -60e$. Since the structural charge of the molecule is localized at the negatively charged O atoms in the backbone phosphate groups, we distribute the total charge over the spheres corresponding to the O1P and O2P atoms using the following procedure. We identify all surface patches in the molecular surface that are made up of points lying at a minimum distance from the nearest "O1P" and "O2P". This essentially distinguishes the surfaces of the charge carrying O backbone atoms from all other atoms in the structure. We then apply a Neumann boundary condition of the form $-\varepsilon\varepsilon_0\nabla\phi \cdot \mathbf{n} = \sigma_{\mathrm{p}}$ to the "O1P-O2P" patches, where $\sigma_{\mathrm{p}} = q_{\mathrm{str}}/A_{\mathrm{p}}$ is a charge density, and A_{p} represents the total surface area of all patches. We utilized GMSH[27] for the subsequent mesh generation and solved the governing equations using FEniCS[28] which is an open-source FE software package.

S7.3 Domain decomposition of the electrostatic free energy, and relating the double helix to an equivalent cylinder model

We solved equation (S28), and calculated the difference in free energy, ΔF_{el}, discussed above (Fig. S10c) for molecular helices and charged cylinders. These free energies were used to obtain values for $q_{\mathrm{eff}} = \Delta F_{\mathrm{el}}/\phi_{\mathrm{m}}$ and $\eta = q_{\mathrm{eff}}/q_{\mathrm{str}}$ which represent the effective charge and charge renormalization factor, respectively[9]. We are interested in mapping the molecular electrostatic problem onto a simpler case given by a cylindrical object of radius, r_{cyl}, and length, l. For a rise per base pair, b, the length of the corresponding cylinder is given by $l = nb$, where n represents the number of basepairs. In our model, the total structural charge is uniformly distributed over the curved surface of the cylinder; i.e. $\sigma_{\mathrm{p}} = q_{\mathrm{str}}/2\pi r_{\mathrm{cyl}} l$. According to our definition, r_{cyl} is the radius of an equivalent cylinder whose effective charge is equal to that of the double helical molecular structure.

Fig. 2a presents calculations of the electrostatic potential distribution for B-DNA and A-RNA for $r_{\mathrm{p}} = 1$ Å and two different values of $w = 0$ and $w = 1.4$ Å for B-DNA, and $w = 0$ and $w = 3$ Å for A-RNA. As shown in Fig. 2a, the equivalent radius for the case of a B-DNA with $w = 0$ is approximately r_{cyl}=8.8 Å. The surface potential distributions for the various models are

considerably different from one another which arises from the non-uniform charge distribution and the topography of the molecular surface. To further compare the molecular models with charged cylinders, we considered the electrical potential distribution on a plane perpendicular to the plane of the slit containing the axis of the molecule/cylinder (Fig. S10c). We determined the average potential on this plane as a function of radial distance from the molecular axis, ρ (Fig. S10d). Comparing the cylinder model and the molecular structure, we find that, as expected, due to differences in geometry and surface charge distributions, there is indeed a disparity in the average potentials in "near fields" (NF) of the two objects ($\rho \lessapprox 2$ nm). This difference in average potential, however, decays monotonically with distance vanishes in the "bulk" (B) and "far field" (FF) regions ($\rho > 2$ nm). Fig. S10e depicts the contributions of these regions to F_{el} by evaluation of the integral in equation (S30) over subdomains representing NF, bulk (B), and FF regions. In this analysis, a capsule of radius 20 Å measured from the molecular axis, enclosing the molecule/cylinder is considered as the NF region, and a sheet of thickness of 40 Å near the walls represents the FF region. Although the definition of these subdomains is empirical, the analysis qualitatively shows that the contribution of "bulk" region to the free energies, and therefore the effective charge, is not significant in comparison to those of the FF and NF, which in turn we find are comparable in magnitude. Thus the regions in the vicinity of both molecule and walls contribute in approximately equal measure to the overall experimental effect. Note that while in this picture the FF region is designated as the electrostatic far-field of the molecule it may equally well be thought of as the electrostatic near-field of the wall regions. Thus the interaction free energy stems almost entirely from the near-field regions of the interacting objects.

S7.4 The role of probe radius, r_{p}, and its impact on the calculated effective charge, q_{eff}

In the calculation of molecular free energies, the geometric properties of the molecular surface used depend on the choice of radius of the rolling probe. Therefore the probe radius, r_{p}, is another parameter that merits investigation. Free energy calculations for various molecular models show that, generally, the probe radius has a negligible impact on the calculated effective charge as long as the global features of the molecular surface are unaffected by the change of probe size. We examined this effect by considering a larger probe radius of $r_{\mathrm{p}} = 5$ Å. For B-DNA we find that this increase in probe radius (from 1 to 5 Å) increases the magnitude of the effective charge by approximately 2%. Interestingly the impact on the calculated q_{eff} of A-RNA is much larger (≈25%). Figure S10B displays the influence of probe radius on the surface potentials of the two helical species. A comparison reveals that increasing the probe radius only slightly modifies the B-DNA surface by smoothing out small details (such as crevices) of the molecular surface, and therefore, as noted above has a relatively minor impact on the calculated effective charge. Contrary to B-DNA, the A-RNA structure undergoes a significant change, losing its narrow and deep major groove, which effectively reflects the fact that the molecular interior becomes progressively impenetrable to rolling probes of larger radii, as shown in Fig. S10b (top view).

In general, the effective charge values calculated for A-RNA with $r_{\mathrm{p}} < 5$ suggest equivalent cylinder radii values, $r_{\mathrm{cyl}} < r_{\mathrm{c}}$, which turn out to be much smaller than the experimentally measured $r_{0,\mathrm{A}}$ value. While there is no requirement *a priori* for the radius of the equivalent cylinder to be comparable to that of the outer helical envelope, the indication of $r_{\mathrm{cyl}} < r_{\mathrm{c}}$ from these computational molecular models may in fact reflect an unphysical result for the following reasons. One consideration is likely to be the continuum character of the PB equation which also treats ions as point-like particles. It is known that at high magnitudes of electrical potential the finite size of ions causes the point-ion assumption to break down. This is because the close packing condition - which specifies an upper bound on ionic concentrations - is violated. The maximum magnitude of surface potentials for A-RNA models under our experimental conditions ($|\psi|_{\max} \approx 11.5$) indicates local concentrations of

around ≈100 M >> 9 M which represents the close packing concentration given by $(2a_{\mathrm{H}})^{-3}$ for Na^{+} ions ($a_{\mathrm{H}} \approx 2.8$ Å). This strongly suggests that a departure from the PB regime is highly likely for an A-RNA molecule. Another feature distinguishing the A-RNA structure from that of B-DNA is the deep major groove, which creates the impression of a "hollow spine" along the molecular axis when viewed from the top (Fig. 2a and Fig. S10b). The point-ion PB model predicts high counterion concentrations in the major groove under our experimental conditions, and yet again it is not clear that the PB calculations point to physically plausible local concentrations under experimental conditions where ionic species have finite sizes and the maximum attainable concentrations would be bounded by an upper limit[29]. We point that within the PB model the experimental measurements require the presence of a hydration sheath of thickness w, whose main structural effect is to entirely occlude ions from within an outer molecular surface envelope.

S7.5 Accounting for finite ion size in the point-ion PB model

We now discuss the role of finite ion size in the molecular electrostatics problem. We focus in particular on explicit corrections to the PB model that account for finite ion size. In particular we show that the simple PB model is in fact able to account for finite ion size in a direct fashion involving an increase in the radius of the corresponding equivalent cylinder.

Using a lattice gas approach, Borukhov *et al.* proposed a modified PB (MPB) model which uses the close packing limit to define an upper bound for local ionic concentrations[30]. Use of this MPB model and the related free energy functionals in our calculations gives results that are effectively identical to the point-ion model for B-DNA as expected. For A-RNA however, the MPB model yields larger calculated r_{cyl} values, in good agreement with the experimental measurements. This is expected on the grounds that the average surface electrical potentials for A-RNA tend to be higher than for B-DNA. However, we point out that the close packing limit is a purely geometric condition which prohibits unphysical overlapping of ions in a lattice model. This particular MPB model does not take into account short-range interactions between ions and surfaces due to which steric effects can manifest at ion concentrations much lower than the close packing limit, as demonstrated in more recently described MPB models such as the Poisson-Helmholtz-Boltzmann (PHB) model[11]. The ability to capture the role of finite ion size in the limit of low ion densities could be important in modeling B-DNA self consistently and may be possible using the PHB model. Thus while the simple PB model does permit us to effectively deduce aspects of the interfacial structural detail of the double helix required to capture the experimental measurement (see below), a more general model incorporating finite ion size will enhance the robustness of the parameter values and molecular structural detail inferred in this work.

Despite the fact that the simple point-ion PB model does not explicitly incorporate finite ion size, we have found that the point-ion approach can indeed lend itself to accounting for finite ion size at the molecule/electrolyte interface. The basis of the underlying argument is illustrated in Fig. S10f. Essentially, the existence of a hydration layer around the molecule and finite size of counterions implies the presence of a low-dielectric "shell" region in the vicinity of the molecular structure, whose thickness may be expected to be well approximated by the radius of a hydrated counterion. For illustrative purposes we compare two cylinder models of the same length, carrying identical amounts of total charge. The first model (model (a)) has the charge distributed on the "interior" molecular ("vdW") surface but is surrounded by a dielectric shell of dielectric constant ε_{s}, representing the charge free (c=0), ion-excluded zone (Fig. S10f model (a)). The exterior surface of this shell region may be regarded as equivalent to an "ion accessible surface" (IAS) beyond which the point-ion description should hold provided the local electrical potential $\psi < 9$ for $c \approx 1$ mM. This condition may be expected to apply for B-DNA in our experiments. The second model (model (b)) considers a simpler hollow object consisting of a single cylindrical surface of radius equal to the IAS in model (a). Model (b) ignores interior dielectric detail entirely. Figure S10F(ii) compares the calculated q_{eff}

values for the two models by plotting the ratio of effective charges for the two cases. We find that neglecting the dielectric region and distributing the same amount of charge on the outer (IAS) surface in model (b) has negligible impact on the effective charge. For example, for $\varepsilon_{\mathrm{s}} = 2$ and $t = 3$ Å we expect the discrepancy in q_{eff} for the two models to lie at around 0.7% for a 30 bp fragment and 0.3% for 60 bp fragment of B-DNA (Fig. S10f). These percentage differences are of the the same order as the broadening of the experimental data due to statistical uncertainty and may be regarded as effectively negligible for our purposes. Furthermore we find that the similarity in q_{eff} between the dielectric shell and simple cylinder models holds for a range of shell thicknesses, t, and shell dielectric constants, ε_{s}, relevant to the problem (Fig. S10f). We therefore expect the effective cylindrical radius parameter, r_{cyl}, to successfully capture the effect of finite ion size in the determination of q_{eff}. Thus, based on the above results, we have neglected the dielectric shell region, that could more accurately reflect an ion excluded zone, for all q_{eff} calculations for molecular structures and cylinders. Significantly, the above result provides a physical basis for why we observe a dependence of the measured effective cylinder radius, r_{m}, of the double helix on the radius of the cationic species in solution (Fig. 4a).

List of important variables and parameters

Symbol	Variable or parameter name
b	Helical rise per basepair
b_{m}	Experimentally measured helical rise per basepair
c	Salt concentration
d	Depth of the pocket structure
D	Diffusion coefficient
e	Elementary unit charge
f_{A}	Additive correction factor
f_{H}	Hydrodynamic correction factor
f_{M}	Multiplicative correction factor
F_{el}	Electrostatic free energy
ΔF_{el}	Electrostatic contribution to the total free energy
ΔF_{rot}	Free energy contribution to W arising from rotational motion of a rod-shaped molecule
$\Delta F_{\mathrm{trans}}$	Free energy contribution to W arising from position fluctuations of the center of mass
$2h$	Measured slit height
κ^{-1}	Debye length
k_{B}	Boltzmann's constant
l	Contour length of the nucleic acid molecule
n	Number of basepairs
μ	Solvent viscosity
T	Temperature
t_{cycle}	Cycle time in fluorescence imaging
t_{esc}	Average escape time of the molecule
t_{exp}	Exposure time in fluorescence imaging
t_{lag}	Lag time in fluorescence imaging
t_{r}	Position relaxation time of the trapped molecule
r	Helical radius
r_{m}	Measured radius of the nucleic acid molecule
r_{H}	Hydrodynamic radius measured using FCS
q_{dye}	Effective charge of a single dye attached to each 5' terminus of a nucleic acid
q_{eff}	Calculated effective electrical charge
q_{m}	Measured electrical charge in a real experiment
q_{NA}	Number of fully ionized backbone phosphate groups
q_{str}	Net structural charge
W	Depth of the trap or potential well
x_{e}	Error on parameter x
x_{in}	Input test value for parameter x used in a simulation
x_{out}	Output value for parameter x obtained in a simulation with input value x_{in}
x_{t}	True value for parameter x
ϕ_{m}	Electrical potential at the slit mid-plane
ϕ_{s}	Electrical potential at the surface of the slit wall

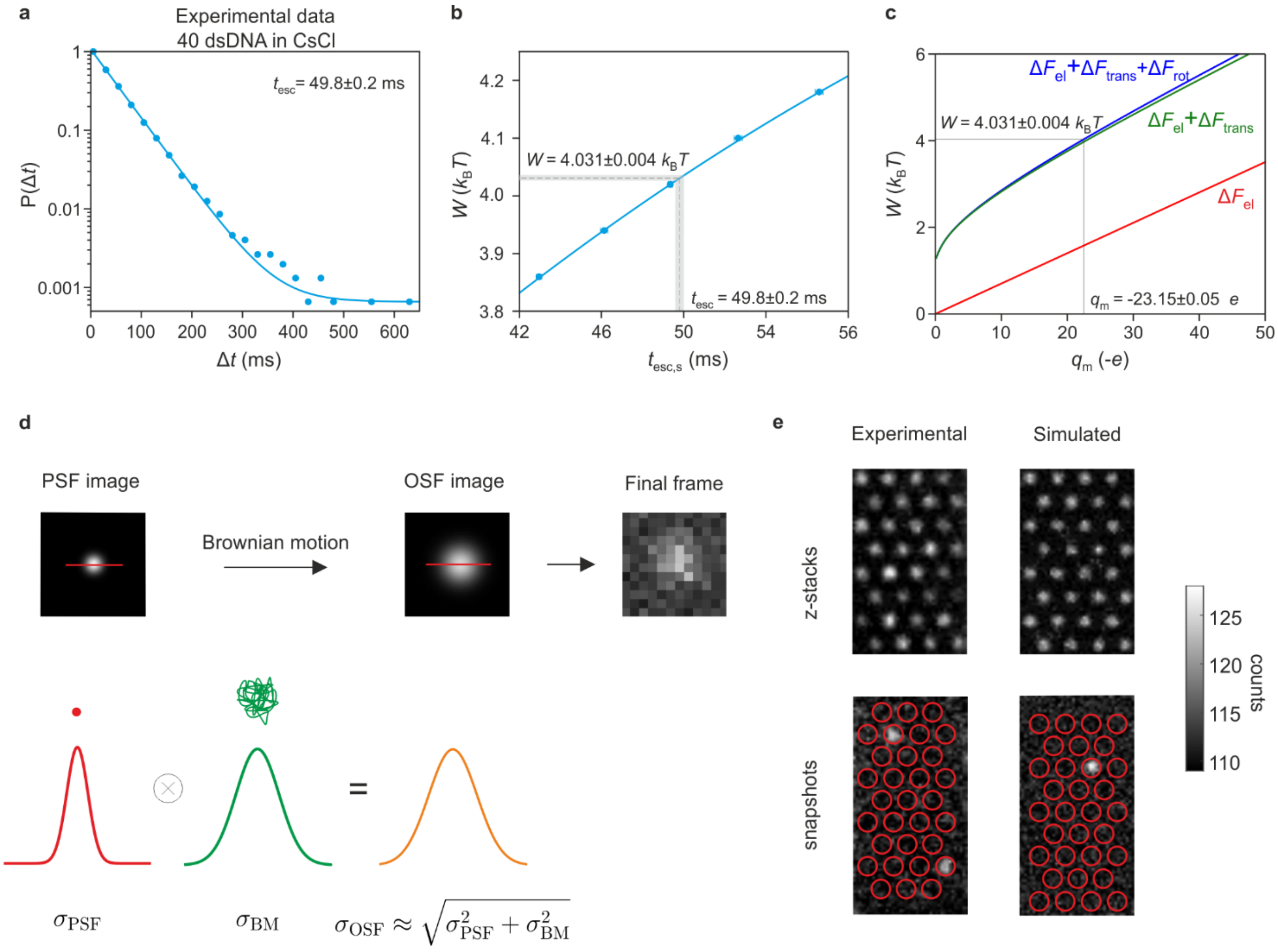

Figure S1. Converting measured escape times, t_{esc}, to measured molecular effective charge, q_m. (a) A sample histogram of measured escape times fitted with a single exponential, $P(\Delta t) \propto \exp(-\Delta t/t_{esc})/t_{esc}$, for 40 dsDNA measured in 1.21 mM CsCl, 1 mM TRIS, pH=8.9. **(b)** Brownian Dynamics (BD) simulations of molecular motion in a free energy landscape yield a relationship for potential well depth W vs. average escape time, $t_{esc,s}$, fitted with a second degree polynomial. Values for $t_{esc,s}$ were obtained from image analysis of simulated movies (procedure described below). The t_{esc} value from experimental measurements was converted to a measurement of W using this fit relation. **(c)** Plot of W as a function of q_m, for $\phi_s = -2.8\ k_B T/e$, displaying the various contributions to W, namely ΔF_{el}, ΔF_{trans} and ΔF_{rot}. **(d)** A series of images depicting from left to right: the optical point spread function (PSF) for a single stationary molecule, a representative optical image for a molecule exhibiting thermal motion, which we call the object spread function (OSF) image, and a final pixelated image superimposing the OSF on the background intensity, including shot noise, mimicking the actual experiment. The OSF image is a convolution of the PSF image of a stationary emitter with a probability density representative of the molecule's spatial fluctuations due to Brownian motion within a time period $t_{exp} = 5$ ms, obtained from a BD simulation, as described in Ref. 2. **(e)** Top: Maximum intensity projection of experimental (left) and simulated (right) widefield fluorescence images of an array of 32 traps. Bottom: typical experimental (left) and simulated (right) snapshot camera images. Locations of traps are indicated by red circles. The simulation contains a single molecule sampling the array, while the experiment can involve more than one molecule simultaneously in the same field of view. Photobleaching is not expected to pose an issue as established in previous work[1].

a

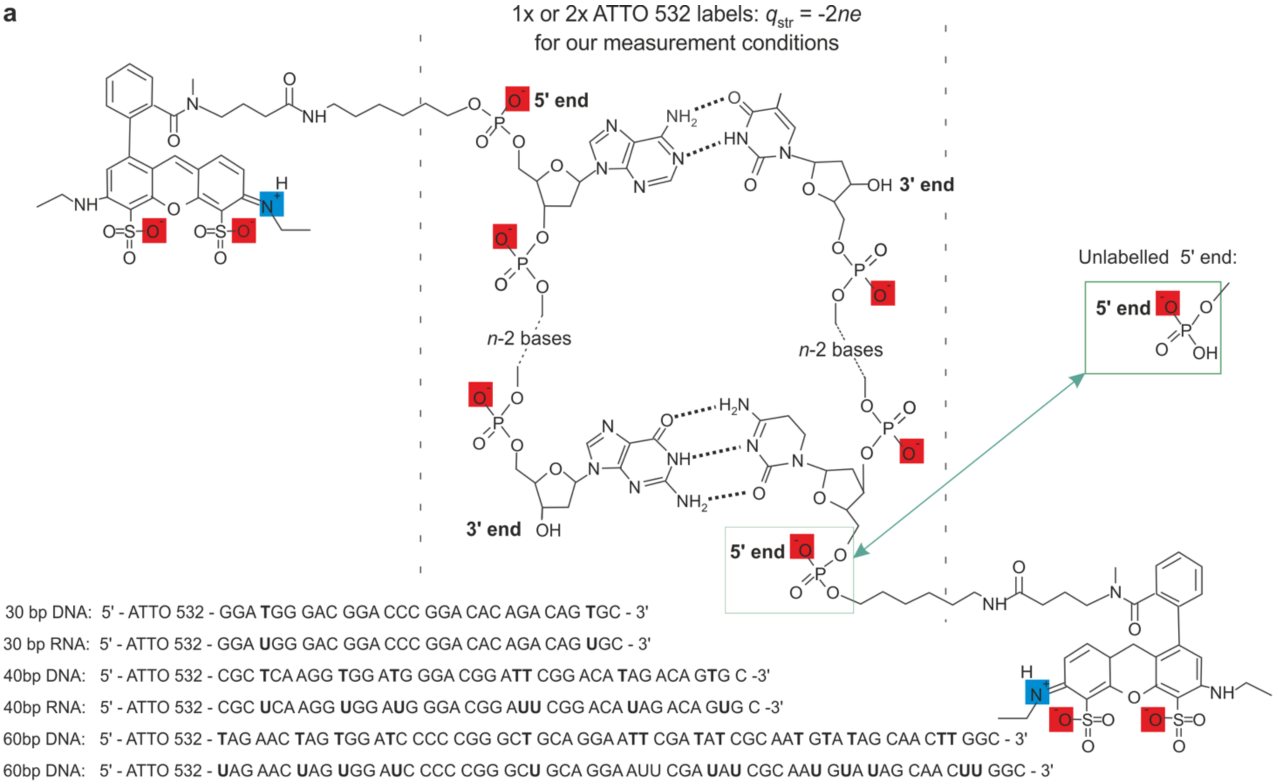

b

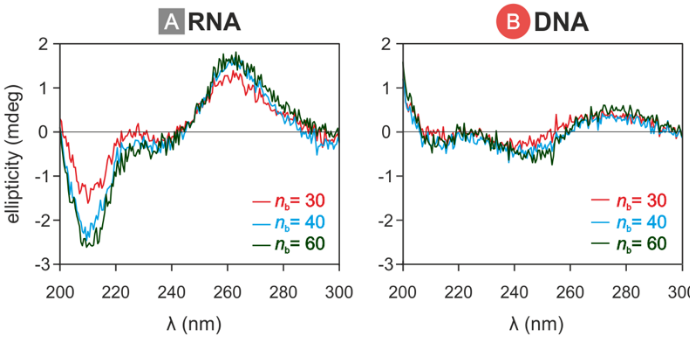

Nucleic acid	λ_{min} (-) (nm)	$\lambda_{crossover}$ (nm)	λ_{max} (+) (nm)
A-RNA	210	243	263
B-DNA	247	260	275

c

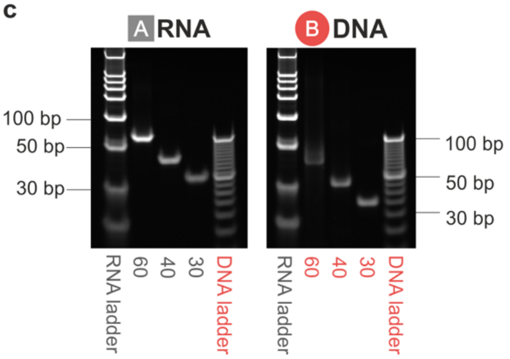

d

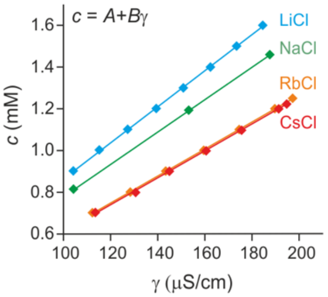

Salt	*A*	*B*	a_m, Å	a_H, Å
LiCl	-0.00397	0.00869	3.14	3.58
NaCl	-0.00185	0.00781	2.40	2.76
RbCl	-0.03148	0.00651	1.64	1.78
CsCl	-0.04213	0.00652	1.65	1.79

Figure S2. Characterization of B-DNA and A-RNA samples. (a) Chemical structure of dsDNA with two ATTO 532 modifications on both 5'-ends. Charge carrying atoms, ionized under our conditions, are highlighted in blue (positive) and red (negative). In singly labeled nucleic acid fragments only one 5'-phosphate end carries the dye modification; the other remains underivatized, as shown. A ds nucleic acid, *n* basepairs in length, labeled with either one or two ATTO 532 dyes on

its 5'-ends respectively, carries a structural charge $q_{\mathrm{str}} = q_{\mathrm{NA}} = -2ne$. This value is the charge of the nucleic acid alone and excludes the contribution of the dye. dsDNA and dsRNA sequences used in the measurements have effectively identical sequences with uracil (U) replacing thymine (T) in dsRNA (bottom). **(b)** CD spectra of 30 bp, 40 bp, 60 bp dsDNA and dsRNA fragments measured in an electrolyte solution containing 1 mM NaCl, 1-1.3 mM Tris. The table presents average peak and crossover bands for dsDNA and dsRNA structures, corresponding to B- and A-helices respectively. **(c)** 20% native polyacrylamide gel for A-RNA (left) and B-DNA (right) fragments stained with GelRed dye. dsRNA gel (left): Lane 1 – dsRNA ladder, 2 – 60 bp dsRNA, 3 – 40 bp dsRNA, 4 – 30 bp dsRNA, 5 – 5 bp dsDNA ladder. dsDNA gel (right): Lane 1 – dsRNA ladder, 2 – 60 bp dsDNA, 3 – 40 bp dsDNA, 4 – 30 bp dsDNA, 5 – 5 bp dsDNA ladder. All fragments display slightly lower mobilities than the corresponding fragments in the ladders, likely due to the presence of the charged fluorescent dye labels. **(d)** Converting measured solution conductivity, γ (in $\mu\mathrm{S/cm}$) to salt concentration, c (in mM). Solid lines denote the linear calibration function $c = A + B\gamma$ used to convert γ to c. Fit A and B coefficients are individual for every salt: CsCl (red), RbCl (orange), NaCl (green), and LiCl (blue). The table lists values for hydrated cationic radii, a_{m}, that we obtained using the equation (S27), $a_{\mathrm{Cl^-}}$ =1.81 Å and the fit relation. Values of a_{H} from Ref. 18 are also listed.

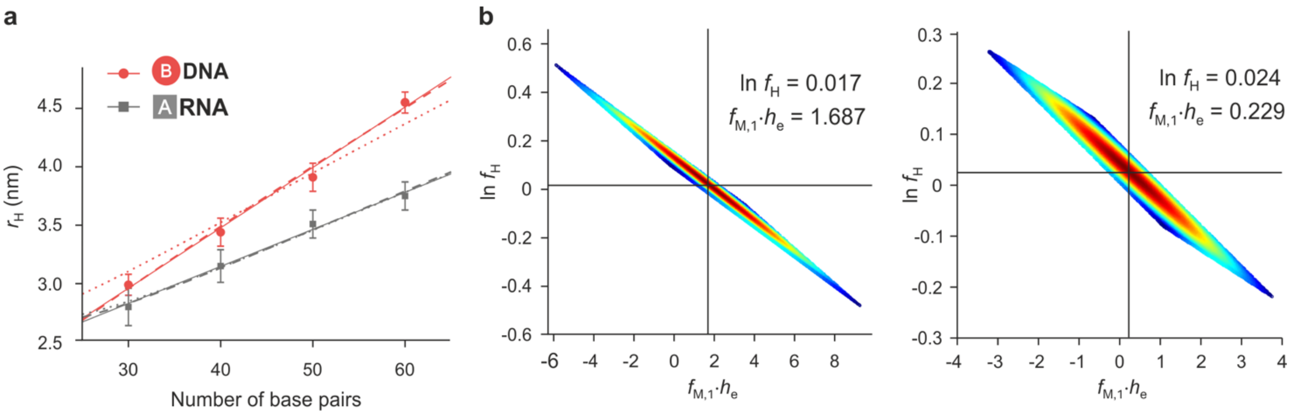

Number of base pairs	r_H(B-DNA), nm	r_H(A-RNA), nm
30	2.96±0.21	2.83±0.17
40	3.48±0.24	3.15±0.19
50	3.99±0.26	3.46±0.22
60	4.51±0.29	3.78±0.24

$r_H = r_{H,t} \cdot f_H$

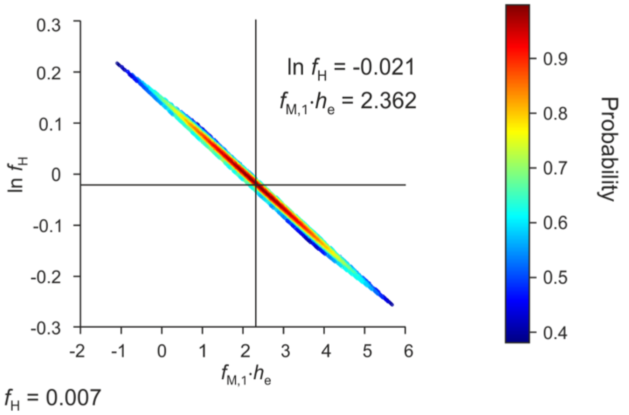

Average ln f_H = 0.007

Figure S3. Measuring the hydrodynamic uncertainty, f_H. (a) Hydrodynamic radii, r_H of dsDNA (red circles) and dsRNA (grey squares) for fragments of length n basepairs measured using FCS. Independent linear fits of r_H vs n were performed for both dsDNA (red solid line) and dsRNA (grey solid line) fragments. r_H values for each fragment from the linear fit relations were used in BD simulations and are listed in the table below. Furthermore, the measured r_H vs n data were also fitted with an equation of the form $r_H = l \,/\, 2[\ln(\,l/2r) + v]$ as described in the text. Performing the fitting procedure with three fit parameters, b, r v, we obtained: $b = 4.4 \pm 8.5$ Å, $r = 9.3 \pm 18516$ Å, $v = 0.26 \pm 2001$ for B-DNA (red dashed line), and $b = 2.6 \pm 1.9$ Å, $r = 10.4 \pm 4042$ Å, $v = 0.06 \pm 389$ for A-RNA (grey dashed line). Dotted lines (red for B-DNA, grey for A-RNA) illustrate the fits performed with b values fixed to the nominal crystallographic values, 3.4 Å for B-DNA and 2.6 Å for A-RNA. In this case we obtained: $r = 9.8 \pm 1255$ Å, $v = -0.004 \pm 127$ for B-DNA, and $r = 9.7 \pm 315$ Å, $v = -0.02 \pm 32$ for A-RNA. **(b)** Measuring the hydrodynamic correction factor, f_H. Solving the system of two linear equations of the form equation (S18) graphically, we first obtained the space of possible $f_{M,1} \cdot h_e$ and $\ln f_H$ values, and then determined the weighted mean values (black solid lines) for both unknowns. Three sets of measurements revealed average value of $\ln f_H = 0.007 \pm 0.025$ or $f_H = 1.007 \pm 0.025$.

a

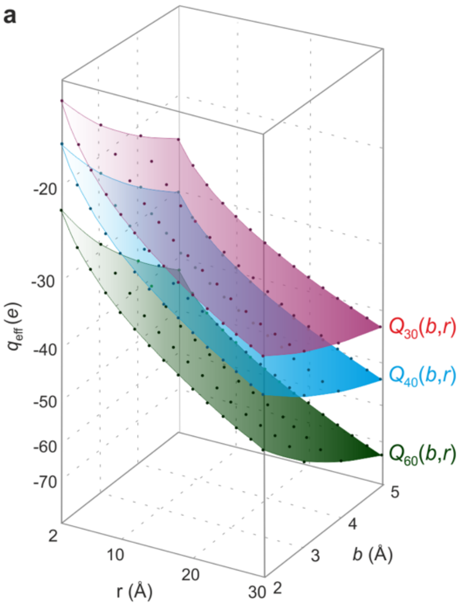

$$Q_n(b,r) = a_1 + a_2(b^{\alpha} + a_3)(r^{\beta} + a_4)$$

b and r in nm

c (mM)	n	α	β	a_1	a_2	a_3	a_4
1.00	30	0.522	0.662	824.291	-0.505	21.161	76.477
	40	0.589	0.696	232.284	-2.439	4.561	20.261
	60	0.656	0.726	93.928	-8.798	1.263	7.834
1.10	30	0.560	0.663	374.804	-1.072	9.929	34.721
	40	0.592	0.706	196.097	-2.873	3.819	17.248
	60	0.645	0.739	84.476	-9.679	1.110	7.174
1.20	30	0.524	0.688	617.208	-0.681	15.520	57.753
	40	0.584	0.722	172.824	-3.236	3.318	15.433
	60	0.654	0.753	83.676	-9.849	1.094	7.098
1.25	30	0.549	0.694	599.165	-0.692	15.288	56.090
	40	0.580	0.725	162.044	-3.470	3.070	14.509
	60	0.642	0.755	79.250	-10.396	1.017	6.758
1.30	30	0.521	0.701	319.224	-1.277	7.987	30.615
	40	0.576	0.728	154.606	-3.657	2.908	13.813
	60	0.642	0.762	77.048	-10.664	0.981	6.614
1.35	30	0.546	0.705	292.329	-1.367	7.444	28.132
	40	0.583	0.733	137.571	-4.019	2.605	12.453
	60	0.644	0.767	76.696	-10.780	0.973	6.563
1.50	30	0.544	0.715	298.719	-1.373	7.474	28.474
	40	0.550	0.756	143.345	-4.005	2.569	13.039
	60	0.628	0.782	71.459	-11.597	0.875	6.173

b

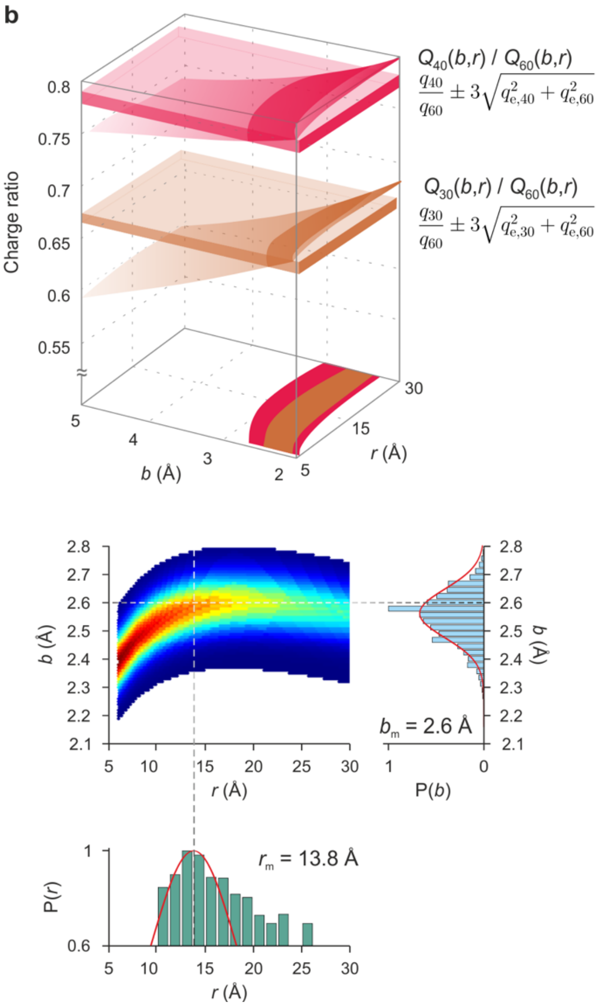

c

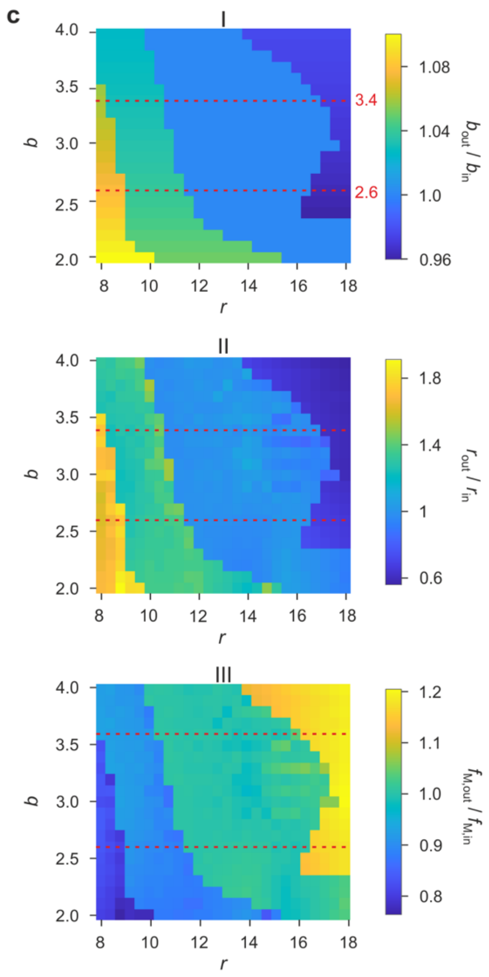

Figure S4. Principle behind the measurement of rise per base pair, b and radius, r, of the double helix, in a real experiment (i.e., $f_M \neq 1$). (a) 2D surfaces for the effective charge values obtained by fitting the calculated effective charge values (shown with points), $q_{\mathrm{eff},n}(b,r)$, with a power-law function. The table lists the fit values of all parameters in the power-law fit equations for $n = 30$, 40

and 60 basepairs determined for salt concentrations ranging from 1 to 1.5 mM. **(b)** Measured values for effective charge ratios, e.g. $\frac{q_{30}}{q_{60}} \pm 3\sqrt{q_{e,30}^2 + q_{e,60}^2}$ and $\frac{q_{40}}{q_{60}} \pm 3\sqrt{q_{e,40}^2 + q_{e,60}^2}$, superimposed as planes of finite thickness in charge on the corresponding 2D surfaces, e.g. $Q_{30}\,(b,r)/Q_{60}\,(b,r)$ and $Q_{40}\,(b,r)/Q_{60}\,(b,r)$. This yields two domains of possible solutions in b and r. Intersection of these domains yields a probability weighted manifold of solutions from which measured values of helical rise per base pair, b_{m} and radius, r_{m}, are extracted. The lower panel illustrates the results for one measurement of A-RNA in CsCl measurements. **(c)** Results of a simulation based study of the overall accuracy in the determination of r (panel I), b (panel II), and f_{M} (panel III) for various combinations of input parameters b_{in} and r_{in}. The level of accuracy expected is plotted as a ratio $x_{\mathrm{out}}/x_{\mathrm{in}}$, where x denotes either b, r or f_{M}. Dotted red lines indicate $b = 3.4$ Å or 2.6 Å and are provided as visual guides. Maximum accuracy in determination of r, b and f_{M} is obtained within the ranges of r_{in}= 10.5-17.0 Å for $b = 3.4$ Å and $r_{\mathrm{in}} = 11.3$-16.5 Å for $b = 2.6$ Å.

a

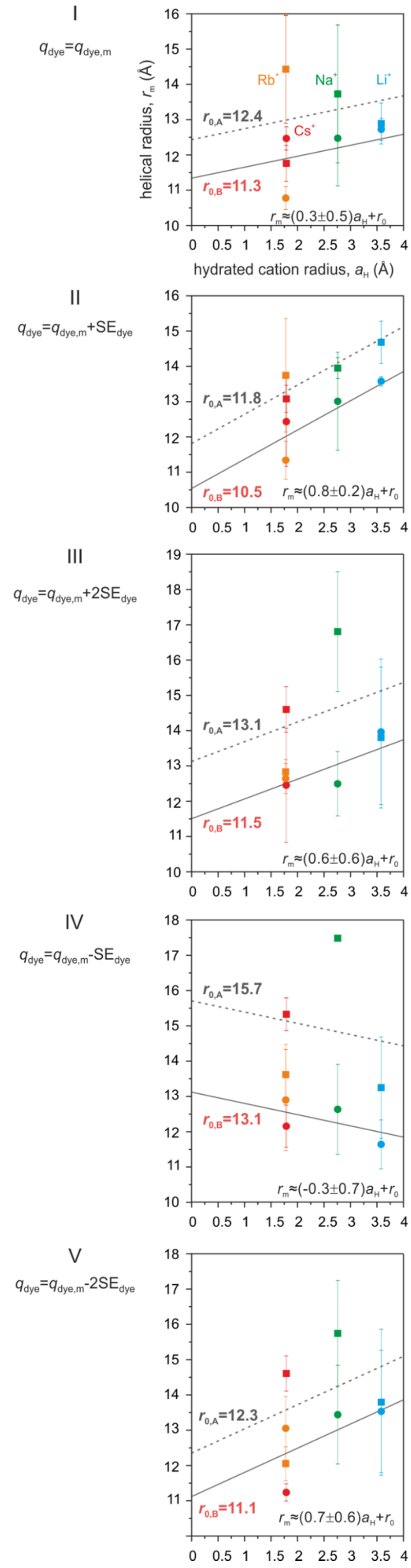

b

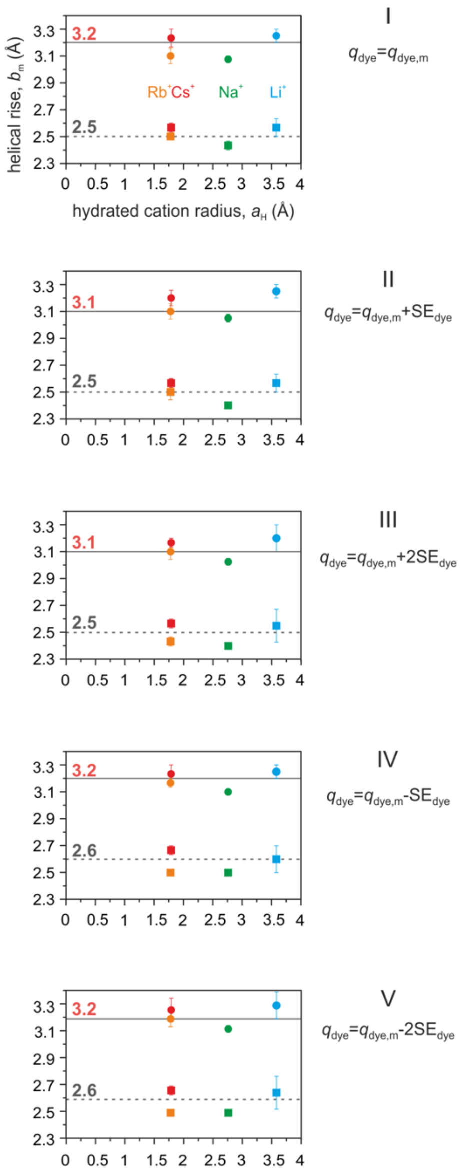

c

	Used q_{dye}	$r_{0,B}$, Å	$r_{0,A}$, Å	slope
I	$q_{dye}=q_{dye,m}$	11.3±1.4	12.4±1.4	0.3±0.5
II	$q_{dye}=q_{dye,m}+SE_{dye}$	10.5±0.6	11.8±0.6	0.8±0.2
III	$q_{dye}=q_{dye,m}+2SE_{dye}$	11.5±1.7	13.1±1.7	0.6±0.6
IV	$q_{dye}=q_{dye,m}-SE_{dye}$	13.1±1.9	15.7±1.9	-0.3±0.7
V	$q_{dye}=q_{dye,m}-2SE_{dye}$	11.1±1.7	12.3±1.7	0.7±0.6

Figure S5. Examining the influence of small perturbations of the value of q_{dye} on the inferred average values for helical radius, r_{m}, and rise per base pair, b_{m}. The analysis is performed for $\ln f_{\mathrm{H}} = 0.007$ and corresponding measured q_{dye} values: $q_{\mathrm{dye,DNA}} = -0.46\,e$ with $\mathrm{SE}_{\mathrm{dye,DNA}} =$

$0.04\,e$, $q_{\mathrm{dye,RNA}} = -0.44\,e$ with $\mathrm{SE}_{\mathrm{dye,RNA}} = 0.05\,e$. The analysis procedure is repeated by introducing systematic offsets on the average q_{dye} value given by $\pm\mathrm{SE}_{\mathrm{dye}}$ and $\pm2\mathrm{SE}_{\mathrm{dye}}$. **(a)** Influence of the value of q_{dye} on the measured average r_{m} values for B-DNA (circles) and A-RNA (squares). The r_{m} vs a_{H} data were fitted with a linear expression of the form $r_{\mathrm{m}} = a_{\mathrm{H}} \cdot k + r_0$ with a shared value of slope, k, for both helical forms (solid line for B-DNA and dashed line for A-RNA). **(b)** Influence of the value of q_{dye} on the measured average b_{m} values for B-DNA (circles) and A-RNA (squares). Black lines (solid for B-DNA and dashed for A-RNA) denote the average values for b_{m} over all the measurements for a given q_{dye} value. **(c)** List of fitted values for $r_{0,\mathrm{A}}$, $r_{0,\mathrm{B}}$ and slope, k, obtained by linear extrapolation of the measured r_{m} vs a_{H} data to a_{H} =0. The result corresponding to $q_{\mathrm{dye}} = q_{\mathrm{dye,m}} + \mathrm{SE}_{\mathrm{dye}}$ (highlighted in the table) is used for all the further analysis and discussions in the study.

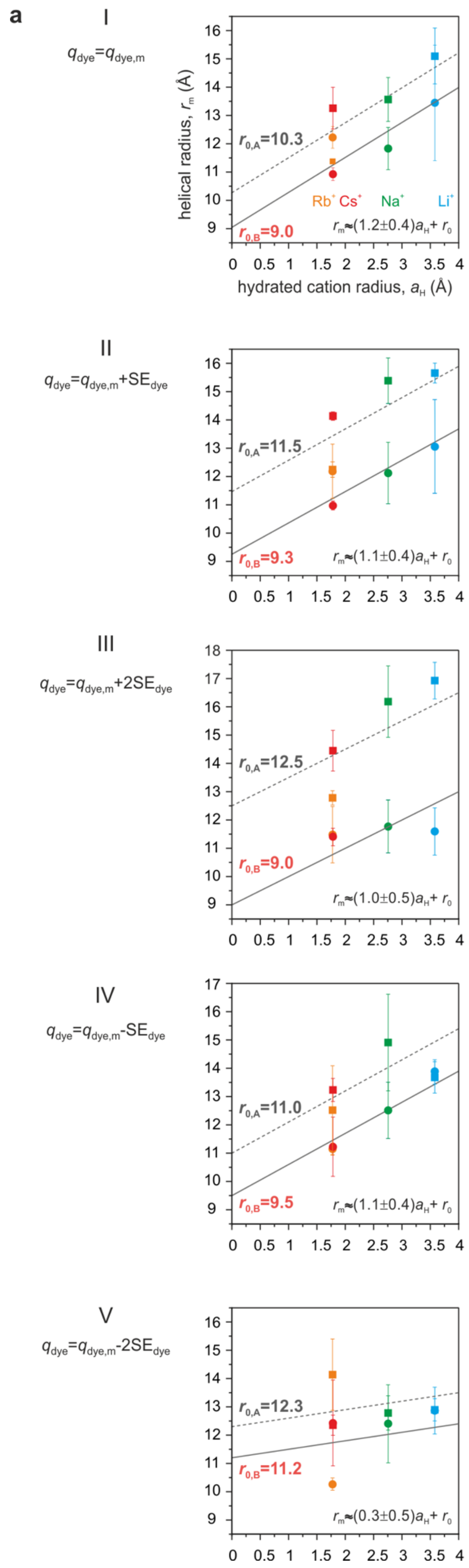

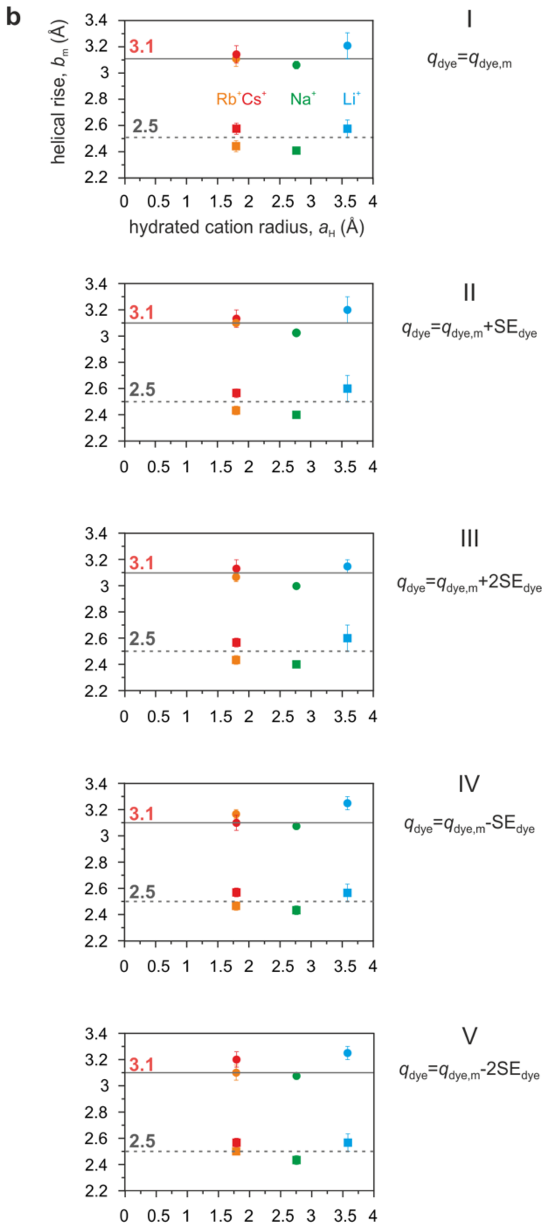

c

	Used q_{dye}	$r_{0,B}$, Å	$r_{0,A}$, Å	slope
I	q_{dye}=$q_{dye,m}$	9.0±1.1	10.3±1.1	1.2±0.4
II	q_{dye}=$q_{dye,m}$+SE$_{dye}$	9.3±1.1	11.5±1.1	1.1±0.4
III	q_{dye}=$q_{dye,m}$+2SE$_{dye}$	9.0±1.3	12.5±1.3	1.0±0.5
IV	q_{dye}=$q_{dye,m}$-SE$_{dye}$	9.5±1.0	11.0±1.0	1.1±0.4
V	q_{dye}=$q_{dye,m}$-2SE$_{dye}$	11.2±1.3	12.3±1.3	0.3±0.5

Figure S6. Examining the influence of small perturbations of the value of q_{dye} on the inferred average values for helical radius, r_{m}, and rise per base pair, b_{m}. The analysis is performed for $\ln f_{\mathrm{H}} = 0.013$ and corresponding measured q_{dye} values: $q_{\mathrm{dye,DNA}} = -0.45\,e$ with $\mathrm{SE}_{\mathrm{dye,DNA}} =$

$0.03\,e$, $q_{\mathrm{dye,RNA}} = -0.42\,e$ with $\mathrm{SE}_{\mathrm{dye,RNA}} = 0.03\,e$. The analysis procedure is repeated by introducing systematic offsets on the average q_{dye} value given by $\pm\mathrm{SE}_{\mathrm{dye}}$ and $\pm 2\mathrm{SE}_{\mathrm{dye}}$. **(a)** Influence of the value of q_{dye} on the measured average r_{m} values for B-DNA (circles) and A-RNA (squares). The r_{m} vs a_{H} data were fitted with a linear expression of the form $r_{\mathrm{m}} = a_{\mathrm{H}} \cdot k + r_0$ with a shared value of slope, k, for both helical forms (solid line for B-DNA and dashed line for A-RNA).
(b) Influence of the value of q_{dye} on the measured average b_{m} values for B-DNA (circles) and A-RNA (squares). Black lines (solid for B-DNA and dashed for A-RNA) denote the average values for b_{m} over all the measurements for a given q_{dye} value. (**c**) List of fitted values for $r_{0,\mathrm{A}}$, $r_{0,\mathrm{B}}$ and slope, k, obtained by linear extrapolation of the measured r_{m} vs a_{H} data to $a_{\mathrm{H}} = 0$.

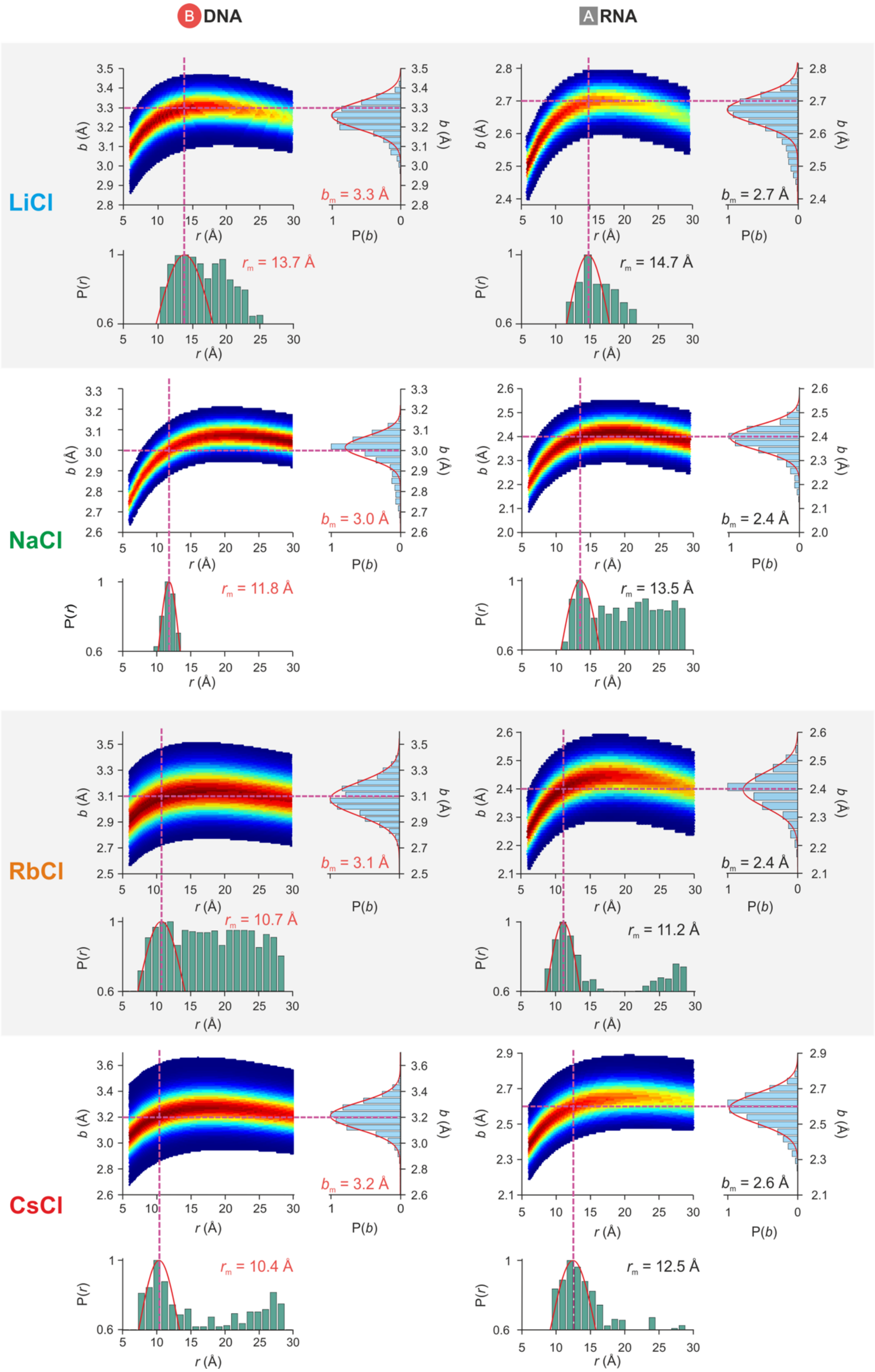

Figure S7. Example measured *b*-*r* probability manifolds for B-DNA (left) and A-RNA (right) for experiments performed in solutions containing LiCl, NaCl, RbCl or CsCl (from top to bottom). Experimental details on these data sets are denoted in boldface in Table S1.

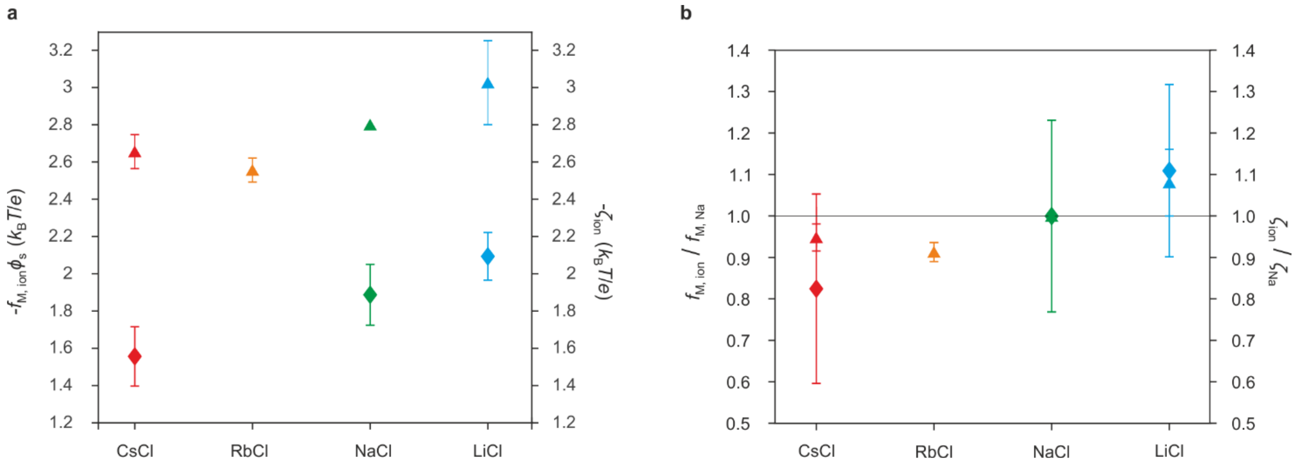

Figure S8. The influence of ionic species on the value of the multiplicative correction factor, f_M. (a) Comparison of measured $f_M\phi_s$ values (triangles), reflecting the true effective surface potential, $\phi_{s,t}$, in our experiments, with zeta (ζ) potential values (diamonds) for CsCl (red), RbCl (orange), NaCl (green) and LiCl (blue) aqueous solutions obtained from Ref. 15. **(b)** Comparison of measured $f_{M,ion}/f_{M,Na}$ values (triangles) with the values for ζ_{ion}/ζ_{Na} (diamonds) for CsCl (red), RbCl (orange), NaCl (greean) and LiCl (blue) aqueos solutions reveal good mutual agreement in the trends.

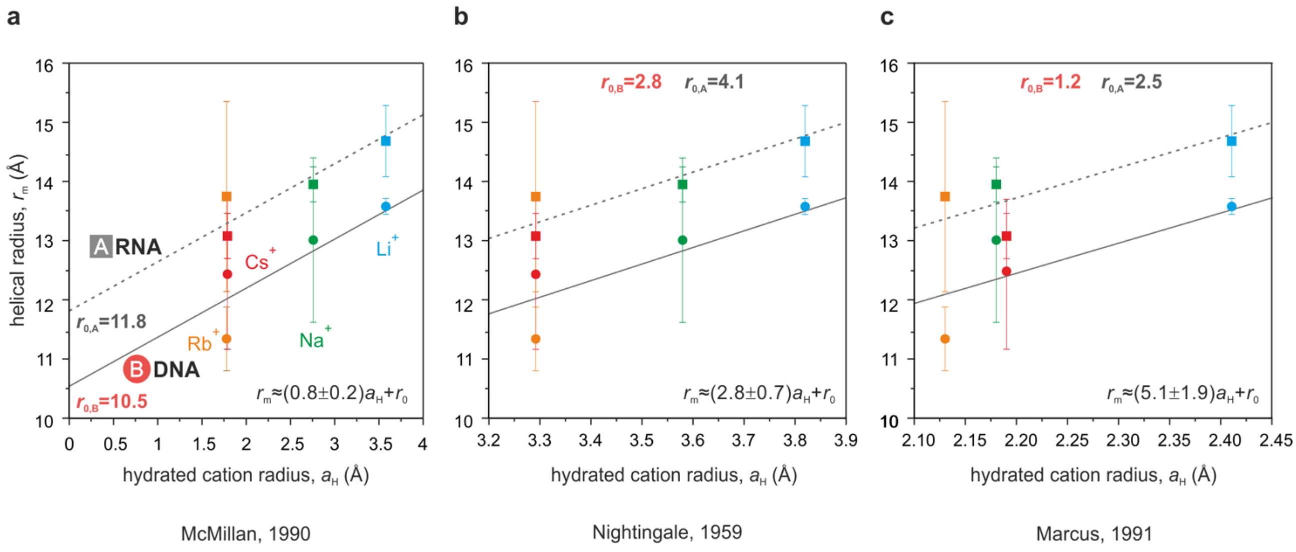

Figure S9. Dependence of the inferred r_m vs a_H relationship on the assumed values of the hydrated cationic radii, a_H. Values for a_H are taken from various sources: **(a)** Ref. 18. **(b)** Ref. 19. **(c)** Ref. 20

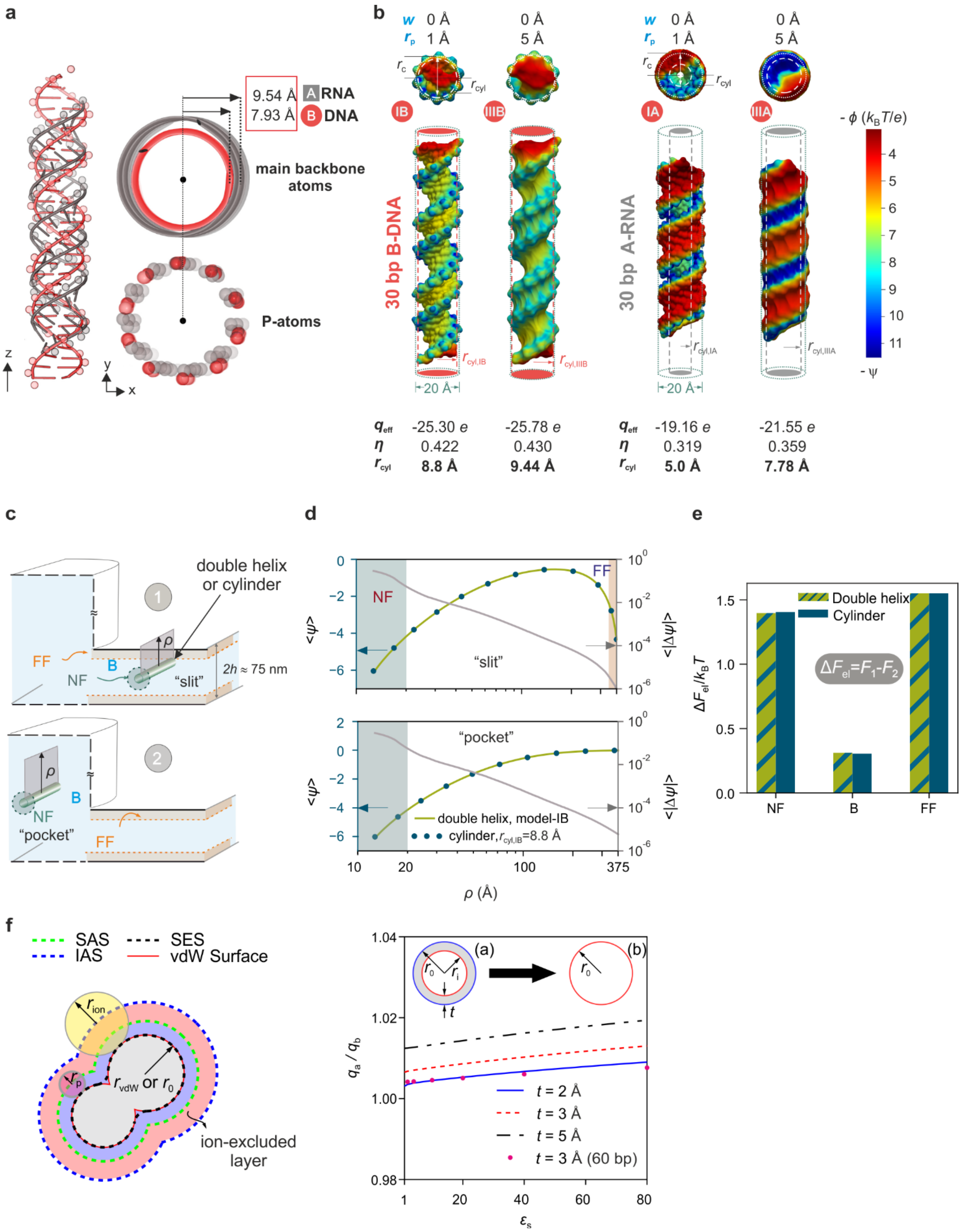

Figure S10. (a) Model structures of A-RNA (grey) and B-DNA (red) generated with the 3DNA platform[17] and visualized with Protein Imager[18] (left) and nglview[19] (right). Side view of the superimposed helices (left) and axial (top)-view down the principle axis of the model structures (right). 'Main backbone' atoms presented in ribbon form (top right) and phosphorous atoms as

spheres (bottom right). Mean backbone atom radii for A-RNA and B-DNA are 9.54 Å and 7.93 Å respectively, while the P atoms suggest a comparable average axial distance in both cases. **(b)** Impact of probe size, r_{p}, on the calculated effective charge, q_{eff}. For B-DNA, a larger probe smooths out the corrugations of the molecular surface but preserves its global structure resulting in a small change in the calculated effective charge (<2%). For A-RNA, an increase in probe radius can lead to the loss of the major groove which affects the effective charge substantially (>10%). **(c)** Analysis of calculated electrostatic free energies in a single electrostatic fluidic trap where state (1) represents the molecule in the slit and state (2) depicts a trapped molecule. NF denotes the electrostatic "near-field" in the vicinity of the molecule while FF defines a thin layer near the walls. The plane containing the axis of the molecule and perpendicular to the wall is used to calculate the average potentials in panel **d**. **(d)** Variations of the average potential, $\langle\psi\rangle$, vs axial distance, ρ, for a B-DNA (model-IB) and its corresponding cylindrical model (r_{cyl}=8.8 Å). The difference in average potential, $\langle|\Delta\psi|\rangle$, monotonically decreases with distance leading to almost identical average potential distributions for B-DNA and the cylinder in the "bulk" and in the "far-field" (ρ >2 nm). **(e)** Contributions of different regions to the total free energy difference for B-DNA (model-IB) and its equivalent cylindrical model ($r_{\mathrm{cyl}} = 8.8$ Å). The total free energy differences, and therefore magnitude of the effective charges, are mostly determined by the contributions from the NF and FF regions which make roughly equal contributions. **(f)** Left: Schematic representation of molecular surfaces. The grey region denotes the interior of the molecule, while the van der Waals surface (vdWS - red lines) and ion accessible surface (IAS - blue lines) display low dielectric regions inaccessible to water molecules and ions, respectively. The solvent accessible surface (SAS - green line) denotes the surface of closest approach of water molecules to the vdWS. The charge distribution outside the IAS is governed by the Boltzmann distribution. Right: Influence of a dielectric shell surrounding a charged cylinder on the calculated effective charge. We compare a cylinder of radius r_{i} =10 Å shielded by a dielectric layer of thickness, t, with a uniform dielectric constant, ε_{s}, yielding a calculated effective charge q_{a} (model (a)), with a hollow cylinder of radius, $r_0 = r_{\mathrm{i}} + t$, with no dielectric layer, described by a calculated effective charge q_{b} (model (b)). Cylinder lengths of 102 Å and 204 Å correspond to those for 30 bp and 60 bp B-DNA, respectively. Calculations were performed for c = 1 mM with the total charge $q_{\mathrm{str}} = -2\,ne$ uniformly distributed on the lateral surface of the cylinders (red) for 30 bp B-DNA (lines) and 60 bp B-DNA (pink circles). The ratio $q_{\mathrm{a}}/q_{\mathrm{b}}$ is of order 1% over a wide range of ε_{s} and t values suggesting that in the electrometry measurement, a hollow cylinder of radius $r_0 = r_{\mathrm{i}} + t$ is an excellent approximation to a charged cylinder of radius r_{i} surrounded by a dielectric shell of thickness t. Panels **(b)**, and **(d)-(f)** are reproduced from Ref. 31, with the permission of AIP Publishing.

	Molecule	Device	Buffer 1 mM Tris +	T (°C)	W (k_BT)	t_{esc} (s)	q_m (-e)	f_M	Structural parameters b_m (Å)	Structural parameters r_m (Å)
LiCl	**30bp DNA**		**1.23 mM LiCl, pH=9.0**	**22.8**	**4.226**	**52.3±0.3**	**25.28±0.07**			
	**40bp DNA**	**2*h*=75.2 nm**	**1.23 mM LiCl, pH=9.1**	**22.9**	**4.655**	**93.9±0.4**	**30.46±0.06**	0.86	3.3	13.7
	**60bp DNA**	***d*=167 nm**	**1.25 mM LiCl, pH=9.1**	**22.8**	**5.374**	**242.5±1.1**	**40.71±0.07**			
	30bp DNA		1.32 mM LiCl, pH=9.0	21.9	3.863	41.0±0.4	25.60±0.16			
	40bp DNA	2*h*=74.7 nm	1.32 mM LiCl, pH=9.0	22.2	4.312	69.2±1.0	31.08±0.22	0.89	3.2	13.4
	60bp DNA	*d*=146 nm	1.29 mM LiCl, pH=9.1	22.0	5.197	209.7±0.6	41.20±0.04			
	60bp DNA (1 dye)		1.29 mM LiCl, pH=9.1	22.0	5.151	203.4±2.1	40.79±0.15			
	**30bp RNA**		**1.23 mM LiCl, pH=8.9**	**22.5**	**4.099**	**46.3±0.2**	**23.86±0.04**			
	**40bp RNA**	**2*h*=75.2 nm**	**1.25 mM LiCl, pH=8.9**	**23.2**	**4.408**	**70.4±0.8**	**28.35±0.13**	0.87	2.7	14.7
	**60bp RNA**	***d*=167 nm**	**1.22 mM LiCl, pH=8.9**	**22.7**	**5.316**	**192.5±0.6**	**37.26±0.04**			
	30bp RNA		1.21 mM LiCl, pH=9.0	22.7	4.244	53.7±0.4	24.74±0.08			
	40bp RNA	2*h*=75.2 nm	1.20 mM LiCl, pH=9.1	22.8	4.645	82.6±0.1	29.08±0.02	0.95	2.5	13.6
	60bp RNA	*d*=167 nm	1.20 mM LiCl, pH=9.1	22.8	5.422	210.6±0.9	37.96±0.06			
	60bp RNA (1 dye)		1.20 mM LiCl, pH=9.0	22.8	5.277	185.1±1.7	37.60±0.13			
	30bp RNA		1.25 mM LiCl, pH=9.0	22.5	4.183	50.2±0.1	25.63±0.03			
	40bp RNA	2*h*=75.2 nm	1.25 mM LiCl, pH=9.0	22.3	4.569	80.0±0.2	30.33±0.03	0.93	2.5	15.7
	60bp RNA	*d*=167 nm	1.24 mM LiCl, pH=9.1	21.9	5.367	207.2±1.0	39.60±0.06			
	60bp RNA (1 dye)		1.25 mM LiCl, pH=9.1	22.6	5.311	187.1±2.1	39.20±0.15			
NaCl	**30bp DNA**		**1.25 mM NaCl, pH=9.1**	**22.0**	**4.104**	**50.2±0.2**	**25.77±0.05**			
	**40bp DNA**	**2*h*=74.7 nm**	**1.24 mM NaCl, pH=9.2**	**22.0**	**4.602**	**94.0±1.0**	**31.06±0.14**	0.96	3.0	11.8
	**60bp DNA**	***d*=146 nm**	**1.25 mM NaCl, pH=9.2**	**22.3**	**5.396**	**256.4±0.9**	**41.09±0.04**			
	**60bp DNA (1 dye)**		**1.25 mM NaCl, pH=9.2**	**22.5**	**5.364**	**247.1±1.9**	**40.69±0.10**			
	30bp DNA		1.24 mM NaCl, pH=9.0	22.1	4.324	61.2±0.5	27.57±0.12			
	40bp DNA	2*h*=74.7 nm	1.22 mM NaCl, pH=9.0	21.8	4.863	116.6±0.4	33.11±0.04	0.98	3.1	13.0
	60bp DNA	*d*=146 nm	1.22 mM NaCl, pH=9.1	21.7	5.790	357.8±0.9	43.91±0.03			
	30bp DNA		1.10 mM NaCl, pH=9.1	21.7	4.800	93.2±1.1	26.61±0.12			
	40bp DNA	2*h*=74.7 nm	1.12 mM NaCl, pH=9.1	21.7	5.276	173.4±0.8	32.08±0.05			
	60bp DNA	*d*=146 nm	1.28 mM NaCl, pH=9.1	21.9	5.350	250.1±0.7	42.54±0.04	1.03	3.0	10.4
	60bp DNA (1 dye)		1.29 mM NaCl, pH=9.0	22.0	5.309	229.4±1.7	42.01±0.10			
	30bp DNA	2*h*=73.8 nm	1.30 mM NaCl, pH=9.1	22.7	4.154	31.9±0.1	26.19±0.02			
	40bp DNA	*d*=157 nm	1.30 mM NaCl, pH=9.2	23.0	4.589	54.1±0.2	31.49±0.07	0.85	3.1	16.9
	60bp DNA		1.31 mM NaCl, pH=9.1	22.9	5.358	141.2±0.9	41.81±0.09			
	**30bp RNA**		**1.26 mM NaCl, pH=9.1**	**22.2**	**4.622**	**45.6±0.2**	**26.46±0.06**			
	**40bp RNA**	**2*h*=71.5 nm**	**1.27 mM NaCl, pH=9.1**	**22.2**	**5.057**	**77.9±0.9**	**31.34±0.14**	1.04	2.4	13.5
	**60bp RNA**	***d*=150 nm**	**1.29 mM NaCl, pH=9.2**	**22.2**	**5.800**	**183.3±1.0**	**40.69±0.07**			
	30bp RNA		1.17 mM NaCl, pH=9.1	22.5	4.230	50.3±0.8	23.85±0.17			
	40bp RNA	2*h*=74.7 nm	1.17 mM NaCl, pH=9.1	22.6	4.657	86.3±0.9	28.48±0.12	0.93	2.4	13.9
	60bp RNA	*d*=146 nm	1.23 mM NaCl, pH=9.1	22.5	5.137	163.7±0.4	36.79±0.04			
	60bp RNA (1 dye)		1.23 mM NaCl, pH=9.1	22.7	5.077	152.1±1.2	36.34±0.10			
	30bp RNA		1.29 mM NaCl, pH=9.1	24.1	4.039	25.2±0.1	24.31±0.06			
	40bp RNA	2*h*=73.8 nm	1.28 mM NaCl, pH=9.2	23.9	4.438	42.8±0.3	28.83±0.07	0.93	2.4	14.5
	60bp RNA	*d*=157 nm	1.28 mM NaCl, pH=9.2	23.6	5.150	98.7±0.2	37.40±0.03			
RbCl	**30bp DNA**		**1.26 mM RbCl, pH=9.0**	**22.6**	**4.162**	**48.2±0.6**	**26.67±0.16**			
	**40bp DNA**	**2*h*=76.6 nm**	**1.26 mM RbCl, pH=8.9**	**22.6**	**4.549**	**83.2±1.7**	**31.94±0.34**	1.01	3.1	10.7
	**60bp DNA**	***d*=186 nm**	**1.26 mM RbCl, pH=8.9**	**22.8**	**5.369**	**240.1±1.0**	**42.65±0.06**			
	30bp DNA		1.21 mM RbCl, pH=8.8	22.9	4.210	50.6±0.7	25.30±0.21			
	40bp DNA	2*h*=76.6 nm	1.22 mM RbCl, pH=8.9	22.8	4.613	89.8±0.8	30.41±0.10	0.90	3.2	12.4
	60bp DNA	*d*=186 nm	1.20 mM RbCl, pH=8.9	22.9	5.500	271.3±1.7	40.45±0.08			
	* 30bp DNA		1.25 mM RbCl, pH=8.9	23.1	3.705	32.1±0.2	20.30±0.08			
	40bp DNA	2*h*=76.6 nm	1.25 mM RbCl, pH=9.0	22.9	4.072	54.8±0.1	24.19±0.02	0.77	3.0	10.9
	60bp DNA	*d*=186 nm	1.25 mM RbCl, pH=9.0	22.7	4.789	133.2±0.7	32.17±0.07			
	*** 30bp RNA**		**1.19 mM RbCl, pH=8.9**	**22.1**	**3.897**	**36.1±0.6**	**20.67±0.05**			
	**40bp RNA**	**2*h*=76.6 nm**	**1.20 mM RbCl, pH=8.9**	**22.1**	**4.212**	**55.0±0.5**	**24.44±0.10**	0.86	2.4	11.2
	**60bp RNA**	***d*=186 nm**	**1.21 mM RbCl, pH=8.9**	**22.5**	**4.853**	**120.9±0.4**	**31.78±0.06**			
	30bp RNA		1.23 mM RbCl, pH=8.9	22.8	4.068	43.3±1.9	24.37±0.54			
	40bp RNA	2*h*=76.6 nm	1.22 mM RbCl, pH=9.0	22.2	4.477	70.9±2.9	28.84±0.53	0.92	2.6	13.3
	60bp RNA	*d*=186 nm	1.22 mM RbCl, pH=9.0	22.5	5.132	165.0±0.5	37.46±0.04			
	* 30bp RNA		1.22 mM RbCl, pH=8.9	22.4	3.711	30.3±0.6	19.87±0.05			
	40bp RNA	2*h*=76.6 nm	1.21 mM RbCl, pH=9.0	22.7	4.105	40.9±0.5	23.44±0.12	0.71	2.5	16.7
	60bp RNA	*d*=186 nm	1.21 mM RbCl, pH=8.9	22.4	4.697	111.5±0.2	30.57±0.04			

Table continued from previous page

	Molecule	Device	Buffer 1 mM Tris +	T (°C)	W (k_BT)	t_{esc} (s)	q_m (-e)	f_M	Structural parameters b_m (Å)	Structural parameters r_m (Å)
CsCl	*** 30bp DNA**	**2h=73.4 nm**	**1.22 mM CsCl, pH=9.0**	**23.7**	**4.006**	**45.2±0.6**	**21.52±0.13**			
	**40bp DNA**	**d=144 nm**	**1.22 mM CsCl, pH=9.0**	**23.7**	**4.477**	**78.8±0.6**	**25.94±0.08**	0.81	3.2	10.4
	**60bp DNA**		**1.22 mM CsCl, pH=9.0**	**23.7**	**5.433**	**267.3±1.1**	**34.55±0.05**			
	* 30bp DNA	2h=76.6 nm	1.20 mM CsCl, pH=8.9	22.8	3.414	25.2±0.1	15.53±0.06			
	40bp DNA	d=186 nm	1.20 mM CsCl, pH=8.9	22.8	3.707	40.1±0.1	18.43±0.01	0.55	3.3	12.2
	60bp DNA		1.19 mM CsCl, pH=8.9	22.4	4.386	96.6±0.9	24.82±0.11			
	* 30bp DNA	2h=76.6 nm	1.21 mM CsCl, pH=8.9	23.0	3.664	29.5±0.4	18.62±0.20			
	40bp DNA	d=186 nm	1.21 mM CsCl, pH=8.8	22.9	4.031	49.8±0.2	22.48±0.05	0.64	3.1	14.7
	60bp DNA		1.21 mM CsCl, pH=8.9	22.5	4.755	129.1±0.6	29.78±0.05			
	*** 30bp RNA**	**2h=76.6 nm**	**1.20 mM CsCl, pH=9.0**	**22.8**	**3.538**	**26.7±0.3**	**16.95±0.15**			
	**40bp RNA**	**d=186 nm**	**1.20 mM CsCl, pH=9.0**	**22.6**	**3.839**	**39.9±0.1**	**20.08±0.04**	0.66	2.6	12.5
	**60bp RNA**		**1.18 mM CsCl, pH=9.1**	**23.0**	**4.486**	**89.3±0.2**	**26.26±0.04**			
	* 30bp RNA	2h=76.6 nm	1.20 mM CsCl, pH=8.9	22.7	3.442	24.9±0.1	16.09±0.05			
	40bp RNA	d=186 nm	1.20 mM CsCl, pH=9.0	22.9	3.749	36.7±0.2	19.15±0.08	0.63	2.5	13.0
	60bp RNA		1.19 mM CsCl, pH=9.0	22.9	4.333	80.8±0.6	24.90±0.10			
	* 30bp RNA	2h=75.2 nm	1.11 mM CsCl, pH=9.0	23.1	4.377	56.8±0.5	22.17±0.09			
	40bp RNA	d=167 nm	1.12 mM CsCl, pH=9.0	22.8	4.782	95.1±1.4	26.17±0.15	0.83	2.6	13.8
	60bp RNA		1.12 mM CsCl, pH=9.0	22.8	5.687	266.7±0.9	34.24±0.04			

Table S1. Experimental details on all r and b measurements. DNA datasets are highlighted in red. The measured b-r probability manifolds and inferred b_m and r_m values for datasets denoted in boldface are shown in Fig. S7. Datasets labeled with * were analyzed including the rotational free energy term, ΔF_{rot} (see section S2.2).

References

(1) Ruggeri, F.; Zosel, F.; Mutter, N.; Rozycka, M.; Wojtas, M.; Ozyhar, A.; Schuler, B.; Krishnan, M. Single-molecule electrometry. *Nature Nanotechnology* **2017**, *12* (5), 488-495. DOI: 10.1038/nnano.2017.26.
(2) Ruggeri, F.; Krishnan, M. Lattice diffusion of a single molecule in solution. *Physical Review E* **2017**, *96* (6), 062406. DOI: 10.1103/PhysRevE.96.062406.
(3) Demarest, S. J.; Martinez-Yamout, M.; Chung, J.; Chen, H. W.; Xu, W.; Dyson, H. J.; Evans, R. M.; Wright, P. E. Mutual synergistic folding in recruitment of CBP/p300 by p160 nuclear receptor coactivators. *Nature* **2002**, *415* (6871), 549-553. DOI: 10.1038/415549a.
(4) Borgia, A.; Zheng, W. W.; Buholzer, K.; Borgia, M. B.; Schuler, A.; Hofmann, H.; Soranno, A.; Nettels, D.; Gast, K.; Grishaev, A.; et al. Consistent View of Polypeptide Chain Expansion in Chemical Denaturants from Multiple Experimental Methods. *Journal of the American Chemical Society* **2016**, *138* (36), 11714-11726. DOI: 10.1021/jacs.6b05917.
(5) Tirado, M. M.; Garciadelatorre, J. Translational friction coefficients of rigid, symmetric top macromolecules. Application to circular cylinders. *Journal of Chemical Physics* **1979**, *71* (6), 2581-2587. DOI: 10.1063/1.438613. Tirado, M. M.; Martinez, C. L.; Delatorre, J. G. Comparison of theories for the translational and rotational diffusion coefficients of rod-like macromolecules. Application to short DNA fragments. *Journal of Chemical Physics* **1984**, *81* (4), 2047-2052. DOI: 10.1063/1.447827.
(6) Odijk, T. Polyelectrolytes near the rod limit. *Journal of Polymer Science Part B-Polymer Physics* **1977**, *15* (3), 477-483. DOI: 10.1002/pol.1977.180150307. Skolnick, J.; Fixman, M. Electrostatic Persistence Length of a Wormlike Polyelectrolyte. *Macromolecules* **1977**, *10* (5), 944-948. DOI: 10.1021/ma60059a011.
(7) Kovacic, R. T.; Vanholde, K. E. Sedimentation of Homogeneous Double-Strand DNA Molecules. *Biochemistry* **1977**, *16* (7), 1490-1498. DOI: 10.1021/bi00626a038. Kapahnke, R.; Rappold, W.; Desselberger, U.; Riesner, D. The stiffness of dsRNA: hydrodynamic studies on fluorescence-labelled RNA segments of bovine rotavirus. *Nucleic Acids Research* **1986**, *14* (8), 3215-3228. DOI: 10.1093/nar/14.8.3215.
(8) Kramers, H. A. Brownian motion in a field of force and the diffusion model of chemical reactions. *Physica* **1940**, *7*, 284-304. DOI: 10.1016/s0031-8914(40)90098-2.
(9) Krishnan, M. A simple model for electrical charge in globular macromolecules and linear polyelectrolytes in solution. *Journal of Chemical Physics* **2017**, *146* (20), 205101. DOI: 10.1063/1.4983485.
(10) Ruggeri, F.; Krishnan, M. Entropic Trapping of a Singly Charged Molecule in Solution. *Nano Letters* **2018**, *18* (6), 3773-3779. DOI: 10.1021/acs.nanolett.8b01011.
(11) Brown, M. A.; Bossa, G. V.; May, S. Emergence of a Stern Layer from the Incorporation of Hydration Interactions into the Gouy-Chapman Model of the Electrical Double Layer. *Langmuir* **2015**, *31* (42), 11477-11483. DOI: 10.1021/acs.langmuir.5b02389.
(12) Franks, G. V. Zeta potentials and yield stresses of silica suspensions in concentrated monovalent electrolytes: Isoelectric point shift and additional attraction. *Journal of Colloid and Interface Science* **2002**, *249* (1), 44-51. DOI: 10.1006/jcis.2002.8250. Redondo, A. B.; Jordan, I.; Ziazadeh, I.; Kleibert, A.; Giorgi, J. B.; Worner, H. J.; May, S.; Abbas, Z.; Brown, M. A. Nanoparticle-Induced Charge Redistribution of the Air-Water Interface. *Journal of Physical Chemistry C* **2015**, *119* (5), 2661-2668. DOI: 10.1021/jp511915b.
(13) Darlington, A. M.; Gibbs-Davis, J. M. Bimodal or Trimodal? The Influence of Starting pH on Site Identity and Distribution at the Low Salt Aqueous/Silica Interface. *Journal of Physical Chemistry C* **2015**, *119* (29), 16560-16567. DOI: 10.1021/acs.jpcc.5b02480.
(14) Pau, P. C. F.; Berg, J. O.; McMillan, W. G. Application of Stokes' law to ions in aqueous solution. *Journal of Physical Chemistry* **1990**, *94* (6), 2671-2679. DOI: 10.1021/j100369a080.
(15) Nightingale, E. R. Phenomenological Theory of Ion Solvation. Effective Radii of Hydrated Ions. *Journal of Physical Chemistry* **1959**, *63* (9), 1381-1387. DOI: 10.1021/j150579a011.
(16) Marcus, Y. Thermodynamics of solvation of ions. Part 5.—Gibbs free energy of hydration at 298.15 K. *Journal of the Chemical Society-Faraday Transactions* **1991**, *87* (18), 2995-2999. DOI: 10.1039/ft9918702995.
(17) Li, S. X.; Olson, W. K.; Lu, X. J. Web 3DNA 2.0 for the analysis, visualization, and modeling of 3D nucleic acid structures. *Nucleic Acids Research* **2019**, *47* (W1), W26-W34. DOI: 10.1093/nar/gkz394.
(18) Tomasello, G.; Armenia, I.; Molla, G. The Protein Imager: a full-featured online molecular viewer interface with server-side HQ-rendering capabilities. *Bioinformatics* **2020**, *36* (9), 2909-2911. DOI: 10.1093/bioinformatics/btaa009.
(19) Nguyen, H.; Case, D. A.; Rose, A. S. NGLview-interactive molecular graphics for Jupyter notebooks. *Bioinformatics* **2018**, *34* (7), 1241-1242. DOI: 10.1093/bioinformatics/btx789.
(20) Overbeek, J. T. G. The role of energy and entropy in the electrical double layer. *Colloids and Surfaces* **1990**, *51*, 61-75.
(21) Krishnan, M. Electrostatic free energy for a confined nanoscale object in a fluid. *Journal of Chemical Physics* **2013**, *138* (11), 114906. DOI: 10.1063/1.4795087.
(22) Lee, B.; Richards, F. M. The interpretation of protein structures: estimation of static accessibility. *Journal of Molecular Biology* **1971**, *55* (3), 379-400. DOI: 10.1016/0022-2836(71)90324-x. Richards, F. M. Areas, volumes,

packing, and protein structure. *Annual Review of Biophysics and Bioengineering* **1977**, *6*, 151-176. DOI: 10.1146/annurev.bb.06.060177.001055. Connolly, M. L. Analytical molecular surface calculation. *Journal of Applied Crystallography* **1983**, *16* (OCT), 548-558. DOI: 10.1107/s0021889883010985. Richmond, T. J. Solvent accessible surface area and excluded volume in proteins: Analytical equations for overlapping spheres and implications for the hydrophobic effect. *Journal of Molecular Biology* **1984**, *178* (1), 63-89. DOI: 10.1016/0022-2836(84)90231-6.
(23) Sanner, M. F.; Olson, A. J.; Spehner, J. C. Reduced surface: An efficient way to compute molecular surfaces. *Biopolymers* **1996**, *38* (3), 305-320. DOI: 10.1002/(sici)1097-0282(199603)38:3<305::aid-bip4>3.0.co;2-y.
(24) Pettersen, E. F.; Goddard, T. D.; Huang, C. C.; Couch, G. S.; Greenblatt, D. M.; Meng, E. C.; Ferrin, T. E. UCSF chimera - A visualization system for exploratory research and analysis. *Journal of Computational Chemistry* **2004**, *25* (13), 1605-1612. DOI: 10.1002/jcc.20084.
(25) Lu, X. J.; Olson, W. K. 3DNA: a software package for the analysis, rebuilding and visualization of three-dimensional nucleic acid structures. *Nucleic Acids Research* **2003**, *31* (17), 5108-5121. DOI: 10.1093/nar/gkg680.
(26) Tsai, J.; Taylor, R.; Chothia, C.; Gerstein, M. The packing density in proteins: Standard radii and volumes. *Journal of Molecular Biology* **1999**, *290* (1), 253-266. DOI: 10.1006/jmbi.1999.2829.
(27) Geuzaine, C.; Remacle, J. F. Gmsh: A 3-D finite element mesh generator with built-in pre- and post-processing facilities. *International Journal for Numerical Methods in Engineering* **2009**, *79* (11), 1309-1331. DOI: 10.1002/nme.2579.
(28) Martin S. Alnæs, J. B., Johan Hake, August Johansson, Benjamin Kehlet, Anders Logg, Chris Richardson, Johannes Ring, Marie E. Rognes, and Garth N. Wells. The FEniCS Project Version 1.5. *Archive of Numerical Software* **2015**, *3* (100), 9-23.
(29) Kirmizialtin, S.; Silalahi, A. R. J.; Elber, R.; Fenley, M. O. The Ionic Atmosphere around A-RNA: Poisson-Boltzmann and Molecular Dynamics Simulations. *Biophysical Journal* **2012**, *102* (4), 829-838. DOI: 10.1016/j.bpj.2011.12.055.
(30) Borukhov, I.; Andelman, D.; Orland, H. Steric effects in electrolytes: A modified Poisson-Boltzmann equation. *Physical Review Letters* **1997**, *79* (3), 435-438. DOI: 10.1103/PhysRevLett.79.435.
(31) Behjatian, A.; Krishnan, M. Electrostatic free energies carry structural information on nucleic acid molecules in solution. *Journal of Chemical Physics* **2022**, *156* (13). DOI: 10.1063/5.0080008.
